# Clinical decision thresholds for surfactant administration in preterm infants: a systematic review and network meta-analysis

**DOI:** 10.1016/j.eclinm.2023.102097

**Published:** 2023-07-20

**Authors:** Viraraghavan Vadakkencherry Ramaswamy, Tapas Bandyopadhyay, Thangaraj Abiramalatha, Abdul Kareem Pullattayil S, Tomasz Szczapa, Clyde J. Wright, Charles Christoph Roehr

**Affiliations:** a Department of Neonatology, Ankura Hospital for Women and Children, Hyderabad, India; b Department of Neonatology, ABVIMS and Dr. RML Hospital, New Delhi, India; c Department of Neonatology, Kovai Medical Center and Hospital (KMCH), Coimbatore, Tamil Nadu, India; d Queen’s University, Kingston, Canada; e II Department of Neonatology, Neonatal Biophysical Monitoring and Cardiopulmonary Therapies Research Unit, Poznan University of Medical Sciences, Poznan, Poland; f Section of Neonatology, Department of Pediatrics, Children's Hospital Colorado and University of Colorado School of Medicine, Aurora, Colorado, USA; g National Perinatal Epidemiology Unit, Nuffield Department of Population Health, Medical Sciences Division, University of Oxford, Oxford, United Kingdom; h Newborn Services, Southmead Hospital, North Bristol NHS Trust, Bristol, United Kingdom; i Faculty of Health Sciences, University of Bristol, Bristol, United Kingdom

**Keywords:** Surfactant, Neonate, Preterm, RDS

## Abstract

**Background:**

The ideal threshold at which surfactant administration in preterm neonates with respiratory distress syndrome (RDS) is most beneficial is contentious. The aim of this systematic review was to determine the optimal clinical criteria to guide surfactant administration in preterm neonates with RDS.

**Methods:**

The systematic review was registered in PROSPERO (CRD42022309433). Medline, Embase, CENTRAL and CINAHL were searched from inception till 16th May 2023. Only randomized controlled trials (RCTs) were included. A Bayesian random effects network meta-analysis (NMA) evaluating 33 interventions was performed. The primary outcome was requirement of invasive mechanical ventilation (IMV) within 7 days of life.

**Findings:**

58 RCTs were included. In preterm neonates ≤30 weeks after adjusting for the confounding factor of modality of surfactant administration, an arterial alveolar oxygen tension ratio (aAO_2_) <0.36 (FiO_2_: 37–55%) was ranked the best threshold for decreasing the risk of IMV, very low certainty. Further, surfactant administration at an FiO_2_ 40–45% possibly decreased mortality compared to rescue treatment when respiratory failure was diagnosed, certainty very low. The reasonable inference that could be drawn from these findings is that surfactant administration may be considered in preterm neonates of ≤30 weeks’ with RDS requiring an FiO_2_ ≥ 40%. There was insufficient evidence for the comparison of FiO_2_ thresholds: 30% vs. 40%. The evidence was sparse for surfactant administration guided by lung ultrasound. For the sub-group >30 weeks, nebulized surfactant administration at an FiO_2_ < 30% possibly increased the risk of IMV compared to Intubate-Surfactant-Extubate at FiO_2_ < 30% and 40%, and less invasive surfactant administration at FiO_2_ 40%, certainty very low.

**Interpretation:**

Surfactant administration may be considered in preterm neonates of ≤30 weeks’ with RDS if the FiO_2_ requirement is ≥40%. Future trials are required comparing lower FiO_2_ thresholds of 30% vs. 40% and that guided by lung ultrasound.

**Funding:**

None.

**Research in context**

**Evidence before this study**

A literature search of the databases Medline and Embase using the key words ‘surfactant’, ‘respiratory distress syndrome’ and ‘preterm’ was performed from 1st January 2000 to 16th May 2023. Only systematic reviews and randomized controlled trials which had evaluated surfactant administration in preterm infants were included. Studies published in both English and non-English languages were eligible for inclusion and descriptive reviews were excluded. Three network meta-analyses (NMAs) related to surfactant administration in preterm neonates with RDS were identified. One NMA which focussed on various non-invasive respiratory support modalities in preterm infants such as CPAP, non-invasive positive pressure ventilation along with different surfactant administration modalities such as INSURE, LISA and through LMA reported that LISA was associated with the lowest likelihood of mortality or BPD at 36 weeks’ postmenstrual age (OR, 0.49; 95% CrI, 0.30–0.79), with the evidence quality being low. The other NMA exclusively evaluated the various modalities of surfactant administration in preterm infants and found that surfactant administration via thin catheters was associated with lower likelihood of mortality (OR, 0.64; 95% CI, 0.54–0.76), need for mechanical ventilation (OR, 0.43; 95% CI, 0.29–0.63) and bronchopulmonary dysplasia (OR, 0.57; 95% CI, 0.44–0.73) with the evidence quality being moderate. The most recently published NMA studied the different FiO_2_ thresholds for surfactant administration in preterm neonates of less than 32 weeks’ gestation with RDS and found that there was no clear benefit of surfactant therapy based on different FiO_2_ cut-off levels with the quality of evidence being low.

**Added value of this study**

This NMA investigated the various thresholds utilized for surfactant administration. It provides a holistic approach of evaluating surfactant administration in preterm neonates with RDS by studying a range of techniques, thresholds including but not merely focussing on FiO_2_ alone, and respiratory support modes. By including the technique of surfactant administration as a part of intervention we are cautiously confident to have circumvented the risk of intransitivity, which is the fundamental assumption of an NMA analysis. This novel approach of combining the two important aspects related to surfactant administration has not been utilized in the previously published NMAs. The interpretation of the results of this NMA paves way for clinically relevant conclusions and henceforth should help guide safe clinical practice.

**Implications of all the available evidence**

The ideal threshold for surfactant administration in preterm neonates with RDS may not be restricted to the FiO_2_ requirement alone but may also be related to other criteria such as a/AO_2_ ratio, clinical assessment of respiratory distress, severity of RDS as adjudged by lung ultrasound and lamellar body counts. Based on the results of this NMA, in preterm neonates of ≤30 weeks’ gestation, surfactant administration may be considered when an FiO_2_ threshold of 40% is reached. Clinicians may take into consideration many of the other aforementioned parameters while adjudging the requirement of surfactant replacement therapy in preterm neonates with RDS as well. Such an approach might identify preterm neonates with RDS who could benefit from surfactant administration earlier and hence improve clinical outcomes. Finally, this systematic review also identifies gaps in current literature to stimulate discussion and advise future research into interventions for which the evidence certainty is very low to low, such as surfactant guided by lung ultrasound severity scores.

## Introduction

Surfactant administration for the treatment of respiratory distress syndrome (RDS) is the mainstay intervention to reduce mortality and major morbidity in preterm neonates.^1^ Since the first clinical trial performed in 1980, significant progress has been made with respect to its use.^2^ Systematic reviews indicate that early surfactant administration and the use of lesser invasive modalities such as less invasive surfactant administration (LISA) reduce mortality and the risk of bronchopulmonary dysplasia (BPD). However, there is a paucity of evidence to define the optimal thresholds at which exogenous surfactant therapy would be most effective.^1^^,^^3^, ^4^, ^5^

Our current understanding is that the pathophysiology of RDS is multifactorial, of which surfactant deficiency remains the most important aspect.^6^ In the absence of a clinically feasible test to accurately and timely quantify surfactant levels in the preterm lung, several other markers have been used to classify the severity of RDS. Of these, the fraction of inspired oxygen (FiO_2_) requirement is most used. To date, there are no randomized controlled trials (RCT) evaluating different FiO_2_ thresholds together with defined modalities of surfactant administration in preterm neonates with RDS who are stabilized on CPAP.^7^^,^^8^ This lacuna is evident in the differing recommendations by various international RDS treatment guidelines, focusing predominantly on oxygen requirement to maintain acceptable peripheral saturations (SpO_2_).^3^^,^^9^ However, apart from FiO_2_ there may be other parameters available to predict significant RDS and guide targeted surfactant administration, ideally during an optimal time frame. Other parameters for predicting the severity of RDS that have been studied include arterial alveolar oxygen tension ratio (a/AO_2_), lamellar body counts in lung- or gastric aspirates, lung ultrasound and clinical scoring systems of respiratory distress.^8^^,^^10^, ^11^, ^12^

This systematic review was performed with an aim to assess the different thresholds for surfactant administration in preterm neonates (≤36 weeks’) diagnosed with or at risk of RDS. A network meta-analysis (NMA) was used to synthesize data as it allows for comparing efficacies as well as ranking these through the provision of indirect evidence as there are only a few RCTs comparing the various decision thresholds for surfactant administration.^13^

## Methods

The systematic review was registered in PROSPERO (CRD42022309433).^14^ The results of the NMA was reported according to PRISMA-NMA.^15^

### Search strategy

**Fig. 1:**
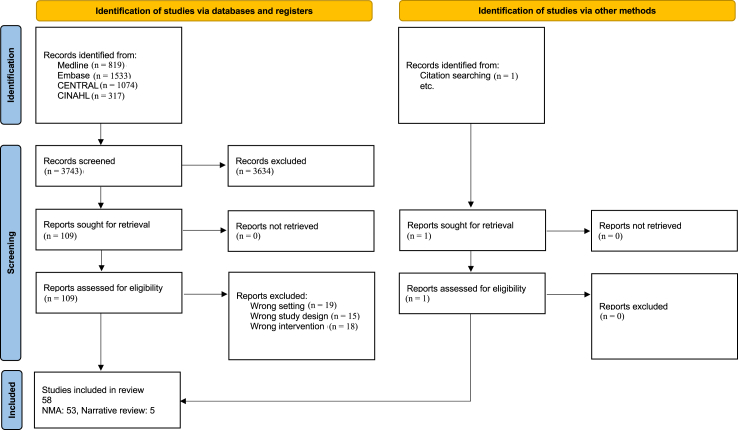
PRISMA flow of literature search.

Medline, Embase, CENTRAL and CINAHL were searched by two authors independently without any restrictions^16^ (eTable S1). A balance of recall and precision was maintained while searching all the databases. The PRISMA flowchart is given in Fig. 1.

### Selection criteria

#### Patient population

Preterm neonates (born at ≤36 weeks’ gestation) and diagnosed with or at risk of RDS.

#### Interventions/comparators

33 interventions were evaluated. Each intervention included two components: The threshold used for surfactant administration and the modality by which surfactant was administered (Annexure 1). The modality of surfactant administration was combined along with the treatment threshold to avoid confounding and intransitivity.

#### Outcomes

The primary outcome was requirement of invasive mechanical ventilation (IMV) within the first week of life. The secondary outcomes included mortality before discharge, mortality or BPD (oxygen requirement at 36 weeks’ postmenstrual age), intraventricular hemorrhage grade (IVH) > grade 2,^17^ air leak, receipt of multiple doses of surfactant and long term neurodevelopmental outcomes.

#### Study selection

Only RCTs were included.

#### Time frame

The databases were searched from inception until 16th May 2023.

### Risk of bias assessment

The risk of bias of the included studies was evaluated using the Cochrane risk of bias tool version 2.0 by two authors independently.^18^ Disagreements were resolved by consensus.

### Certainty of evidence (CoE)

CoE for the NMA effect estimates was assessed according to GRADE recommendations.^19^ The CoE is classified into 4 categories: very low, low, moderate and high. Whilst very low and low CoE indicate that the true effect probably or might be different from the estimated effect, moderate CoE suggest that the true effect is probably close to the estimated effect. High CoE means that the authors are very confident that the true effect is similar to the estimated effect.

### Statistical analysis

A Bayesian NMA was performed using the R-software (R Foundation for Statistical Computing, Vienna, Austria) and the “gemtc” package was utilized for data synthesis.^20^ The “netmeta” package was utilized for pairwise meta-analysis.^20^ Markov chain Monte Carlo (MCMC) simulation using vague priors with four chains, burn-in of 50,000 iterations, followed by 10,00,000 iterations and 10,000 adaptations was used. Model convergence was assessed using Gelman–Rubin Potential Scale Reduction Factor, trace and density plots. Leverage plots, total residual deviance and deviance information criterion were evaluated to confirm model convergence. Intransitivity was evaluated by comparing the characteristics of the included the studies and inconsistency by node splitting. Pair-wise meta-analysis of RCTs was also performed. The effect estimates of the NMA were reported as risk ratio (RR) with 95% credible interval (CrI). The NMA estimates were illustrated with forest plots and matrix plots. Surface under the cumulative ranking curve (SUCRA) was used to depict the ranking of the interventions.^21^ Publication bias in direct evidence from meta-analyses of RCTs would be assessed if at least 10 trials had evaluated an outcome. When the data from the trials could not be synthesized in an NMA, they were described in the narrative review.

### Sub-group analyses/meta-regression

Sub-groups analyses were performed based on gestational age: ≤30 weeks and >30 weeks. Meta-regression was done for the following gestational ages: 24 weeks, 26 weeks, 28 weeks, 30 weeks, 32 weeks, and 36 weeks.

### Role of funding source

There was no funding obtained for this study. All the authors of this paper namely, Viraraghavan Vadakkencherry Ramaswamy, Tapas Bandyopadhyay, Thangaraj Abiramalatha, Abdul Kareem Pullattayil, Tomasz Szczapa, Clyde J. Wright and Charles Christoph Roehr have access to the data set and all of them had made the decision to submit for publication.

## Results

After the removal of duplicates, 3661 titles and abstracts were screened. Of these, 108 full texts and 1 abstract were retrieved. 58 studies were included in the systematic review: 53 studies analyzed in the NMA^8^^,^^10^^,^^11^^,^^22^, ^23^, ^24^, ^25^, ^26^, ^27^, ^28^, ^29^, ^30^, ^31^, ^32^, ^33^, ^34^, ^35^,^36^, ^37^, ^38^, ^39^, ^40^, ^41^, ^42^, ^43^, ^44^, ^45^,^46^, ^47^, ^48^, ^49^, ^50^, ^51^, ^52^, ^53^, ^54^, ^55^, ^56^, ^57^, ^58^, ^59^, ^60^,^61^, ^62^, ^63^, ^64^, ^65^, ^66^, ^67^, ^68^, ^69^, ^70^, ^71^ and 5 in the narrative review.^72^, ^73^, ^74^, ^75^, ^76^ The characteristics of the included studies is given in Table 1.

**Table 1:** Characteristics of included studies.

Study	n	Mean GA (w)	Mean BW (g)	Respiratory support	Surfactant threshold	Surfactant: type, dosage	Modality of administration	Criteria for IMV	Criteria for repeat surfactant	ANS (%)	Other comments
Attridge 2012 USA	I: 13 C: 13	I: 33 C: 32	I: 2001 C: 2130	nCPAP	- FiO_2_: 30–60%	Calf lung surfactant	I: LMA C: INSURE	NA	- FiO_2_ rise >10% for >30 min after the initial dose	I: 54 C: 46	- Both group received surfactant via ET tube if FiO_2_ rise >15% for >30 min after enrolment - FiO_2_ at randomisation in intervention group and control group are 0.39 (0.33–0.45) and 0.38 (0.34–0.40) respectively
Amini 2019 Iran	I: 30 C: 30	I: 32.6 C: 32.4	I: 1970 C: 1850	nCPAP+/− NIPPV	- FiO_2_ was 20% more than the baseline - FiO_2_ was ≥60% - FiO_2_ ≥ 30% associated with the worsening of ACORN score	Poractant	I: LMA C: INSURE	- Severe respiratory distress: defined as ACORN >8 - Severe acid-base imbalance based on arterial blood gas (ABG) - Apnea during the first 24 h of life	- Same as for the initial dose	I: 90 C: 86.7	- 13.3% vs. 6.7%, p = 0.67 required a second dose of surfactant within 12 h of the first, while no neonate in either group required a third dose of surfactant administration
Berggren 2000 Sweden	I: 16 C: 16	I: 31 C: 31	I: 1620 C: 1603	nCPAP	- FiO_2_ > 40% - a/AO_2_ < 0.22 - Clinically and radiologically diagnosed progressive RDS	Poractant 480 mg	I: Nebulisation C: CPAP only	- a/AO_2_ < 0.15	NA	I: 81 C: 69	- No improvement in pH or a/A PO_2_ over time. No difference in any other outcomes.
Bao 2015 China	I: 47 C: 43	I: 29.1 C: 29.3	I: 1034 C: 1087	nCPAP	- FiO_2_ > 30% - Clinically (Silverman-Anderson score greater than 4) and/or respiratory frequency >60 breaths per minute and radiologically diagnosed RDS	Poractant 200 mg/kg	I: LISA C: INSURE	- FiO_2_ was >60% - Respiratory acidosis (pH < 7.2) - Significant apnea	- If clinically indicated	I: 89.4 C: 93	- No difference in any outcome except for significant reduction in duration of respiratory support in intervention group. - More surfactant reflux in intervention group. - Less fluctuation in vital parameters in intervention than control group.
Barbosa 2017 Brazil	I: 26 C: 22	I: 31.1 C: 31.4	I: 1515 C: 1495	nCPAP	- FiO_2_ ≥ 40% - Clinically (Silverman-Anderson score >4 and/or respiratory frequency >60 breaths per minute) and radiologically diagnosed RDS	Poractant 200 mg/kg	I: LMA C: IMV	- Clinician discretion	- Any infant 6–33 h after first dose presenting with: increasing respiratory effort, hemodynamic instability, frequent apnea (≥2/h), pH < 7.20, PaCO_2_ > 65 mmHg, PaO_2_ < 50 mmHg, SpO_2_ < 91%, or FiO_2_ ≥ 50% - LMA patients received a second dose by ET tube followed by IMV.	I: 53.8 C: 77.2	- No difference in primary outcome of reduction in FiO_2_ ≤ 30% 3 h after surfactant administration and number of surfactant doses - IMV duration was significantly higher in intervention group who were intubated in comparison to control group
Boskabadi 2019 Iran	I: 20 C: 20	I: 29.1 C: 28.2	I: 1280 C: 1230	nCPAP	- FiO_2_ > 50% - pCO_2_ > 50–60 mmHg and pH < 7.2	Poractant 200 mg/kg	I: LISA C: INSURE	NA	NA	NA	- Significant reduction in IMV in the first 72 h of life in the intervention arm. No difference in other outcomes.
Choupani 2018 Iran	I: 52 C: 52	I: 32.9 C: 33.1	I: 1938 C: 2067	nCPAP	- FiO_2_ > 40% - Presence of moderate to severe respiratory distress	Poractant 200 mg/kg	I: LISA C: INSURE	- SPO_2_ < 90% despite FiO_2_ 40–70% and CPAP of 5–10 cm H_2_O - pH less than 7.2 - PaCO_2_ ≥ 60 mmHg - Occurrence of persistent apnea	- 12 h after surfactant administration, if the FiO_2_ ≥ 30% to keep SpO_2_ > 86%	I: 28.8 C: 28.8	- No difference in the primary outcome of IMV requirement within the first 72 h and other secondary outcomes except for a higher incidence of hypoxemia in the control arm during the procedure
Dani 2004 Italy	I: 13 C: 14	I: 29 C: 28.3	I: 1078 C: 1126	nCPAP	- FiO_2_ ≥ 30% - Clinically and radiologically diagnosed RDS	Poractant 200 mg/kg	I: INSURE C: IMV	- pH < 7.20, PaO_2_ < 50 mm Hg with FiO_2_ > 50% and PCo_2_ > 65 mm Hg	- FiO_2_ > 50% 12 h after 1st dose	I: 62 C: 93	- Significant reduction in the requirement of IMV in the first 7 days, duration of oxygen use, duration of respiratory support, second dose of surfactant, duration of hospital stay in the intervention group
Dargaville 2021 Australia	I: 241 C: 244	I: 27.3 C: 27.3	I: 929 C: 928	nCPAP	- FiO_2_ ≥ 30% for LISA - FiO_2_ ≥ 45% once intubated	Poractant 200 mg/kg	I: LISA C: INSURE	- FiO_2_ ≥ 45% or severe or recurrent apnea or persistent respiratory acidosis	NA	I: 90.8 C: 92.2	- No difference in primary outcome of mortality or BPD at 36 weeks’ PMA - Significant reduction in BPD at 36 weeks’ PMA, pneumothorax requiring drainage, and need for intubation within 72 h, intubation at any time, requirement of surfactant therapy by endotracheal tube, significant reduction in duration of all forms of respiratory support in the intervention group
Dilmen 2013 Turkey	I: 79 C: 80	I: 28.7 C: 28.3	I: 1157 C: 1113	nCPAP	- FiO_2_ > 40% for early group - FiO_2_ > 40% along with clinic-radiological evidence of RDS for late group	Poractant 200 mg/kg	INSURE for both groups	- Apnea unresponsive to stimulation and other medical management, pH < 7.25 with PaCO_2_ > 60 mm Hg, unresponsive metabolic acidosis, or FiO_2_ > 60% to maintain SpO_2_ ≥ 88% after 1 h of surfactant treatment or needing FiO_2_ ≥ 45%.	- FiO_2_ > 40% to maintain SpO_2_ ≥ 88% between 6 and 24 h of the surfactant administration	I: 67.9 C: 62.7	- No difference in outcomes except for a significantly lower risk of pneumothorax in intervention arm - Similar findings were observed when stratified according to the birthweight (<1000 g vs. ≥1000 g)
Dunn 1991 Canada	I: 62 C: 60	I: 26.9 C: 27.2	I: 962 C: 986	IMV	- Prophylactic for early group. - Clinico-radiological evidence for late group	Bovine lung surfactant 100 mg/kg	IMV in both groups	NA	- FiO_2_ increases by 10% over the lowest baseline value	I: 46.7 C: 50	- No difference in oxygenation and ventilation parameters between both groups. However, a/AO_2_ was better in the early group at 24 and 48 h - No difference in incidence of other neonatal complications
Egberds 1993 Netherlands	I: 75 C: 72	I: 28 C: 27.8	I: 1033 C: 1126	IMV	- Prophylactic for early group. - FiO_2_ ≥ 60% at 6–24 h after birth	Poractant 200 mg/kg	IMV in both groups	NA	- Only to the prophylactic group requiring >60% FiO_2_ at 6 h after first dose	I: 25 C: 32	- Significant reduction in tcPO_2_/FiO_2_ at 6 h. The reduction at 6 h was significantly higher for GA 26–27 weeks’ and BW < 1000 g vs. 28–29 weeks’ and ≥1000 g
Gharehbaghi 2018 Iran	I: 25 C: 25	I: 32.9 C: 33.8	I: 2078 C: 2198	nCPAP	- Clinico-radiological evidence of RDS	Bovine lung surfactant 100 mg/kg	I: LMA C: INSURE	- Clinical deterioration, respiratory acidosis and recurrent apnea	NA	NA	- No difference in any of the clinical outcomes
Gopel 2011 Germany	I: 108 C: 112	I: 27.6 C: 27.5	I: 975 C: 938	nCPAP	- FiO_2_ > 30%	Porcine/Bovine lung surfactant 100 mg/kg	I: LISA C: INSURE	- Severe respiratory distress/asphyxia - High FiO_2_ (centre-specific) ranging from 30% to 60% - Low pH (centre-specific) ranging from 7.15 to 7.20 - High pCO_2_ (centre-specific) ranging from 60 to 70 mm Hg	- FiO_2_ > 40%	I: 96 C: 96	- Significant reduction in primary outcome of any requirement of IMV overall as well as in subgroup of neonates at 28 weeks’ gestation in the intervention arm - Significant reduction in requirement of any IMV, duration of IMV, supplemental oxygen requirement at 28 days, requirement of sedatives and analgesics in the intervention arm
Gortner 1998 Germany	I: 154 C: 163	I: 29.5 C: 29.7	I: 1227 C: 1269	nCPAP	- I: If requiring IMV - C: IMV with FiO_2_ ≥ 40%	Bovine lung surfactant 100 mg/kg	IMV in both groups	- Necessity of resuscitation and—severe RDS with impaired gas exchange: FiO_2_ > 50% or PaCO_2_ > 60 mm Hg or pH < 7.25 during spontaneous respiration	- FiO_2_ > 50%	I: 79.9 C: 72.8	- No difference in any of the studied outcomes
Gupta 2020 India	I: 29 C: 29	I: 30 C: 29.9	I: 1225 C: 1222	NIPPV	- FiO_2_ > 30%	Poractant 200 mg/kg	I: LISA C: INSURE	- Persistent respiratory acidosis with pH < 7.2 and pCO_2_ > 60 mm Hg or—recurrent apnoea requiring PPV or -NIPPV setting of FiO_2_ > 60%, PIP > 25 cm H_2_O and PEEP > 6 cm H_2_O	- FiO_2_ > 30%	I: 79.3 C: 82.8	- No difference in any of the studied outcomes
Gallup 2021 USA	I: 50 C: 40	NA	NA	NIPPV	- FiO_2_ 30–60%	NA	I: LMA C: INSURE	NA	NA	NA	- No difference in any of the studied outcomes
Han 2020 China	I: 151 C: 147	I: 30.6 C: 30.8	I: 1428 C: 1419	nCPAP	- FiO_2_ > 40%	Calf pulmonary surfactant 70–100 mg/kg	I: LISA C: INSURE	NA	- FiO_2_ > 40%	I: 72.9 C: 67	- No difference in any of the studied outcomes
Heidarzadeh 2013 Iran	I: 38 C: 42	I: 30.1 C: 29.6	I: 1490 C: 1383	nCPAP	- FiO_2_ > 30%	Poractant 200 mg/kg	I: LISA C: INSURE	NA	NA	I: 74 C: 74	- More episodes of desaturations in INSURE group - Higher incidence of NEC ≥ stage 2 and total duration of CPAP in INSURE group
Hentschel 2009 Germany	I: 77 C: 90	NA	NA	nCPAP	- I: If requiring IMV - C: IMV with FiO_2_ ≥ 40%	Bovine lung surfactant 100 mg/kg	IMV in both groups	- Necessity of resuscitation and—severe RDS with impaired gas exchange: FiO_2_ > 50% or PaCO_2_ > 60 mm Hg or pH < 7.25 during spontaneous respiration	- FiO_2_ > 50%	NA	Neuro-behavioural and motor development was similar in both groups as assessed by Griffiths scales, as was other morbidity. However, in the early treatment group. There was a delay in the subscale ‘personal social’ of the Griffiths test (p = 0.02), in ‘rolling over from supine to prone’ (p = 0.01) and an increased risk of raised muscular tone (p = 0.01) in the early treatment group.
Herting 2020 Germany	NA	NA	NA	nCPAP	- FiO_2_ > 30%	Porcine/Bovine lung surfactant 100 mg/kg	I: LISA C: INSURE	- Severe respiratory distress/asphyxia - High FiO_2_ (centre-specific) ranging from 30% to 60% - Low pH (centre-specific) ranging from 7.15 to 7.20 - High pCO_2_ (centre-specific) ranging from 60 to 70 mm Hg	- FiO_2_ > 40%	NA	No statistically significant differences in weight, length or neurodevelopmental outcome (Bayley II scores) were found between the LISA group and the control group at 2 years’ CA
Imani 2013 Iran	I: 40 C: 40	I: 30.4 C: 30.4	I: 1357 C: 1357	nCPAP	- Clinical assessment of respiratory distress	Poractant 100 mg/kg	I: CPAP C: INSURE	- Severe episodes of apnea - pH < 7.20 due to respiratory acidosis and a/AO_2_ < 0.15	NA	I: 69 C: 67	- No difference in any of the clinical outcomes
Jena 2019 India	I: 175 C: 175	I: 31 C: 31	I: 1630 C: 1683	nCPAP	- FiO_2_ > 30%	Neosurf 135 mg/kg	I: LISA C: INSURE	Clinical assessment and CXR based severity	- FiO_2_ > 30%	I: 61 C: 63	- Significant reduction in IMV in first 72 h, duration of CPAP, supplemental oxygen, NEC, BPD and duration of hospital stay - No significant reduction in other outcomes
Kanmaz 2013 Turkey	I: 100 C: 100	I: 28 C: 28.3	I: 1093 C: 1121	nCPAP	- FiO_2_ ≥ 40%	Poractant 100 mg/kg	I: LISA C: INSURE	- CPAP pressure >7 cm H_2_O along with FiO_2_ 60% - Sustained respiratory acidosis (pH < 7.2), and apnea requiring repeated PPV.	- FiO_2_ > 40% - PCO_2_ > 60 mm Hg	I: 73 C: 81	- Significant reduction in IMV in first 72 h, duration of CPAP and IMV - No difference in other clinical outcomes
Kribs 2015 Germany	I: 107 C: 104	I: 25.3 C: 25.2	I: 711 C: 674	I: nCPAP C: IMV	- FiO_2_ > 30% and/or Silverman Anderson score ≥5	Poractant 100 mg/kg	I: LISA C: IMV	- FiO_2_ > 45% for >2 h during CPAP to maintain PaO_2_ > 45 mmHg - Respiratory acidosis with pH < 7.15, or—severe apnea during CPAP despite respiratory analeptic therapy	- FiO_2_ > 35%	I: 82.2 C: 76	- No difference in the primary outcome of mortality or BPD at 36 weeks’ PMA, but significant reduction in the requirement of IMV in GA 25 and 26 weeks’, survival without major complications, duration of IMV, pneumothorax and IVH grade >2
Kandraju 2013 India	I: 74 C: 79	I: 30 C: 30	I: 1220 C: 1290	nCPAP	- I: Clinico-radiological RDS - C: FiO_2_ > 50%	Porcine/Bovine surfactant 100 mg/kg	INSURE in both groups	- Requirement of CPAP pressure >7 cm H_2_O and/or FiO_2_ > 70% to maintain SpO_2_ > 85% - PaCO_2_ > 55 mm Hg with arterial pH < 7.20 in association with increasing RD as measured by SAS - Recurrent/severe apnea defined as >4 episodes per hour or need for bag and mask ventilation >2 times per hour.	- FiO_2_ > 50%	I: 70 C: 74	- Significant reduction in the primary outcome of IMV requirement in the first 7 days of life - No difference in any other clinical outcomes
Kendig 1991 USA	I: 235 C: 244	I: 27.4 C: 27.5	I: 1023 C: 1040	IMV	- I: Prophylactic - C: Clinico-radiological evidence of RDS along with either FiO_2_ ≥ 40% on IMV with MAP > 7 cm H_2_O or both	Calf lung surfactant 90 mg	IMV in both groups	NA	- FiO_2_ ≥ 40% or IMV with MAP > 7 cm H_2_O or both	I: 66 C: 84	- Significant improvement in survival in the prophylactic group, particularly in neonates ≤26 weeks’ GA - Significant reduction in the incidence of pneumothorax in the prophylactic group - No difference in other outcomes
Khosravi 2008 Iran	I: 22 C: 23	I: 31.8 C: 31.3	I: 1642 C: 1695	NA	- Clinico-radiological evidence of RDS along with FiO_2_ > 40%	Bovine lung surfactant 70 mg/kg	I: INSURE C: IMV	NA	- FiO_2_ > 40%	I: 30.4 C: 36.3	- Significant reduction in duration of IMV in the first 24 h - No difference in other clinical outcomes
Kong 2016 China	I: 116 C: 91	NA	I: 1489 C: 1443	IMV	- Clinico-radiological evidence of RDS	Bovine lung surfactant 100 mg/kg	IMV in both groups	NA	- No significant improvement in respiratory symptoms observed after the first dose and CXR indicated no remission or worsened RDS - IMV was required and a PaO_2_ > 50 mmHg could only be maintained with FiO_2_ ≥ 40% and MAP ≥ 9 cm H_2_O - a/AO_2_ < 0.22	I: 84 C: 67	- Significant reduction in the incidence of severe RDS, requirement of repeat dose of surfactant, BPD, and PDA. The prophylactic group also has shorter duration of IMV and supplemental O_2_.
Konishi 1992 Japan	I: 16 C: 16	I: 27.3 C: 27.1	I: 1011 C: 1014	IMV	- I: Prophylactic - C: RDS on chest X-ray and FiO_2_ ≥ 40% and/or MAP > 7 cm H_2_O while on IMV	Surfactant-TA 100 mg/kg	IMV in both groups	NA	NA	I: 0 C: 6	- Significant reduction in the incidence of BPD in prophylactic group
Lefort 2003 Brazil	I: 35 C: 40	I: 29.6 C: 30.1	I: 1275 C: 1369	IMV	- I: Prophylactic - C: Clinico-radiological diagnosis and need for IMV, with FiO_2_ > 40% and PaO_2_/FiO_2_ ratio of ≤175 mmHg	Poractant 100 mg/kg	IMV in both groups	NA	NA	NA	- Significantly higher PaO_2_/FiO_2_ and PaO_2_/PAO_2_ in the prophylactic group - Significantly lower FiO_2_, PaO_2_ and D (A - a)O_2_ - No difference in common neonatal complications
Li 2016 China	I: 22 C: 22	I: 29.5 C: 29.3	I: 1089 C: 1145	nCPAP	- Clinical assessment of respiratory distress	Poractant	I: LISA C: INSURE	NA	NA	I: 72.7 C: 77.3	- No significant differences in the mean cerebral ScO_2_ and MABP - During surfactant administration rScO2-MABP significantly increased than baseline values in LISA group and INSURE group - In the first and second 5 min after administration, rScO2-MABP was not significantly different from baseline in the LISA group as compared to significant increase in the INSURE group in first 5 min after administration and returned to baseline in second 5 min - The relative change rate of rScO2-MABP in the INSURE group significantly exceeded that in the LISA group
Merritt 1991 USA	I: 76 C: 72	I: 26.9 C: 27.5	I: 1002 C: 1005	IMV	- I: Prophylactic. - C: Late rescue: Clinico-radiological evidence of RDS and FiO_2_ ≥ 50% and a MAP ≥ 7 cm H_2_O	Human surfactant 70 mg/kg	IMV in both groups	NA	- FiO_2_ was ≥50% or the MAP was ≥2 cm above the lowest level achieved after initial treatment	NA	- Significant reduction in PIE in prophylaxis as compared to placebo - No differences in other clinical outcomes except that ROP stage >1 occurred frequently among infants receiving prophylactic treatment - Significant reduction in mortality in the surfactant group as compared to placebo - No difference in other clinical outcomes
Minocchieri 2019 Australia	I: 32 C: 32	I: 31.4 C: 31.4	I: 1645 C: 1562	nCPAP	- I: FiO_2_ 22–30% - C: FiO_2_ > 35% over >30 min or FiO_2_ > 45% at anytime.	Poractant 200 mg/kg	I: Nebulised surfactant C: IMV	- FiO_2_ > 35% for >30 min or FiO_2_ > 45% at anytime - More than four episodes of apnea/hour or two episodes of apnea requiring bag and mask ventilation. - Two capillary blood gas samples with a pH < 7.20 and PaCO_2_ > 65 mm Hg (or PaCO_2_ > 60 mm Hg if arterial blood gas sample). - Physician discretion.	- Persisting oxygen requirement and/or respiratory distress (persistent tachypnea >60 breaths/min, sternal or intercostal recession, or grunting)	I: 90.6 C: 96.9	- Significant reduction in rate of CPAP failure within 72 h in 32–33^+6^ weeks with no difference in 29–31^+6^ weeks.
Mehler 2020 Germany	I: 78 C: 78	I: 25.3 C: 25.2	I: 711 C: 674	nCPAP	- FiO_2_ > 30%	Poractant 100 mg/kg	I: LISA C: INSURE	- FiO_2_ > 45% for >2 h during CPAP to obtain a PaO_2_ > 45 mmHg - Respiratory acidosis with pH < 7.15, or—severe apnea during CPAP despite respiratory analeptic therapy	- FiO_2_ > 35%	I: 82.2 C: 76	- Significantly higher MDI scores in the intervention group in babies between 25 and 26 weeks’ gestation. - Significant reduction in severe disability in both MDI and PDI among babies 25–26 weeks in the intervention group - Significant reduction in severe disability in PDI among all babies
Mohammadizadeh 2014 Iran	I: 19 C: 19	I: 30 C: 31	I: 1289 C: 1428	nCPAP	- FiO_2_ > 30% or—moderate to severe respiratory distress as defined as a Silverman Andersen score >4	Poractant 200 mg/kg	I: LISA C: INSURE	- FiO_2_ ≥ 70% for >2 h or >40% >12 h (despite surfactant administration). - pH < 7.2 or PaCO_2_ more than 65 mmHg. - One episode of severe apnea (requiring bag and mask ventilation) or six episodes of minor apnea (requiring only a brief stimulation) within 6 h	- FiO_2_ > 30%	I: 84.2 C: 89.5	- Significant reduction in the duration of oxygen therapy and the number of adverse events during surfactant administration
Nayeri 2014 Iran	I: 21 C: 21	I: 30.3 C: 31	I: 1485 C: 1532	I: nCPAP C: IMV	- FiO_2_ ≥ 45%	Bovine lung surfactant 100 mg/kg	I: INSURE C: IMV	- SPO_2_ < 85% despite FiO_2_ ≥ 70% and PEEP >7 cm H_2_O - Prolonged (>15 s) or recurrent (>2 episodes within 24 h) of apnea with bradycardia, requiring bag and mask ventilation - Respiratory acidosis with PaCO_2_ > 60 mmHg and PH < 7.2 in ABG	NA	NA	- Significant reduction in the primary outcome of IMV requirement in the first 5 days of life in the intervention group. Also, significant reduction in the incidence of PDA and IVH in the intervention group.
Olivier 2007 Canada	I: 24 C: 21	I: 34 C: 33 + 6	I: 2157 C: 2277	nCPAP	- FiO_2_ ≥ 35%	Bovine lung surfactant 100 mg/kg	I: LISA C: INSURE	- Respiratory acidosis confirmed by two pH values <7.20 along with pCO_2_ > 70 mmHg or—lack of improvement of oxygen requirement for 4 h following LISA	- FiO_2_ > 30%	I: 67 C: 52	- Significant reduction in the primary outcome of need for IMV or the occurrence of a pneumothorax requiring chest tube insertion in the first 3 days. Also, significant reduction in the episodes of desaturation in the intervention group.
OSIRIS collaborative group 1992 UK	I: 1344 C: 1346	I: 27.9 C: 27.9	I: 1121 C: 1122	IMV	- I: Prophylactic. - C: Late rescue: with a/AO_2_ < 0.22 on IMV	Exosurf 5 ml/kg	IMV in both groups	NA	- a/AO_2_ < 0.22	I: 23.3 C: 20.7	- Significant reduction in primary outcomes of death or dependence on supplemental oxygen at 28 days of age, death at any time and prolonged oxygen dependence. Also, significant reduction in the incidence of pneumothorax in the intervention group. No difference in other clinical outcomes.
Pareek 2021 India	I: 20 C: 20	I: 31.5 C: 31.4	I: 1500 C: 1460	nCPAP/NIPPV	Presence of ≥2 of the following: - Silverman Andersen Score ≥4 - FiO_2_ > 30% for <30 weeks and >40% for >30 weeks c. CXR suggestive of Grade II or III RDS and d. a/AO_2_ < 0.2	Bovine lung surfactant 100 mg/kg	I: LISA C: INSURE	- Silverman Andersen Score ≥7 - FiO_2_ requirement ≥60% on NRS - pH < 7.2 - pCO_2_ ≥ 60 mmHg or significant apnea	- FiO_2_ ≥ 40%	I: 90 C: 85	- No difference in the primary outcome of need for IMV within the first 72 h of life and secondary outcomes
Pinheiro 2016 USA	I: 30 C: 30	NA	I: 2118 C: 1945	nCPAP	- FiO_2_: 30–60%	Poractant 200 mg/kg	I: LISA C: INSURE	- Persistent apnea, severe retractions and/or inability to wean FiO_2_ below 60%	- FiO_2_ increases by 20% than the baseline within 8 h of therapy - FiO_2_ ≥ 60% or if ≥30% associated with worsening clinical signs of RDS after 8 h of first dose	I: 50 C: 53	- Significant reduction in the primary outcome of requirement of IMV. No significant difference in any other clinical outcomes.
Rodriguez-Fanjul 2020 Spain	I: 29 C: 29	I: 30 C: 30	I: 1500 C: 1520	nCPAP	- I: Lung USG - C: FiO_2_ > 30%	Poractant 200 mg/kg	LISA in both groups	- FiO_2_ > 50% despite surfactant therapy - Multiple episodes of apnea (>4 episodes per hour or >1 episode requiring PPV) or c. Respiratory acidosis (pCO_2_ > 65 mmHg) and pH < 7.20 in capillary samples	NA	I: 93.1 C: 88.9	Surfactant administration occurred earlier in the intervention group than in the control group. The intervention group had lower FiO_2_ requirement, and a higher SPO_2_ after surfactant administration. No difference in other outcomes.
Reininger 2005 USA	I: 52 C: 53	I: 32.6 C: 32.4	I: 1895 C: 1853	nCPAP	- FiO_2_ ≥ 30% initially and later changed to >21%	Bovine lung surfactant 100 mg/kg	I: INSURE C: IMV	- FiO_2_ > 30% and rising - PaCO_2_ > 50–55 torr - Apnea (>20 s), and/or moderate-to-severe retractions	- Need for IMV with FiO_2_ > 30% and PaO_2_ ≤ 80 torr at ≥6 h from last dose	I: 46 C: 57	Significant reduction in the primary outcome of requirement of IMV. Among secondary outcomes, there was significant reduction in requirement of subsequent doses of surfactant and FiO_2_.
Roberts 2017 USA	I: 50 C: 53	I: 32 C: 32	I: 1982 C: 1968	nCPAP/NIPPV/BiPAP	- Clinico-radiological evidence along with FiO_2_ 30–40% for ≥30 min	Poractant 200 mg/kg	I: LMA C: IMV	Requirement of any 2 of the following: - FiO_2_ > 40% for >30 min (to maintain SpO_2_ between 88% and 92%) - PCO_2_ > 65 mmHg on arterial or capillary blood gas or >70 on venous blood gas, or c. pH < 7.22 or 1 of the following: - Recurrent or severe apnea - Hemodynamic instability requiring Pressors - Repeat surfactant dose requirement or—clinician’s discretion	- Any baby requiring IMV	I: 68 C: 72	Significant reduction in the primary outcome of requirement of IMV in the first 7 days of life. Subgroup analysis showed that the reduction was more marked in the neonates between 28 and 31 weeks as compared to 32–35 weeks. The number of neonates requiring no supplemental oxygen and the mean oxygen requirement at 15 min, 30 min, and 1 h was significantly different, favouring the intervention group. No significant difference in other clinical outcomes.
Rojas 2009 USA	I: 141 C: 137	I: 29.3 C: 29.3	I: 1293 C: 1299	nCPAP	- Clinical assessment of respiratory distress	Bovine lung surfactant 100 mg/kg	I: INSURE C: IMV	- FiO_2_ > 75% for >30 min to maintain SpO_2_ within the preestablished target ranges - Persistent or recurrent desaturation <80% that did not respond to suctioning of the airways and PPV; c. PCO_2_ > 65 mm Hg and pH < 7.22 on an arterial or capillary blood gas analysis, in association with progressive respiratory failure	- Meeting the same treatment failure criteria	I: 88 C: 88	Significant reduction in primary outcome of requirement of IMV in the intervention arm. The reduction was mainly seen in the 30–31^+6^ weeks’ strata as compared to 27–29 weeks’. Among secondary outcomes, there was significant reduction in requirement of subsequent doses of surfactant and air leaks in the intervention arm. A significant reduction in the incidence of CLD was also noted in 30–31^+6^ weeks strata in the intervention arm.
Sabzehei 2022 Iran	I: 56 C: 56	I: 29.6 C: 30.6	I: 1530 C: 1677	nCPAP	- FiO_2_ > 40%	Poractant 200 mg/kg	I: LISA C: INSURE	- Respiratory acidosis (pH < 7.2 with PaCO_2_ > 60–65 mmHg) - Hypoxia (PaO_2_ < 50 mmHg despite receiving oxygen or FiO_2_ > 40% under nCPAP), and—severe apnea.	- FiO_2_ > 40%	I: 55.9 C: 67.9	No significant difference in the primary outcome of requirement of IMV in the first 72 h of life or secondary outcomes
Sadeghnia 2014 Iran	I: 35 C: 35	I: 35 C: 35	I: 2352 C: 2372	nCPAP	- FiO_2_ ≥ 30%	Bovine lung surfactant 100 mg/kg	I: LMA C: INSURE	- Need for FiO_2_ ≥ 70% to maintain oxygen saturation from 89 to 95% - Apnea more than thrice, which needed stimulation and bag and mask ventilation - Inability to maintain the acceptable ventilation and respiratory failure, which was identified by pH < 7.2 and PaCO_2_ > 65 mmHg	- FiO_2_ > 50%	I: 51.4 C: 65.7	Significant improvement in average a/AO_2_ after surfactant administration
SUPPORT 2010 USA	I: 653 C: 663	I: 26.2 C: 26.2	I: 826 C: 835	nCPAP	- I: Prophylactic - C: FiO_2_ > 50%		IMV in both groups	- FiO_2_ > 0.50 to maintain SpO_2_ ≥ 88% for 1 h - PaCO_2_ > 65 mm Hg, within 1 h before intubation; c. Hemodynamic instability, defined as BP low for GA, poor perfusion, or both, requiring volume or pressor support for a period of ≥4 h	NA	I: 95.6 C: 96.8	No difference in the primary outcome of composite of death or physiological BPD assessed at 36 weeks’ PMA. However, there was significant reduction in the duration as well as requirement of IMV, requirement of postnatal steroid for BPD. Posthoc analysis showed significant reduction in mortality in neonates between 24 and 25^+6^ weeks both during hospitalisation as well as at 36 weeks’ PMA but not in neonates between 26 and 27^+6^ weeks’.
Tapia 2012 South America Multi-centre	I: 131 C: 125	I: 29.8 C: 29.5	I: 1196 C: 1197	I: CPAP C: Oxyhood/low flow oxygen cannula	- I: CPAP followed by INSURE if FiO_2_ > 35% - C: Infants with signs of RDS were started on oxyhood/low flow oxygen cannula and given surfactant followed by continued IMV if FiO_2_ > 35%	Bovine lung surfactant 100 mg/kg	I: INSURE C: IMV	- FiO_2_ ≥ 60% for at least 2 h after surfactant administration - Need for a third dose of surfactant - More than 3 episodes of apnea and bradycardia (heart rate <80/min) per hour; and—PaCO_2_ > 60 mm Hg with pH < 7.20 on arterial blood gas analyses within 30 min	- FiO_2_ > 40%	I: 90.8 C: 88	The need for IMV was lower in the CPAP/INSURE group (29.8% vs. 50.4%; p = 0.001), as well as the use of surfactant (27.5% vs. 46.4%; p = 0.002). There were no differences in the other common neonatal morbidities.
TEXAS group 2004 USA	I: 65 C: 67	I: 32.8 C: 32.7	I: 2040 C: 2068	nCPAP	- FiO_2_ ≥ 40%	Bovine lung surfactant 100 mg/kg	I: INSURE C: IMV	Clinician’s discretion	NA	I: 43.2 C: 32.3	Multicentric study. Significant reduction in duration of IMV, no difference in other outcomes.
Vaucher 1993 USA	I: 25 C: 25	I: 27.5 C: 28	I: 1051 C: 1117	IMV	- I: Prophylactic - C: Late rescue with FiO_2_ ≥ 50% and MAP ≥ 7 cm H_2_O	Human surfactant three 1.0–1.5 ml doses	IMV in both groups	NA	- FiO_2_ ≥ 50% or MAP ≥ 2 cm H_2_O above the lowest level achieved after initial treatment	NA	Significantly higher MDI and PDI score in rescue as compared to prophylactic group.
Verder 2013 Sweden	I: 192 C: 188	I: 28.1 C: 28.3	I: 1073 C: 1070	nCPAP	- I: Lamellar body count <8000/μl - C: a/AO_2_ < 0.36	Poractant 200 mg/kg	INSURE in both groups	- a/AO_2_ < 0.15 for >30 min - >4 episodes of apnea per hour with need for stimulation, or that requiring mask ventilation >2 episodes/hour	- a/AO_2_ < 0.36	I: 94 C: 98	Significantly higher a/AO_2_ at 6 h overall as well as in neonates between 26 and 29 weeks’ in the intervention group. Duration of supplemental oxygen was lower in neonates between 26 and 29 weeks’. Supplemental oxygen at 28 days was significantly lower in the intervention group.
Vento 2020 Italy	I: 107 C: 111	I: 26.4 C: 26.3	I: 815 C: 788	nCPAP	- FiO_2_ ≥ 30%	Poractant 200 mg/kg	I: INRECSURE C: INSURE	- FiO_2_ > 40% - pCO_2_ > 65 mm Hg and pH < 7·20 - Apnea (>4 episodes per hour or >2 episodes per hour requiring ventilation with bag and mask)	- CPAP failure criteria, 24 h after 1st dose	I: 93 C: 90	Significant reduction in the primary outcome of requirement of IMV in the first 72 h of life. The FiO_2_ requirement during surfactant administration was lower in the intervention arm. Per-protocol analysis suggested a protective effect of the recruitment procedure on the outcome of mortality.
Verder 1994 Sweden	I: 35 C: 33	I: 30 C: 29	I: 1380 C: 1303	nCPAP	- a/AO_2_ < 0.22	Poractant 200 mg/kg	I: INSURE C: IMV	- a/AO_2_ < 0.15 for at least 10 min - Severe apnea	- FiO_2_ > 60%	I: 40 C: 61	Significant reduction in the primary outcome of IMV requirement in the INSURE group. The reduction was more in male neonates. Significant improvement in aAO_2_ 6 h after randomisation.
Verder 1999 Sweden	I: 33 C: 27	I: 27 C: 28	I: 950 C: 935	nCPAP	- I: a/AO_2_ 0.35 to 0.22 - C: a/AO_2_ 0.21 to 0.15	Poractant 200 mg/kg	INSURE in both groups	- a/AO_2_ < 0.15, decreasing further over a period of >30 min - Severe apnea defined as >4 episodes per hour or need of mask ventilation >2 times per hour, or—inability of extubation within 1 h after intubation for surfactant treatment	NA	I: 79 C: 81	Significant reduction in the primary outcome composite of requirement of IMV or death within the first 7 days of life. In the early treatment arm. There was also a significant reduction in the primary outcome parameters at discharge, significant reduction in IMV before discharge, aAO_2_ after 6 h, PVL and PDA in the early treatment arm. No significant difference in other secondary outcomes across both groups.
Walti 1995 France	I: 134 C: 122	I: 28.9 C: 28.3	I: 1211 C: 1150	IMV	- I: Prophylactic - C: Late rescue: Clinico-radiological evidence and a PaO_2_/FiO_2_ ratio <150 (mmHg) at MAP of 8 cm H_2_O	Poractant 100 mg/kg	IMV in both groups	NA	- PaO_2_/FiO_2_ ratio <150 (mmHg) at an MAP of 8 cm H_2_O	I: 17 C: 11	No significant difference in the primary outcome of survival without BPD at 28 days after birth. However, significant reduction in requirement of IMV, number of doses of surfactant and improved parameters of gas exchange in the prophylactic group.
Yang 2022 China	I: 92 C: 92	I: 29.7 C: 29.7	I: 1320 C: 1290	nCPAP	- As rescue measure in both the groups when there was CPAP failure. Criteria used to define CPAP failure was same as that for endotracheal intubation	Poractant 200 mg/kg	I: INRECSURE C: INSURE	- FiO_2_ ≥ 40% to maintain SpO_2_ at 88–94% for at least 30 min - Arterial blood gas analysis revealed respiratory acidosis: pCO_2_ > 65 mmHg (8.5 kPa), pH < 7.20 - More than 4 episodes of apnea per hour, or more than 2 severe episodes defined as apnea requiring nasal PPV	- Same as that for IMV	I: 59 C: 52	INRECSURE group required lesser IMV and repeat surfactant doses and lesser duration of IMV when compared to INSURE group. There were no significant differences in the other complications between the two groups
Yang 2019 China	I: 47 C: 50	I: 33.7 C: 34.1	I: 2106 C: 2219	nCPAP	- FiO_2_ > 40%	Poractant 200 mg/kg	I: LISA C: INSURE	- FiO_2_ ≥ 60% - pH < 7.20 and/or PaCO_2_ > 65 mm Hg - Severe apnea	- If requiring IMV after the first dose	I: 27.7 C: 22	Significantly higher SPO_2_ in the LISA group till 6 min after the procedure. The SBP and DBP was significantly higher in the INSURE group. No difference in other clinical outcomes.

a/AO_2_, arterial alveolar oxygen tension ratio; ACORN, acute care of at-risk newborns; ANS, antenatal corticosteroids; BW, birth weight; BPD, bronchopulmonary dysplasia; BiPAP, biphasic CPAP; CA, corrected age; C, control; I, intervention; CXR, chest radiography; DBP, diastolic blood pressure; IVH, intraventricular hemorrhage; IMV, invasive mechanical ventilation; ET, endotracheal tube; FiO_2_, fraction of inspired oxygen; g, grams; GA, gestational age; INSURE, Intubate-Surfactant-Extubate; INRECSURE, Intubate-Recruit-Surfactant-Extubate; LMA, laryngeal mask airway; LISA, less invasive surfactant administration; MAP, mean airway pressure; MABP, mean arterial blood pressure; MDI, mental developmental index; nCPAP, nasal continuous positive airway pressure; NEC, necrotizing enterocolitis; NIPPV, non-invasive positive pressure ventilation; NA, not available; NRS, non-invasive respiratory support; pCO_2_/PaCO_2_, partial pressure of carbon dioxide in the blood; PaO_2_, partial pressure of oxygen in the blood; PAO_2_, partial pressure of alveolar oxygen; PDA, patent ductus arteriosus; PDI, psychomotor developmental index; PIP, peak inspiratory pressure; PEEP, post end expiratory pressure; PMA, post menstrual age; PIE, pulmonary interstitial emphysema; PPV, positive pressure ventilation; RDS, respiratory distress syndrome; rScO_2_, cerebral oxygen saturation; SpO_2_, oxygen saturation as measured by pulse oximetry; SBP, systolic blood pressure; ROP, retinopathy of prematurity; tcPO_2_, transcutaneously monitored PaO_2_; USG, ultrasonography; w, weeks.

### Risk of bias assessment

Whilst 23 studies had a low risk of bias,^11^^,^^23^^,^^24^^,^^28^^,^^32^^,^^33^^,^^37^^,^^46^^,^^48^^,^^51^^,^^54^^,^^57^, ^58^, ^59^^,^^61^^,^^62^^,^^65^, ^66^, ^67^^,^^70^^,^^73^^,^^75^^,^^76^ 21 had some concerns^8^^,^^10^^,^^22^^,^^27^^,^^29^, ^30^, ^31^^,^^34^^,^^38^^,^^42^^,^^44^^,^^45^^,^^49^^,^^52^^,^^55^^,^^60^^,^^64^^,^^68^^,^^69^^,^^72^^,^^74^ and 13 had a high risk of overall bias.^25^^,^^26^^,^^35^^,^^36^^,^^39^, ^40^, ^41^^,^^43^^,^^47^^,^^50^^,^^56^^,^^63^^,^^71^ The risk of bias of one study could not be assessed as only an abstract was available.^53^ Most of the studies with a high risk of bias had ‘some concerns’ in randomization and selection of reported results. The predominant reason for RCTs which had ‘some concerns’ was due to unavailability of registered protocols (eTable S2).

### Outcomes

#### Primary outcome: requirement of IMV

##### Neonates ≤30 weeks

**Fig. 2:**
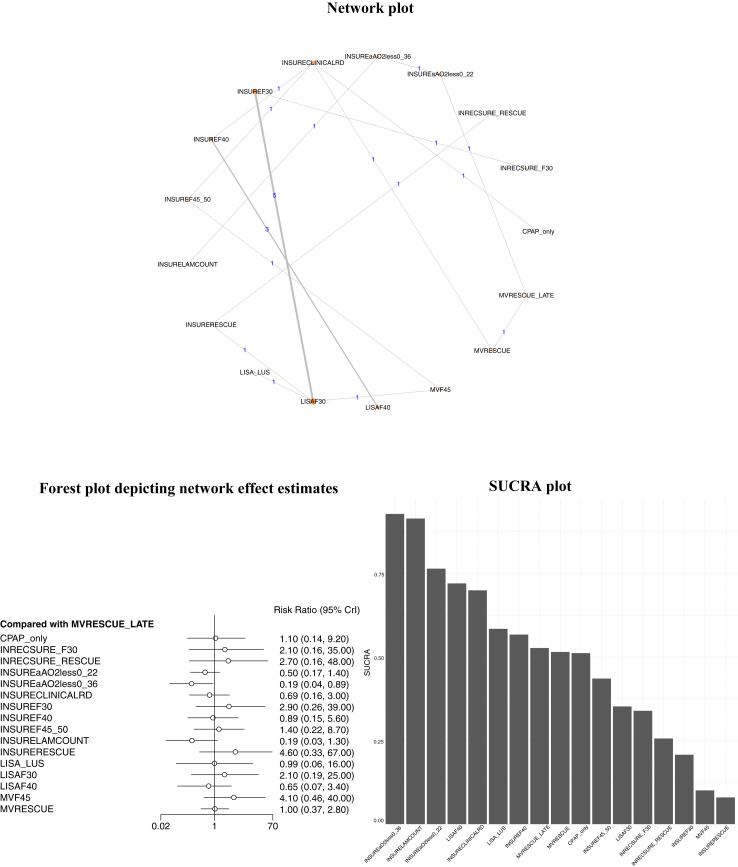
Network plot, Forest plot depicting the network effect estimates and SUCRA plot for the primary outcome of requirement of Invasive Mechanical Ventilation in preterm neonates ≤30 weeks' gestation.

22 studies with 17 interventions including 3531 neonates with 1515 events were evaluated in the NMA (eTable S3). The network plot is illustrated in Fig. 2. Very low CoE suggested that Intubate-Surfactant-Extubate (INSURE) using a threshold of arterial alveolar oxygen tension ratio of <0.36 (INSUREaAO_2_less0_36) was possibly associated with lesser risk of IMV when compared to the interventions: INSURE as a rescue modality (INSURERESCUE) [RR 95% CrI: 0.04 (0.00–0.85)], surfactant administration at an FiO_2_ of 45% followed by mechanical ventilation (MVF45) [0.05 (0.00–0.68)] and surfactant administration as a late rescue measure while on mechanical ventilation (MVRESCUE_LATE) [0.19 (0.04–0.89)]. INSURE based on clinical respiratory distress assessment (INSURECLINICALRD) [0.17 (0.03–0.88)] and centrifuged gastric lavage lamellar body counts (INSURELAMCOUNT) [0.05 (0.00–0.82)] also possibly resulted in lesser risk of IMV when compared to MVF45, CoE: very low. Low CoE suggested that the strategy Intubate-Recruit-Surfactant-Extubate (INRECSURE) either at an FiO_2_ of 30% (INRECSUREF30) or as rescue possibly might not reduce the risk of IMV when compared to INSURE at similar thresholds. Clinical benefit or harm could not be ruled out for FiO_2_ thresholds of 30% vs. 40% irrespective of the modality utilized for surfactant administration [e.g., Less Invasive Surfactant Administration (LISA) or INSURE] since the CoE was very low and effect estimates were not statistically significant. Similarly, clinical benefit or harm could not be ruled out for LISA based on lung ultrasound (LISA_LUS) when compared to LISA at FiO_2_ of 30% (LISAF30) or 40% (LISAF40) and INSURE at FiO_2_ of 30% (INSUREF30) or 40% (INSUREF40), CoE: very low to low. There were no statistically significant differences between the other interventions evaluated. SUCRA ranked the following strategies as the three most efficacious: INSUREaAO_2_less0_36 (SUCRA: 0.93), INSURELAMCOUNT (SUCRA: 0.91) and INSUREaAO_2_less0_22 (SUCRA: 0.76). The SUCRA plot and the forest plot of the NMA effect estimates are illustrated in Fig. 2. The matrix plot depicting the NMA effect estimates for all the comparisons is given in Table 2. The CoE for NMA effect estimates for all the comparisons is given in Table 3. The forest plot of pair-wise comparisons of RCTs is provided in eFigure S1. Inconsistency assessment for this subgroup was not possible due to the sparseness of the network. When Inconsistency was assessed for neonates of all gestations, it was not detected (eFigure S2).

**Table 2:** Matrix plot depicting the network effect estimates for the various comparisons for the primary outcome of requirement of invasive mechanical ventilation in preterm neonates of ≤30 weeks’ gestation.

CPAP_only	1.99 (0.13, 32.61)	2.53 (0.14, 45.05)	0.46 (0.04, 4.69)	0.17 (0.01, 2.31)	0.65 (0.13, 3)	2.69 (0.22, 36.53)	0.83 (0.12, 5.46)	1.29 (0.18, 8.61)	0.18 (0.01, 2.84)	4.28 (0.29, 62.75)	0.92 (0.05, 15.59)	1.93 (0.16, 23.55)	0.59 (0.06, 3.72)	3.83 (0.38, 38.62)	0.96 (0.14, 5.88)	0.93 (0.11, 7.19)
0.5 (0.03, 7.51)	INRECSUREF30	1.29 (0.18, 7.93)	0.23 (0.01, 3.88)	0.09 (0, 1.81)	0.33 (0.03, 2.91)	1.35 (0.48, 3.78)	0.42 (0.03, 4.88)	0.66 (0.08, 4.29)	0.09 (0, 2.16)	2.17 (0.42, 9.6)	0.47 (0.07, 2.47)	0.98 (0.28, 2.91)	0.3 (0.01, 2.98)	1.95 (0.38, 8.37)	0.48 (0.03, 5.38)	0.47 (0.03, 6.38)
0.4 (0.02, 7.07)	0.78 (0.13, 5.66)	INRECSURE_RESCUE	0.18 (0.01, 3.75)	0.07 (0, 1.75)	0.26 (0.02, 2.91)	1.05 (0.23, 5.62)	0.33 (0.02, 4.77)	0.51 (0.06, 4.41)	0.07 (0, 2.05)	1.68 (0.58, 4.93)	0.37 (0.05, 2.6)	0.76 (0.17, 3.39)	0.24 (0.01, 2.88)	1.51 (0.25, 9.08)	0.38 (0.03, 5.27)	0.37 (0.02, 6.28)
2.16 (0.21, 23.56)	4.27 (0.26, 86.51)	5.49 (0.27, 119.9)	INSUREaAO_2_less0_22	0.38 (0.11, 1.17)	1.39 (0.23, 8.52)	5.76 (0.42, 97.49)	1.79 (0.22, 14.85)	2.77 (0.33, 23.94)	0.39 (0.08, 1.8)	9.22 (0.55, 164.96)	2 (0.1, 39.89)	4.16 (0.3, 61.55)	1.3 (0.1, 8.98)	8.25 (0.72, 100.51)	2.05 (0.47, 8.97)	1.99 (0.69, 5.8)
5.78 (0.43, 82.85)	11.39 (0.55, 289.55)	14.63 (0.57, 397.65)	**2.63 (0.85, 8.84)**	INSUREaAO_2_less0_36	3.69 (0.44, 31.98)	15.35 (0.88, 332.28)	4.76 (0.43, 53.73)	7.4 (0.66, 84.39)	1.03 (0.37, 2.87)	24.6 (1.17, 559.62)	5.31 (0.22, 131.63)	11.1 (0.62, 210.47)	3.45 (0.21, 32.65)	22.02 (1.47, 351.19)	5.46 (0.85, 36.11)	5.31 (1.12, 26.41)
1.54 (0.33, 7.98)	3.05 (0.34, 34.07)	3.92 (0.34, 46.76)	0.72 (0.12, 4.36)	0.27 (0.03, 2.29)	INSURECLINICALRD	4.11 (0.59, 36.17)	1.28 (0.43, 3.93)	1.98 (0.65, 6.27)	0.28 (0.03, 2.97)	6.58 (0.75, 62.84)	1.43 (0.13, 15.63)	2.97 (0.43, 22.47)	0.93 (0.18, 2.71)	5.87 (1.13, 34.74)	1.48 (0.52, 4.18)	1.44 (0.34, 6.12)
0.37 (0.03, 4.61)	0.74 (0.26, 2.08)	0.95 (0.18, 4.38)	0.17 (0.01, 2.39)	0.07 (0, 1.13)	0.24 (0.03, 1.69)	INSUREF30	0.31 (0.03, 2.9)	0.49 (0.08, 2.37)	0.07 (0, 1.36)	1.61 (0.45, 4.83)	0.35 (0.07, 1.35)	0.73 (0.38, 1.18)	0.22 (0.01, 1.8)	1.45 (0.42, 4.24)	0.36 (0.03, 3.24)	0.35 (0.03, 3.88)
1.2 (0.18, 8.61)	2.37 (0.2, 33.82)	3.05 (0.21, 46.63)	0.56 (0.07, 4.59)	0.21 (0.02, 2.32)	0.78 (0.25, 2.34)	3.2 (0.34, 36.93)	INSUREF40	1.55 (0.32, 7.58)	0.22 (0.02, 2.95)	5.13 (0.44, 63.04)	1.11 (0.08, 15.32)	2.31 (0.25, 23.06)	0.73 (0.26, 1.16)	4.58 (0.63, 36.76)	1.15 (0.25, 5.23)	1.12 (0.18, 6.88)
0.78 (0.12, 5.64)	1.52 (0.23, 12.56)	1.96 (0.23, 17.98)	0.36 (0.04, 3)	0.14 (0.01, 1.51)	0.5 (0.16, 1.54)	2.05 (0.42, 12.93)	0.65 (0.13, 3.14)	INSUREF45_50	0.14 (0.01, 1.88)	3.28 (0.51, 23.32)	0.72 (0.09, 5.88)	1.48 (0.31, 7.98)	0.47 (0.06, 2.04)	2.92 (0.88, 11.68)	0.75 (0.16, 3.43)	0.72 (0.11, 4.47)
5.64 (0.35, 96.61)	11.1 (0.46, 332.74)	14.28 (0.49, 445.34)	2.58 (0.56, 12.46)	0.97 (0.35, 2.74)	3.61 (0.34, 39.3)	14.96 (0.73, 381.97)	4.65 (0.34, 63.77)	7.2 (0.53, 101.6)	INSURELAMCOUNT	23.98 (0.98, 632.23)	5.18 (0.18, 149.34)	10.83 (0.52, 243.13)	3.37 (0.17, 38.14)	21.44 (1.22, 409.75)	5.33 (0.64, 45.08)	5.18 (0.8, 34.15)
0.23 (0.02, 3.48)	0.46 (0.1, 2.4)	0.59 (0.2, 1.72)	0.11 (0.01, 1.83)	**0.04 (0, 0.85)**	0.15 (0.02, 1.34)	0.62 (0.21, 2.21)	0.19 (0.02, 2.25)	0.3 (0.04, 1.97)	0.04 (0, 1.02)	INSURERESCUE	0.22 (0.04, 1.12)	0.45 (0.16, 1.27)	0.14 (0.01, 1.37)	0.9 (0.21, 3.8)	0.22 (0.02, 2.52)	0.22 (0.02, 2.99)
1.08 (0.06, 19.08)	2.13 (0.4, 14.05)	2.73 (0.39, 20.94)	0.5 (0.03, 9.7)	0.19 (0.01, 4.55)	0.7 (0.06, 7.55)	2.88 (0.74, 14)	0.9 (0.07, 12.49)	1.4 (0.17, 11.38)	0.19 (0.01, 5.43)	4.59 (0.89, 26.26)	LISA_LUS	2.07 (0.57, 8.67)	0.64 (0.03, 7.77)	4.11 (0.82, 23.12)	1.03 (0.08, 13.8)	1.01 (0.06, 16.11)
0.52 (0.04, 6.41)	1.02 (0.34, 3.6)	1.32 (0.29, 5.86)	0.24 (0.02, 3.36)	0.09 (0, 1.6)	0.34 (0.04, 2.31)	1.37 (0.85, 2.63)	0.43 (0.04, 4.01)	0.68 (0.13, 3.22)	0.09 (0, 1.92)	2.21 (0.79, 6.21)	0.48 (0.12, 1.77)	LISAF30	0.31 (0.02, 2.48)	1.98 (0.73, 5.46)	0.5 (0.05, 4.45)	0.48 (0.04, 5.39)
1.7 (0.27, 16.9)	3.3 (0.34, 68.63)	4.24 (0.35, 94.77)	0.77 (0.11, 9.75)	0.29 (0.03, 4.8)	1.08 (0.37, 5.44)	4.45 (0.55, 76.01)	1.38 (0.86, 3.92)	2.15 (0.49, 16.44)	0.3 (0.03, 6.06)	7.12 (0.73, 130.78)	1.55 (0.13, 30.6)	3.22 (0.4, 47.16)	LISAF40	6.4 (0.98, 74.38)	1.59 (0.39, 11.52)	1.54 (0.29, 14.96)
0.26 (0.03, 2.66)	0.51 (0.12, 2.62)	0.66 (0.11, 3.99)	0.12 (0.01, 1.39)	**0.05 (0, 0.68)**	**0.17 (0.03, 0.88)**	0.69 (0.24, 2.39)	0.22 (0.03, 1.6)	0.34 (0.09, 1.14)	**0.05 (0, 0.82)**	1.11 (0.26, 4.75)	0.24 (0.04, 1.22)	0.5 (0.18, 1.38)	0.16 (0.01, 1.02)	MVF45	0.25 (0.03, 1.76)	0.24 (0.03, 2.17)
1.05 (0.17, 7.18)	2.07 (0.19, 28.69)	2.65 (0.19, 39.47)	0.49 (0.11, 2.11)	0.18 (0.03, 1.18)	0.68 (0.24, 1.91)	2.78 (0.31, 31.02)	0.87 (0.19, 3.98)	1.34 (0.29, 6.34)	0.19 (0.02, 1.57)	4.45 (0.4, 54.01)	0.97 (0.07, 13.14)	2.01 (0.22, 19.52)	0.63 (0.09, 2.55)	3.97 (0.57, 31.11)	MVRESCUE	0.97 (0.35, 2.68)
1.08 (0.14, 9.22)	2.13 (0.16, 35.41)	2.72 (0.16, 48.31)	0.5 (0.17, 1.44)	**0.19 (0.04, 0.89)**	0.69 (0.16, 2.97)	2.86 (0.26, 39.07)	0.89 (0.15, 5.58)	1.38 (0.22, 8.72)	0.19 (0.03, 1.26)	4.58 (0.33, 66.63)	0.99 (0.06, 16.16)	2.07 (0.19, 24.59)	0.65 (0.07, 3.42)	4.09 (0.46, 39.55)	1.03 (0.37, 2.84)	MVRESCUE_LATE

CPAP_only, continuous positive airway pressure without any surfactant administration; ‘F’ followed by a number, FiO_2_ threshold expressed as percentage; INSURE, Intubate-Surfactant-Extubate; INSUREaAO_2_less_0_36, INSURE at arterial alveolar tension ratio of less than 0.36; INSUREaAO_2_less_0_22, INSURE at arterial alveolar tension ratio of less than 0.22; INRECSURE, Intubate-Recruit-Surfactant-Extubate; INSURECLINICALRD, INSURE based on clinical respiratory distress; INSURELAMCOUNT, INSURE based on centrifuged gastric lavage lamellar body counts; LISA, less invasive surfactant administration; LISACLINICALRD, LISA based clinical respiratory distress; LISALUS, LISA guided by lung ultrasound; MVRESCUE, surfactant administration when respiratory failure is diagnosed followed by continued mechanical ventilation; MVRESCUE_LATE, surfactant administration on mechanical ventilation when a particular mean airway pressure or FiO_2_ threshold is reached; MVF, surfactant administration when a particular FiO_2_ threshold is reached followed by continued mechanical ventilation. The effect estimates expressed in bold fonts were statistically significant with the 95% CI not crossing the line of no effect.

**Table 3:** Certainty of evidence for the various comparisons for the primary outcome of requirement of invasive mechanical ventilation in preterm neonates of ≤30 weeks’ gestation.

Comparison	CoE for direct evidence	CoE for indirect evidence	CoE for NMA estimate
CPAP_only:INSURECLINICALRD	Very low	–	Very low
INRECSUREF30:INSUREF30	Low		Low
INRECSURERESCUE:INSURERESCUE	Low	–	Low
INSUREaAO_2_less_0_22:INSUREaAO_2_less_0_36	Very low	–	**Very low**
INSUREaAO_2_less_0_22:MVRESCUE_LATE	Low	–	Low
INSUREaAO_2_less_0_36:INSURELAMCOUNT	Low	–	Low
INSURECLINICALRD:INSUREF45-50	Moderate	–	Moderate
INSUREF30:LISAF30	High	–	High
INSUREF40:LISAF40	Moderate	–	Moderate
INSUREF45_50:MVF45	Very low	–	Very low
INSURERESCUE:LISAF30	Moderate	–	Moderate
LISAF30:LISALUS	Low	–	Low
LISAF30:MVF45	High	–	High
MVRESCUE:MVRESCUE_LATE	Low	–	Low
INSURECLINICALRD:MVRESCUE	Moderate	–	Moderate
INSUREF40:MVRESCUE	–	Low	Low
INSURECLINICALRD:INSUREF40	Low	–	Low
CPAP_only:INRECSUREF30	–	Very low	Very low
CPAP_only:INRECSURERESCUE	–	Very low	Very low
CPAP_only:INSUREaAO_2_less_0_22	–	Very low	Very low
CPAP_only:INSUREaAO_2_less_0_36	–	Very low	Very low
CPAP_only:INSUREF30	–	Very low	Very low
CPAP_only:INSUREF40	–	Very low	Very low
CPAP_only:INSUREF45	–	Very low	Very low
CPAP_only:INSUREF50	–	Very low	Very low
CPAP_only:INSURELAMCOUNT	–	Very low	Very low
CPAP_only:INSURERESCUE	–	Very low	Very low
CPAP_only:LISAF30	–	Very low	Very low
CPAP_only:LISAF40	–	Very low	Very low
CPAP_only:LISALUS	–	Very low	Very low
CPAP_only:MVF45	–	Very low	Very low
CPAP_only:MVRESCUE	–	Very low	Very low
CPAP_only:MVRESCUE_LATE	–	Very low	Very low
INRECSUREF30:INRECSURERESCUE	–	Low	Low
INRECSUREF30:INSUREaAO_2_less_0_22	–	Very low	Very low
INRECSUREF30:INSUREaAO_2_less_0_36	–	Very low	Very low
INRECSUREF30:INSURECLINICALRD	–	Very low	Very low
INRECSUREF30:INSUREF40	–	Very low	Very low
INRECSUREF30:INSUREF45	–	Very low	Very low
INRECSUREF30:INSURELAMCOUNT	–	Very low	Very low
INRECSUREF30:INSURERESCUE	–	Very low	Very low
INRECSUREF30:LISAF30	–	Low	Low
INRECSUREF30:LISAF40	–	Low	Low
INRECSUREF30:LISALUS	–	Low	Low
INRECSUREF30:MVF45	–	Low	Low
INRECSUREF30:MVRESCUE	–	Very low	Very low
INRECSUREF30:MVRESCUE_LATE	–	Very low	Very low
INRECSUREF30:NEBaAO_2_less_0_22	–	Very low	Very low
INRECSURERESCUE:INSUREaAO_2_less_0_22	–	Very low	Very low
INRECSURERESCUE:INSUREaAO_2_less_0_36	–	Very low	Very low
INRECSURERESCUE:INSURECLINICALRD	–	Very low	Very low
INRECSURERESCUE:INSUREF30	–	Very low	Very low
INRECSURERESCUE:INSUREF40		Very low	Very low
INRECSURERESCUE:INSUREF45	–	Very low	Very low
INRECSURERESCUE:INSURELAMCOUNT	–	Very low	Very low
INRECSURERESCUE:LISAF30	–	Moderate	Moderate
INRECSURERESCUE:LISAF40	–	Very low	Very low
INRECSURERESCUE:LISALUS	–	Low	Low
INRECSURERESCUE:MVF45	–	Very low	Very low
INRECSURERESCUE:MVRESCUE		Very low	Very low
INRECSURERESCUE:MVRESCUE_LATE	–	Very low	Very low
INSUREaAO_2_less_0_22:INSURECLINICALRD	–	Low	Low
INSUREaAO_2_less_0_22:INSUREF30	–	Very low	Very low
INSUREaAO_2_less_0_22:INSUREF40	–	Low	Low
INSUREaAO_2_less_0_22:INSUREF45_50	–	Very low	Very low
INSUREaAO_2_less_0_22:INSURELAMCOUNT	–	Very low	Very low
INSUREaAO_2_less_0_22:INSURERESCUE	–	Very low	Very low
INSUREaAO_2_less_0_22:LISAF30	–	Very low	Very low
INSUREaAO_2_less_0_22:LISAF40	–	Very low	Very low
INSUREaAO_2_less_0_22:LISALUS	–	Very low	Very low
INSUREaAO_2_less_0_22:MVF45	–	Very low	Very low
INSUREaAO_2_less_0_22:MVRESCUE	Low	Very low	Low
INSUREaAO_2_less_0_22:NEBF22_30	–	Very low	Very low
INSUREaAO_2_less_0_36:INSURECLINICALRD	–	Low	Low
INSUREaAO_2_less_0_36:INSUREF30	–	Very low	Very low
INSUREaAO_2_less_0_36:INSUREF40	–	Very low	Very low
INSUREaAO_2_less_0_36:INSUREF45_50	–	Very low	Very low
INSUREaAO_2_less_0_36:INSURERESCUE	–	Very low	**Very low**
INSUREaAO_2_less_0_36:LISAF30	–	Very low	Very low
INSUREaAO_2_less_0_36:LISAF40	–	Very low	Very low
INSUREaAO_2_less_0_36:LISALUS	–	Very low	Very low
INSUREaAO_2_less_0_36:MVF45	–	Very low	**Very low**
INSUREaAO_2_less_0_36:MVRESCUE	–	Very low	Very low
INSUREaAO_2_less_0_36:MVRESCUE_LATE	–	Very low	**Very low**
INSURECLINICALRD:INSUREF30	–	Very low	Very low
INSURECLINICALRD:INSUREF45 _50	–	Very low	Very low
INSURECLINICALRD:INSURELAMCOUNT	–	Very low	Very low
INSURECLINICALRD:INSURERESCUE	–	Very low	Very low
INSURECLINICALRD:LISAF30	–	Very low	Very low
INSURECLINICALRD:LISAF40	–	Very low	Very low
INSURECLINICALRD:LISALUS	–	Very low	Very low
INSURECLINICALRD:MVF45	–	Very low	**Very low**
INSURECLINICALRD:MVRESCUE_LATE	–	Low	Low
INSURECLINICALRD:NEBaAO_2_less_0_22	–	Low	Low
INSUREF30:INSUREF40	–	Very low	Very low
INSUREF30:INSUREF45_50	–	Very low	Very low
INSUREF30:INSURELAMCOUNT	–	Very low	Very low
INSUREF30:INSURERESCUE	–	Moderate	Moderate
INSUREF30:LISAF40	–	Very low	Very low
INSUREF30:LISALUS	–	Low	Low
INSUREF30:MVF45	–	Very low	Very low
INSUREF30:MVRESCUE	–	Low	Low
INSUREF30:MVRESCUE_LATE	–	Low	Low
INSUREF40:INSUREF45_50	–	Low	Low
INSUREF40:INSURELAMCOUNT	–	Very low	Very low
INSUREF40:INSURERESCUE	–	Very low	Very low
INSUREF40:LISAF30	–	Very low	Very low
INSUREF40:LISALUS	–	Very low	Very low
INSUREF40:MVF45	–	Very low	Very low
INSUREF40:MVRESCUE_LATE	–	Low	Low
INSUREF45_50:INSURELAMCOUNT	–	Very low	Very low
INSUREF45_50:INSURERESCUE	–	Very low	Very low
INSUREF45_50:LISAF30	–	Very low	Very low
INSUREF45_50:LISAF40	–	Very low	Very low
INSUREF45_50:LISALUS	–	Very low	Very low
INSUREF45_50:MVRESCUE	–	Moderate	Moderate
INSUREF45_50:MVRESCUE_LATE	–	Moderate	Moderate
INSURELAMCOUNT:INSURERESCUE	–	Very low	Very low
INSURELAMCOUNT:LISAF30	–	Very low	Very low
INSURELAMCOUNT:LISAF40	–	Very low	Very low
INSURELAMCOUNT:LISALUS	–	Very low	Very low
INSURELAMCOUNT:MVF45	–	Very low	**Very low**
INSURELAMCOUNT:MVRESCUE	–	Very low	Very low
INSURELAMCOUNT:MVRESCUE_LATE	–	Very low	Very low
INSURERESCUE:LISAF40	–	Very low	Very low
INSURERESCUE:LISAF30	Moderate	–	Moderate
INSURERESCUE:LISALUS	–	Very low	Very low
INSURERESCUE:MVF45	–	Moderate	Moderate
INSURERESCUE:MVRESCUE	–	Very low	Very low
INSURERESCUE:MVRESCUE_LATE	–	Very low	Very low
LISAF30:LISAF40		Very low	Very low
LISAF30:MVRESCUE	–	Very low	Very low
LISAF30:MVRESCUE_LATE	–	Very low	Very low
LISAF40:LISALUS	–	Very low	Very low
LISAF40:MVF45	–	Very low	Very low
LISAF40:MVRESCUE	–	Low	Low
LISAF40:MVRESCUE_LATE	–	Low	Low
LISALUS:MVF45	–	Very low	Very low
LISALUS:MVRESCUE	–	Very low	Very low
LISALUS:MVRESCUE_LATE	–	Very low	Very low
MVF45:MVRESCUE	–	Very low	Very low
MVF45:MVRESCUE_LATE	–	Very low	Very low

CoE, certainty of evidence; CPAP_only, continuous positive airway pressure without any surfactant administration; ‘F’ followed by a number, FiO_2_ threshold expressed as percentage; INSURE, Intubate-Surfactant-Extubate; INSUREaAO_2_less_0_36, INSURE at arterial alveolar tension ratio of less than 0.36; INSUREaAO_2_less_0_22, INSURE at arterial alveolar tension ratio of less than 0.22; INRECSURE, Intubate-Recruit-Surfactant-Extubate; INSURECLINICALRD, INSURE based on clinical respiratory distress; INSURELAMCOUNT, INSURE based on centrifuged gastric lavage lamellar body counts; LISA, less invasive surfactant administration; LISACLINICALRD, LISA based clinical respiratory distress; LISALUS, LISA guided by lung ultrasound; MVRESCUE, surfactant administration when respiratory failure is diagnosed followed by continued mechanical ventilation; MVRESCUE_LATE, surfactant administration on mechanical ventilation when a particular mean airway pressure or FiO_2_ threshold is reached; MVF, surfactant administration when a particular FiO_2_ threshold is reached followed by continued mechanical ventilation; NMA, network meta-analysis; CoE for NMA estimates that were statistically significant are depicted in bold fonts.

##### Metaregression

While none of the comparisons showed statistically significant differences for the outcome of IMV in preterm neonates of gestation 24 weeks and 28 weeks, the results were similar to that of the primary analysis at 30 weeks’ gestation (eFigure S3).

##### Neonates >30 weeks

There were two subnetworks for the outcome of IMV.

###### Subnetwork 1

**Fig. 3:**
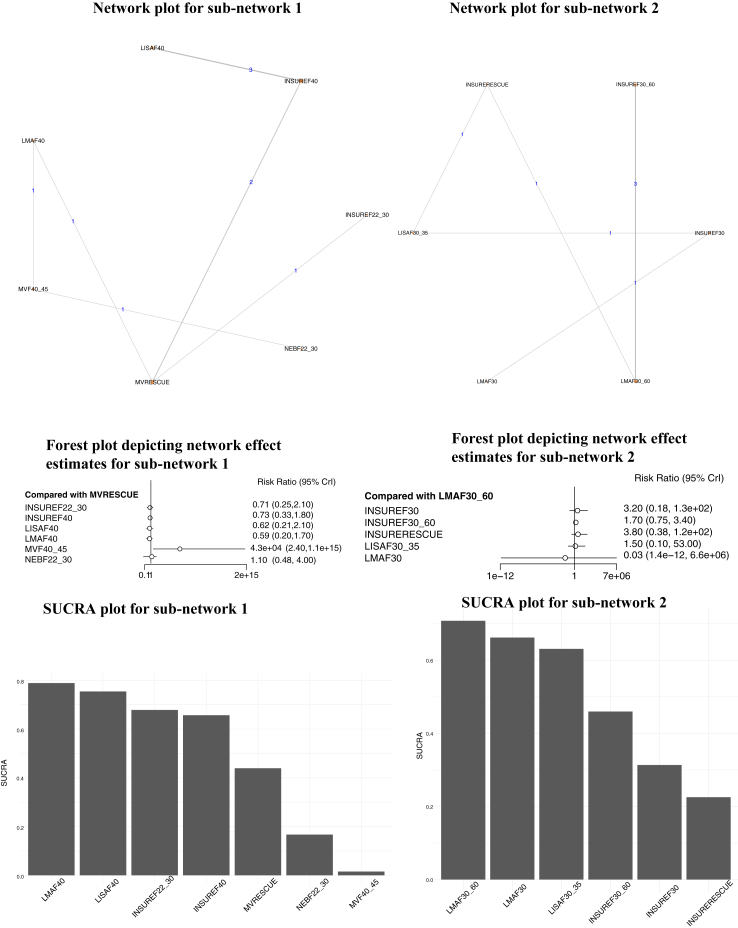
Network plot, Forest plot depicting the network effect estimates and SUCRA plot for the primary outcome of requirement of Invasive Mechanical Ventilation in preterm neonates >30 weeks' gestation for the two sub-networks.

The following interventions were evaluated: Surfactant administration through a laryngeal mask airway (LMA) at FiO_2_ threshold of 40% (LMAF40), LISAF40, INSUREF40, INSURE at FiO_2_ of 22–30% (INSUREF22_30), nebulized surfactant at FiO_2_ of 22–30% (NEBF22_30), surfactant administration at FiO_2_ of 40–45% followed by continued mechanical ventilation (MVF40_45), surfactant administration when respiratory failure was diagnosed followed by continued mechanical ventilation (MVRESCUE) (Fig. 3, eTables S4 and S5).

NMA indicated that INSUREF22_30 [0.00 (0.00–0.32)], LISAF40 [0.00 (0.00–0.29)] and INSUREF40 [0.04 (0.00–0.32)] possibly resulted in lesser risk of IMV when compared to MVF40_45. Similarly, all these interventions possibly reduced the risk of IMV when compared to NEBF22_30. Further, MVRESCUE was probably associated with decreased risk of IMV when compared to MVF40_45 (Fig. 3, eTable S6). SUCRA rated LMAF40 (0.79), LISAF40 (0.75) and INSUREF22_30 (0.68) as the three most efficacious interventions (Fig. 3). The CoE was moderate for the comparisons: LMAF40 vs. MVRESCUE, LMAF40 vs. MVF40_45, NEBF22_30 vs. MVRESCUE, NEBF22_30 vs. MVF40_45 and MVF40_45 vs. MVRESCUE. For all the other comparisons the CoE was mostly very low.

##### Metaregression

While at gestational ages of 32 weeks and 34 weeks, the results were similar to that of the primary analysis, at 36 weeks, there was no statistically significant difference between MVF40_45 and MVRESCUE (eFigure S4).

###### Subnetwork 2

Six interventions were evaluated in this network: LMAF30, LMAF30_60, LISAF30_35, INSUREF30, INSUREF30_60 and INSURERESCUE. For the comparison, INSUREF30 vs. LISAF30_35 for which CoE was moderate, there was probably no difference. Clinical benefit or harm could not be ruled out for any of the other comparisons (eFigures S5–S7, eTables S7 and S8).

### Mortality

#### Neonates ≤30 weeks

INSUREaAO_2_less0_36 possibly was associated with lesser mortality when compared to surfactant administration at FiO_2_ 30% followed by continued mechanical ventilation (MVF30) [0.00 (0.00–0.40)], MVRESCUE [0.06 (0.00–0.94)] and MVRESCUE_LATE [0.01 (0.00–0.1.00)], CoE: very low. INSURE at FiO_2_ 45–50% (INSUREF45_50) was also possibly associated with lesser risk of mortality when compared to INSURERESCUE [0.04 (0.00–0.84), CoE: very low]. LISA, based on clinical respiratory distress assessment (LISACLINICALRD) possibly decreased the risk of mortality when compared to MVF30 [0.04 (0.00–0.11), CoE: very low]. Further, when compared to CPAP alone without any surfactant administration, LISACLINICALRD was possibly associated with decreased mortality [0.04 (0.00–0.91), CoE: very low]. There were no statistically significant differences between the other comparisons (eFigures S8–S10; eTables S9–S11).

##### Metaregression

The findings were similar to the primary analysis at different gestational ages (eFigure S11).

#### Neonates >30 weeks

5 interventions were evaluated: INSUREF22_30, INSUREF40, LISAF40, INSURECLINICALRD and MVRESCUE. No statistically significant differences were found between the interventions (eFigures S12 and S13 and eTables S12–S14). No additional analysis was conducted due to lack of data.

### Mortality or BPD

NMA was done including all preterm neonates as the network was sparse and sub-group analysis based on gestational age was not feasible. No statistically significant differences were found between any of the comparisons (eFigures S14 and S15, eTables S15–S17). Inconsistency assessment and metaregression was not feasible due to the sparseness of the network.

### IVH grade >2

#### Neonates ≤30 weeks

15 interventions which included 25 studies and 6054 neonates were analyzed in the NMA. No statistically significant differences were found between any of the comparisons for this outcome (eFigures S16 and S17, eTables S18–S20).

##### Metaregression

The results of the NMA were similar to that of the primary analysis at different gestational ages (eFigure S18).

#### Neonates >30 weeks

Likewise for the other sub-group of preterm neonates, there were no statistically significant differences between any of the comparisons (eFigures S19 and S20, eTables S21–S23).

### Air leak

Similar to the outcome of mortality or BPD, NMA was performed for air leak including preterm neonates of all gestational ages (eFigure S21). Multiple interventions possibly resulted in lesser risk of air leak when compared to others. SUCRA rated the following interventions as the most efficacious: INSURELAMCOUNT (0.91), INSUREaAO_2_less0_36 (0.89), INSUREF22_30 (0.86) and NEBF22_30 (0.75). Inconsistency was detected in the network for which CoE for the NMA effect estimate was rated down by one level (eFigures S22 and S23, eTables S24–S26).

#### Metaregression

Whilst at gestational age of 32 weeks the results were similar to that of the primary analysis, at 28 weeks and 36 weeks, INSURECLINICALRD decreased the risk of air leak when compared to MVRESCUE_LATE (eFigure S24).

### Receipt of multiple doses of surfactant

INSURECLINICALRD, INSUREF22_30 and INSUREF30 possibly resulted in decreased risk of receipt of multiple doses of surfactant when compared to LMACLINICALRD, LMAF30 and MVF30, respectively. The CoE was predominantly low except for the comparison INSUREF30 vs. MVF30 for which it was moderate. Low CoE also suggested that INRECSUREF30 possibly resulted in lesser risk of receipt of further surfactant doses when compared to MVF30. Similarly, INSUREF30, LISAF30, LMAF30 and LISAF40 possibly resulted in lesser requirement of repeat surfactant doses when compared to MVF30, CoE being very low to moderate. Very low CoE indicated that LISAF40 possibly resulted in decreased risk of repeat surfactant doses when compared to LMACLINICALRD, CoE being very low. No inconsistency was detected in node-splitting (eFigures S25–S27, eTables S27–S29).

#### Metaregression

LMACLINICALRD was also shown to decrease the risk of requirement of repeat doses of surfactant when compared to MVRESCUE_LATE at higher gestational ages of 32 weeks and 36 weeks, but not at 28 weeks (eFigure S28).

The narrative review of 5 studies is provided in Annexure 2.

## Discussion

This systematic review and NMA synthesized data from 53 studies. Since there were only a few RCTs that had compared the various thresholds, the network estimates were predominantly derived from indirect evidence. Further, the results were analyzed separately wherever feasible for two sub-groups of preterm neonates with a gestational age cut-off of 30 weeks to avoid intransitivity as the incidence of the outcomes analyzed might be different in these sub-groups.

Our overall interpretation of the results indicates that when adjusted for the modality of surfactant administration (e.g., LISA, INSURE), an aAO_2_ of <0.36 was associated with decreased risk of IMV compared to various other thresholds in preterm neonates of ≤30 weeks’ gestation with RDS. An aAO_2_ of 0.36 corresponds to an FiO_2_ of 37–55%. Similarly, an FiO_2_ of 40–45% was associated with lesser risk of mortality when compared to a higher FiO_2_ in this sub-group of preterm neonates. Further, evaluation of different modalities of surfactant suggested that less invasive modalities of surfactant administration at various thresholds except for nebulized surfactant administration was associated with decreased risk of IMV when compared to surfactant administration via endotracheal tube followed by continued mechanical ventilation for preterm neonates with RDS.

The recent Cochrane review had suggested that LISA when compared to INSURE resulted in lesser risk of risk of death or BPD, need for assisted breathing in the first 72 hours of life, severe brain bleeding, death during first hospitalisation, and BPD among survivors, the results being different from that of ours’.^77^ The difference in results between the findings of our NMA and the Cochrane review could be attributed to many reasons. Firstly, the literature search in the Cochrane review was until September 2020 and the largest RCT published till date was not included.^28^ Secondly, the study population and the threshold for surfactant administration varied between the studies included in the Cochrane review. Thirdly, sub-group analyses based on gestational age was not performed due to lack of data. Finally, since ours’ was an NMA and the Cochrane meta-analysis was a pairwise one, the addition of indirect evidence in the NMA in addition to the direct evidence from pairwise meta-analysis would have modified the effect estimates.

Amongst the neonates born at ≤30 weeks, SUCRA ranked aAO_2_ < 0.36 was ranked as the best intervention. However, irrespective of the less invasive modality utilized (INSURE or LISA), clinical benefit or harm could not be ruled out for FiO_2_ of 30% vs. 40%. Since these levels have been widely studied and have been recommended as thresholds for surfactant administration, we suggest future trials comparing them.^78^ We acknowledge that in times where peripheral oxygen saturation measurement via pulse-oximetry is widely available, arterial blood gas analysis is not as frequently performed and clinical teams are not universally confident in interpreting aAO_2_ data from these. Therefore, future studies are needed to link FiO_2_ and aAO_2_ in the context of establishing the surfactant need. Choosing a threshold higher than these might not be appropriate as obliquely indicated by the comparison INSURE at aAO_2_ < 0.36 vs. INSURE as a rescue measure where the former was possibly shown to be beneficial. Metaregression indicated that in neonates born at threshold of viability such as at 24 weeks, clinical benefit or harm could not be ruled out for either the various thresholds or the mode of surfactant administration. In a survey conducted by International Network for Evaluating Outcomes (iNEO), it was reported that most of the countries preferred the invasive approach of continuing IMV in neonates born at 23–24 weeks.^79^ Since improving the intact survival of neonates born at threshold of viability is a major focus of neonatal medicine as of present, future trials are warranted in this sub-group of preterm neonates as well.^80^ Earlier surfactant administration, as informed by LUS may warrant further investigation. Though promising, at present, trials have not shown LUS to have additional benefits over FiO_2_ in evaluating the severity of RDS and surfactant need.^11^^,^^81^ Though SUCRA was used to rank the various interventions across outcomes, it has some limitations. SUCRA values can vary across outcomes for the same regimen, the differences might be due to chance alone as it does not account for the 95% CI, and they do not capture the magnitude of differences in effects between interventions.

Further, surfactant administration through LISACLINICALRD was shown to decrease mortality when compared to the use of only CPAP. This finding might be of relevance in resource limited settings where blended oxygen is not available, and the clinicians must depend upon clinical respiratory distress scoring. Though such scores have been developed, surfactant availability and training of health care personnel might be bottle necks.^12^ For the other secondary outcomes of air leak and receipt of repeated doses of surfactant, NMA indicated that earlier surfactant administration at FiO_2_ of 22–30%, aAO_2_ < 0.36 and that guided by lamellar body counts might possibly be beneficial.

Our NMA indicated that the recently evaluated strategy of strategy Intubate-Recruit-Surfactant-Extubate was not superior to the traditionally used INSURE technique. Two trials evaluating INRECSURE were included in the NMA.^67^^,^^70^ It is to be noted that the trial by Vento et al. which had utilized high frequency oscillation ventilation (HFOV) for recruitment had shown a decreased risk of IMV in extremely low gestational age group neonates (ELGANs).^67^ The probable reason for the discrepancy between our results and that of the trial might be attributed to the addition of indirect evidence in the NMA analysis. Also, the possibility of intransitivity could not be ruled out as Vento et al. had exclusively evaluated ELGANs who were sicker compared to those infants included in the other trials. Further, the patient population and the recruitment technique utilized by these two trials were different. Whilst Vento et al. had utilized HFOV for lung recruitment, Yang et al. used sustained lung inflation in conventional mechanical ventilation in a more mature group of preterm infants.^70^ Though we had combined both these interventions as INRECSURE, we had down rated the evidence certainty by one level due to indirectness related to the intervention and patient population. Finally, the ongoing RCT (INREC-LISA trial, NCT05711966) would provide further insights regarding the efficacy of INRECSURE.

For the sub-group of neonates born at >30 weeks’ gestation, NMA also showed that rescue surfactant administration might possibly be more beneficial than using a FiO_2_ threshold of 40–45%, indirectly indicating that a higher threshold may be preferred in this sub-group. This contrasts with the finding that in preterm neonates of ≤30 weeks, INSURE at FiO_2_ of 40–45% was possibly associated with decreased mortality when compared to INSURE as rescue.

Very few studies have interrogated nebulized surfactant for preterm infants with RDS. A systematic review by Gaertner et al. concluded that nebulized surfactant might possibly decrease the risk of IMV in preterm neonates of <37 weeks’ gestation with the CoE being low.^82^ However, in preterm neonates >30 weeks’ gestation, this NMA indicated that use of nebulized surfactant at a lower FiO_2_ threshold of 22–30% might possibly increase the risk of IMV when compared to other lesser invasive modalities at FiO_2_ 22–30% and 40%. A plausible explanation could be that nebulized surfactant might not reach the alveolar space in sufficient quantity to be effective and thus, trials of nebulised surfactant might have delayed the delivery of surfactant administration through other proven modalities. One important reason we postulate for the discrepancy between our findings and that of Gaertner et al. could be that the control group included in Gaertner et al.’s meta-analysis was heterogenous and included preterm neonates who required no treatment, CPAP alone, or a more invasive surfactant administration method. The control groups in our study predominantly included preterm neonates who were diagnosed with RDS and had received surfactant through various modalities at different thresholds. More refined technologies for surfactant nebulisation are required and further clinical trials needed to confidently advise surfactant nebulisation. The optimal threshold for use of surfactant in late preterm neonates is still debated. Previously, different interpretations of evidence supporting surfactant treatment at defined FiO_2_ thresholds have led to different clinical guidance, which may contribute to confusing clinical practice and possibly deleterious outcomes. More studies are needed to define the optional thresholds for surfactant therapy, tailored to the underlying pathology, disease process and means of applied respiratory support.^5^ At least for late preterm and early term infants, one large RCT is currently ongoing.^83^

The main strength of this NMA is that it is the only one conducted to date comprehensively evaluating the different thresholds utilized along with the modality of surfactant administration in preterm neonates with RDS. It allowed the comparison of thresholds that have not been evaluated in RCTs before. The reporting of this systematic review is in accordance with the PRISMA NMA. Further, we followed strict GRADE recommendations for assessing the CoE for the NMA. There were also some limitations. Though we took great care in assessing intransitivity addressing important factors such as gestational age and modality of surfactant administration, there still could have been intransitivity related to the type and dosage of surfactant utilized, the different CPAP levels and the wide time span across which the studies were conducted during which there have been significant advances in all areas of perinatal and neonatal care. Finally, the results of this NMA may not be generalizable to those neonates born at the threshold of viability at gestations of 22–24 weeks’ as only a very few studies have included them and is an area of future research.

The results of the NMA indicate that using lesser invasive surfactant administration approaches such as INSURE and LISA with the threshold of aAO_2_ < 0.36 (corresponding to an FiO_2_ of 37–55%) might possibly be beneficial in preterm neonates of <30 weeks’ gestation. Similarly, for the outcome of mortality, after adjusting for the effect modifier of the mode of surfactant administration, an FiO_2_ 40–45% was shown be associated with decreased mortality when compared to a higher threshold. Henceforth, we suggest surfactant administration in preterm neonates of ≤30 weeks’ with RDS who require an FiO_2_ of ≥40%. We do caution that this suggestion is based on very low to low CoE. There was insufficient evidence for the comparison of the FiO_2_ thresholds of 30% vs. 40%, warranting future trials. The cost-benefit-ratio for giving surfactant at lower thresholds may need to be assessed at unit level, considering regional circumstances, which include prevalence of known aggravating risk factors for severe RDS, such as perinatal inflammation, and also treatment rates with antenatal corticosteroid. In low resource settings where blended oxygen is not always available and FiO_2_ requirement cannot be adjudged, LISA, using clinical respiratory distress scoring, might possibly result in decreased risk of mortality when compared to the use of CPAP alone. Whilst the three NMAs published till date had evaluated either the modality of surfactant administration or the different FiO_2_ cut-off levels, unlike this NMA none had studied these aspects wholesomely as a single intervention which could circumvent the fundamental assumption of an NMA which is intransitivity.^84^, ^85^, ^86^

Further research into optimising surfactant treatment for RDS should focus on practical surrogates of disease severity, taking into consideration infants’ gestational age, co-morbidities and predisposing factors, modality and level of respiratory support, and FiO_2_. In this context, lung ultrasound may be a very promising tool to refine the surfactant need. Also, refinement of nebulization techniques to ensure effective surfactant deposition into the alveoli is warranted. Finally, future trials and meta-analyses may include a homogenous population of ELGANs as evidence base pertaining to this sub-group is sparse.

## Contributors

Drs VVR, CCR and TS conceptualized the systematic review and meta-analysis. AKPS devised the literature search strategy and was involved in the data extraction process. VVR, TB and TA verified and analyzed the data. VVR and CJW produced the initial draft. CCR, TB, TA, AKPS and TS provided further intellectual inputs and revised the initial draft. All authors had full access to all the data in the study and had final responsibility for the decision to submit for publication.

## Data sharing agreement

All data is provided in full in the results section of this paper. The relevant original source documents are cited in full in the reference section. The data can be shared with others on request via email to the corresponding author of this manuscript without any restrictions.

## Declaration of interests

We declare no competing interests.

## Appendix A Supplementary data

eFigures S1–S28, eTables S1–S29, and Annexures 1 and 2
