# Supplementary material for: Clinical decision thresholds for surfactant administration in preterm infants: a systematic review and network meta-analysis
Source: eClinicalMedicine. 2023 Jul 20;62:102097. doi: 10.1016/j.eclinm.2023.102097 (PMC10393620; doi:10.1016/j.eclinm.2023.102097)
Supplement: eFigures S1–S28, eTables S1–S29, and Annexures 1 and 2 [file mmc1.pdf]

Supplementary online content

eFigure 1: Direct evidence from pairwise meta-analysis for the outcome requirement of invasive mechanical ventilation (IMV) for the sub-group ≤30 weeks
eFigure 2: Inconsistency assessment for the outcome requirement of IMV
eFigure 3: Metaregression at different gestational ages for the outcome requirement of IMV for the sub-group ≤30 weeks
eFigure 4: Metaregression at different gestational ages for the outcome requirement of IMV for the sub-group >30 weeks for sub-network 1
eFigure 5: Network plot, Forest plot depicting the network effect estimates and SUCRA plot for the outcome of requirement of IMV in preterm neonates > 30 weeks' gestation for sub-network 2
eFigure 6: Direct evidence from pairwise meta-analysis for the outcome requirement of IMV for the sub-group >30 weeks for sub-network 1
eFigure 7: Direct evidence from pairwise meta-analysis for the outcome requirement of IMV for the sub-group >30 weeks for sub-network 2
eFigure 8: Network plot, Forest plot depicting the network effect estimates and SUCRA plot for the outcome of mortality in preterm neonates ≤ 30 weeks
eFigure 9: Direct evidence from pairwise meta-analysis for the outcome of mortality for the sub-group ≤30

weeks
eFigure 10: Inconsistency assessment for the outcome of mortality
eFigure 11: Metaregression at different gestational ages for the outcome of mortality for the sub-group ≤30 weeks
eFigure 12: Network plot, Forest plot depicting the network effect estimates and SUCRA plot for the outcome of mortality in preterm neonates > 30 weeks' gestation
eFigure 13: Direct evidence from pairwise meta-analysis for the outcome of mortality for the sub-group >30 weeks
eFigure 14: Network plot, Forest plot depicting the network effect estimates and SUCRA plot for the outcome of mortality or bronchopulmonary dysplasia (BPD) in preterm neonates ≤ 36 weeks
eFigure 15: Direct evidence from pairwise meta-analysis for the outcome of mortality or BPD in preterm neonates ≤ 36 weeks
eFigure 16: Network plot, Forest plot depicting the network effect estimates and SUCRA plot for the outcome of Intraventricular Hemorrhage (IVH) > Grade 2 in preterm neonates ≤ 30 weeks
eFigure 17: Direct evidence from pairwise meta-analysis the outcome of IVH > Grade 2 in preterm neonates ≤ 30 weeks'
eFigure 18: Metaregression at different gestational ages for the outcome of IVH > Grade 2 for the sub-group ≤

30 weeks
eFigure 19: Network plot, Forest plot depicting the network effect estimates and SUCRA plot for the outcome of IVH > Grade 2 in preterm neonates > 30 weeks
eFigure 20: Direct evidence from pairwise meta-analysis for the outcome of Intraventricular Hemorrhage (IVH) > Grade 2 in preterm neonates > 30 weeks
eFigure 21: Network plot, Forest plot depicting the network effect estimates and SUCRA plot for the outcome of air leak in preterm neonates ≤ 36 weeks
eFigure 22: Inconsistency assessment for the outcome of air leak in preterm neonates ≤ 36 weeks
eFigure 23: Direct evidence from pairwise meta-analysis for the outcome of air leak in preterm neonates ≤ 36 weeks
eFigure 24: Metaregression at different gestational ages for the outcome of air leak in preterm neonates ≤ 36 weeks' gestation
eFigure 25: Network plot, Forest plot depicting the network effect estimates and SUCRA plot for the outcome of receipt of multiple doses of surfactant in preterm neonates ≤ 36 weeks
eFigure 26: Inconsistency assessment for the outcome of receipt of multiple doses of surfactant in preterm neonates ≤ 36 weeks
eFigure 27: Direct evidence from pairwise meta-analysis for the outcome of receipt of multiple doses of

surfactant in preterm neonates ≤ 36 weeks
eFigure 28: Metaregression at different gestational ages for the outcome of receipt of multiple doses of surfactant in preterm neonates ≤ 36 weeks
eTable 1: Literature search strategy
eTable 2: Risk of bias assessment of the included studies
eTable 3: Network characteristics for the outcome of requirement of IMV for the sub-group ≤30 weeks
eTable 4: Network characteristics for the outcome of requirement of IMV for the sub-group >30 weeks for subnetwork 1
eTable 5: Network characteristics for the outcome of requirement of IMV for the sub-group >30 weeks for subnetwork 2
eTable 6: Matrix plot depicting the network effect estimates for the outcome of requirement of IMV for the sub-group >30 weeks for subnetwork 1
eTable 7: Matrix plot depicting the network estimates effect estimate for the outcome of requirement of IMV for the sub-group >30 weeks for subnetwork 2
eTable 8: Certainty of evidence (CoE) for various comparisons for the outcome of IMV for the sub-group >30 weeks for sub-network 1 and 2
eTable 9: Network characteristics for the outcome of mortality for the sub- group ≤ 30 weeks
eTable 10: Matrix plot depicting the network effect estimates for the outcome of mortality for the sub-group ≤ 30 weeks

eTable 11: Certainty of evidence (CoE) for various comparisons for the outcome of mortality for the sub-group ≤ 30 weeks
eTable 12: Network characteristics for the outcome of mortality for the sub-group > 30 weeks
eTable 13: Matrix plot depicting the network estimates for the outcome of mortality for the sub-group > 30 weeks
eTable 14: CoE for various comparisons for the outcome of mortality for the sub-group > 30 weeks
eTable 15: Network characteristics for the outcome of mortality or BPD in preterm neonates ≤ 36 weeks' gestation
eTable 16: Matrix plot depicting the network estimates for the outcome of mortality or BPD in preterm neonates ≤ 36 weeks' gestation
eTable 17: CoE for various comparisons for the outcome of mortality or BPD in preterm neonates ≤ 36 weeks' gestation
eTable 18: Network characteristics for the outcome of IVH for the sub- group ≤ 30 weeks
eTable 19: Matrix plot depicting the network estimates for the outcome of IVH for the sub-group ≤ 30 weeks
eTable 20: CoE for various comparisons for the outcome of mortality for the sub-group ≤ 30 weeks
eTable 21: Network characteristics for the outcome of IVH for the sub- group > 30 weeks
eTable 22: Matrix plot depicting the network estimates for the outcome of IVH for the sub-group > 30 weeks

eTable 23: CoE for various comparisons for the outcome of mortality for the sub-group > 30 weeks
eTable 24: Network characteristics for the outcome of air leak in preterm neonates ≤ 36 weeks' gestation
eTable 25: Matrix plot depicting the network estimates for the outcome of air leak in preterm neonates ≤ 36 weeks' gestation
eTable 26: CoE for various comparisons for the outcome of air leak in preterm neonates ≤ 36 weeks' gestation
eTable 27: Network characteristics for the outcome of receipt of repeated doses of surfactant in preterm neonates ≤ 36 weeks' gestation
eTable 28: Matrix plot depicting the network estimates for the outcome of receipt of repeated doses of surfactant in preterm neonates ≤ 36 weeks' gestation
eTable 29: CoE for various comparisons for the outcome of receipt of repeated doses of surfactant in preterm neonates ≤ 36 weeks' gestation
Annexure 1: Interventions evaluated in the network meta-analysis Annexure 2: Narrative review of the included the studies in the systematic review

eFigure 1: Direct evidence from pairwise meta-analysis for the outcome requirement of invasive mechanical ventilation (IMV) for the sub-group ≤30 weeks

Study	TE	seTE	Experimental Total	Control Total	RR	95%-CI
INSUREF40:INSUREFCLINICALRD						
Dilmen 2013	0.25	0.2813	80	79	1.28	[0.73; 2.22]
MVRESCUE:MVRESCUE_LATE						
Gortner 1998	0.03	0.0830	154	163	1.03	[0.87; 1.21]
INSURERESCUE:LISAF30						
Gopel 2011	0.79	0.1476	112	108	2.20	[1.64; 2.93]
INSURECLINICALRD:CPAP_only						
Imani 2013	−0.41	0.6055	40	40	0.67	[0.20; 2.18]
INSURECLINICALRD:INSUREF45_50						
Kandraju 2012	−0.67	0.3117	74	79	0.51	[0.28; 0.94]
LISAF30:LISALUS						
Rodriguez−Fanjul 2020	0.69	0.4807	29	29	2.00	[0.78; 5.13]
INSUREaAO2less_0_36:INSURELAMCOUNT						
Verder 2013	−0.03	0.1432	188	192	0.97	[0.74; 1.29]
INSUREF30:LISAF30						
Gupta 2020	0.69	0.6565	29	29	2.00	[0.55; 7.24]
Heidarzadeh 2013	0.37	0.5243	42	38	1.45	[0.52; 4.05]
Kribs 2015	0.28	0.0570	104	107	1.32	[1.18; 1.48]
Bao 2015	0.31	0.4248	43	47	1.37	[0.59; 3.14]
Mohammadizadeh 2015	0.41	0.8533	19	19	1.50	[0.28; 7.99]
Random effects model			**237**	**240**	**1.33**	**[1.19; 1.49]**
Heterogeneity: $I^2 = 0\%$, $\tau^2 = 0$, $p = 0.98$						
INSUREF40:LISAF40						
Kanmaz 2013	0.20	0.1594	100	100	1.23	[0.90; 1.67]
Sabzehei 2022	0.15	0.2597	56	56	1.17	[0.70; 1.94]
Boskabadi 2019	1.79	1.0328	20	20	6.00	[0.79; 45.42]
Random effects model			**176**	**176**	**1.26**	**[0.91; 1.74]**
Heterogeneity: $I^2 = 16\%$, $\tau^2 = 0.0170$, $p = 0.30$						
INSURECLINICALRD:MVRESCUE						
Rojas 2009	−0.39	0.1775	141	137	0.68	[0.48; 0.96]
INRECSUREF30:INSUREF30						
Vento 2020	−0.30	0.1469	107	111	0.74	[0.56; 0.99]
INSUREaAO2less_0_22:MVRESCUE_LATE						
Verder 1994	−0.68	0.2086	35	33	0.51	[0.34; 0.76]
INSUREaAO2less_0_22:INSUREaAO2less_0_36						
Verder 1999	0.95	0.3413	27	33	2.60	[1.33; 5.07]
INRECSURE_RESCUE:INSUREF_RESCUE						
Yang 2022	−0.51	0.2333	92	92	0.60	[0.38; 0.95]
LISAF30:MVF45						
Dargaville 2021	−0.68	0.0938	241	244	0.51	[0.42; 0.61]
INSUREF45_50:MVF45						
Nayeri 2014	−1.03	0.4198	21	21	0.36	[0.16; 0.81]

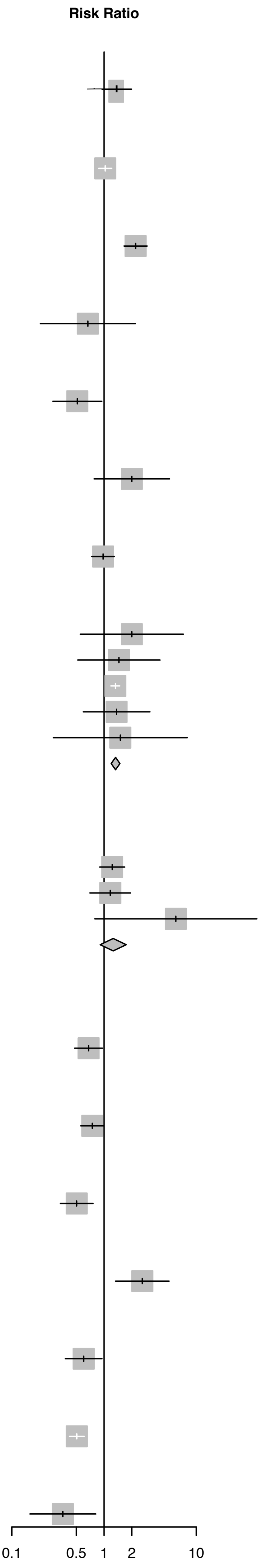

eFigure 2: Inconsistency assessment for the outcome requirement of IMV

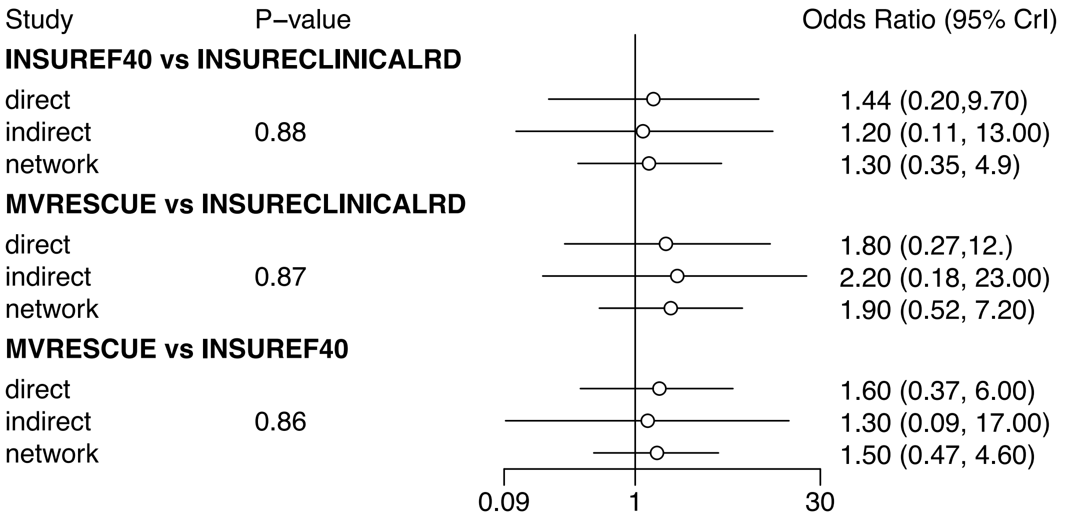

eFigure 3: Metaregression at different gestational ages for the outcome requirement of IMV for the sub-group ≤30 weeks

Gestational age: 24 weeks

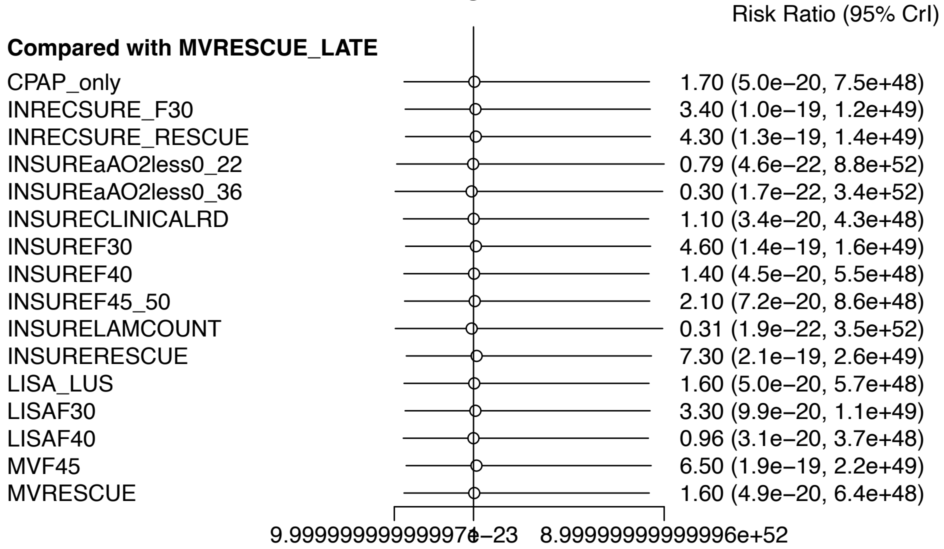

Gestational age: 28 weeks

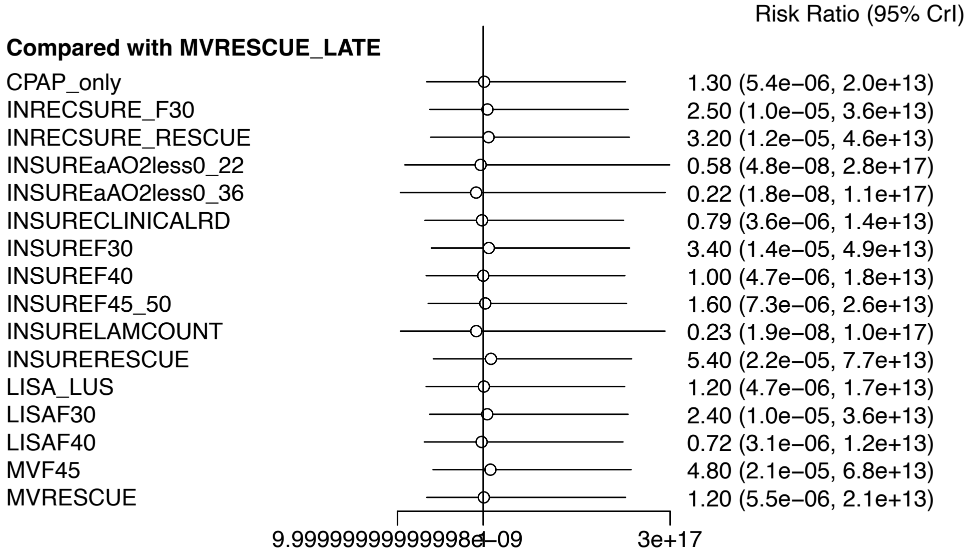

Gestational age: 30 weeks

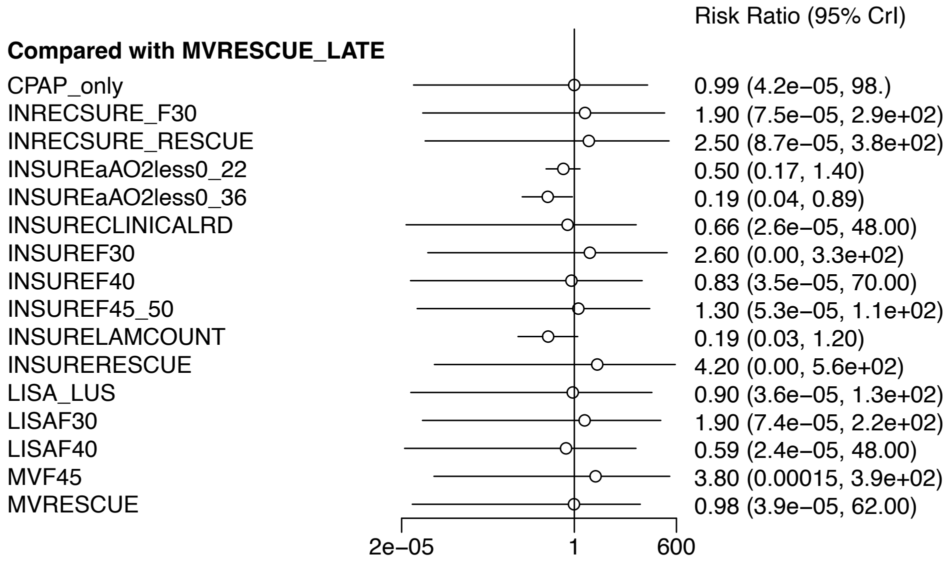

eFigure 4: Metaregression at different gestational ages for the outcome requirement of IMV for the sub-group >30 weeks for sub-network 1

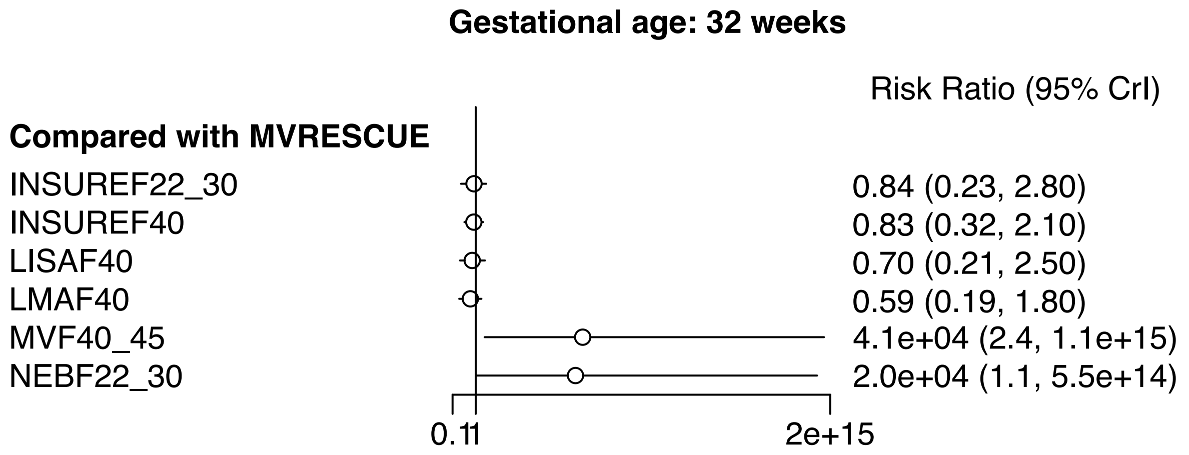

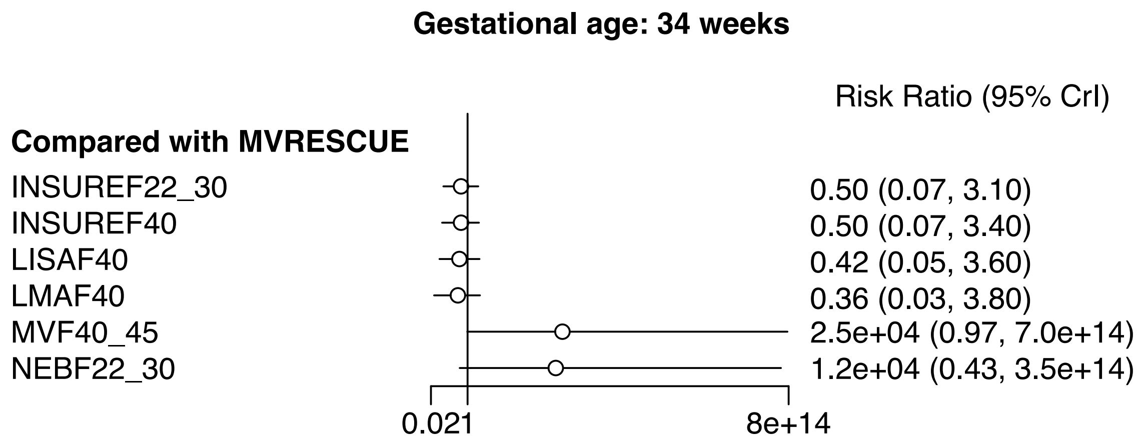

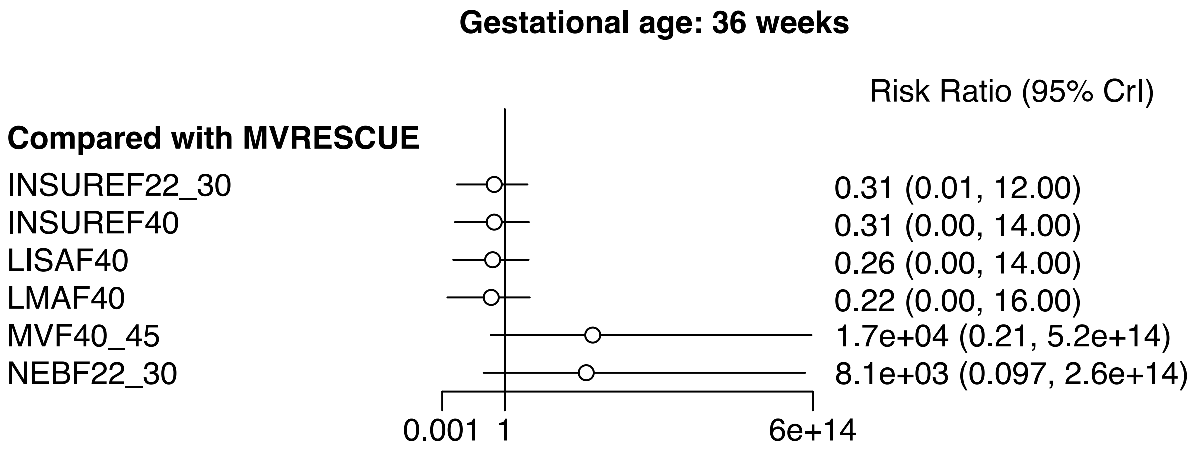

eFigure 5: Network plot, Forest plot depicting the network effect estimates and SUCRA plot for the outcome of requirement of IMV in preterm neonates > 30 weeks' gestation for sub-network 2

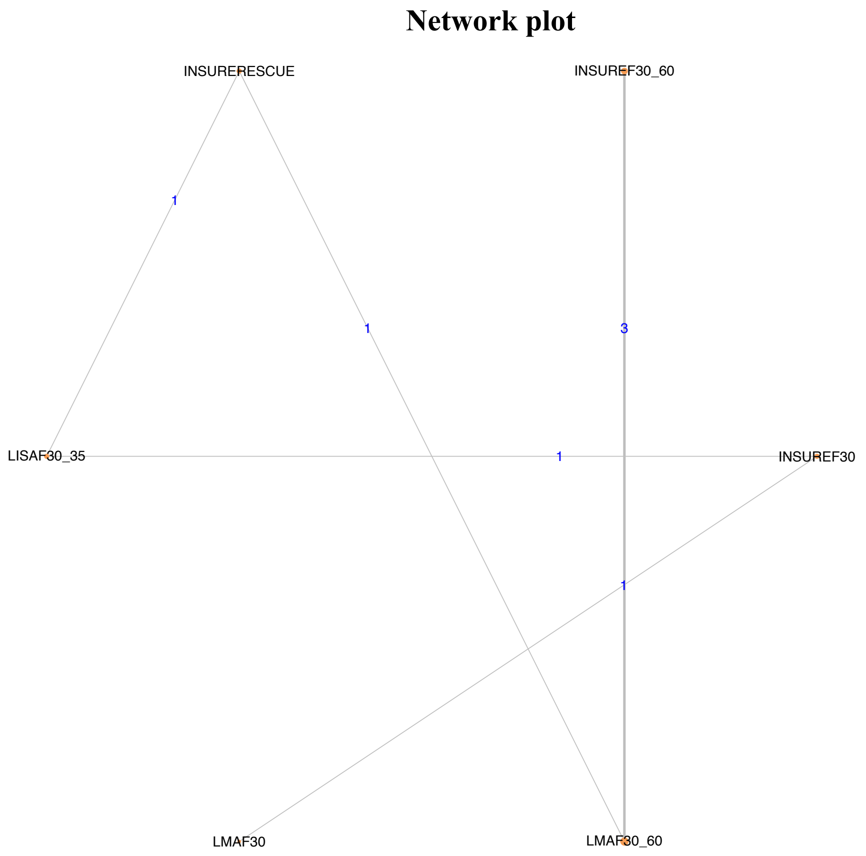

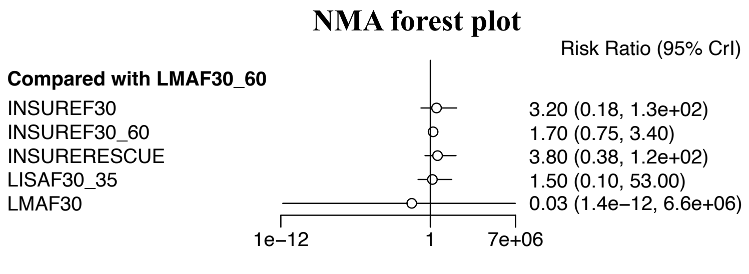

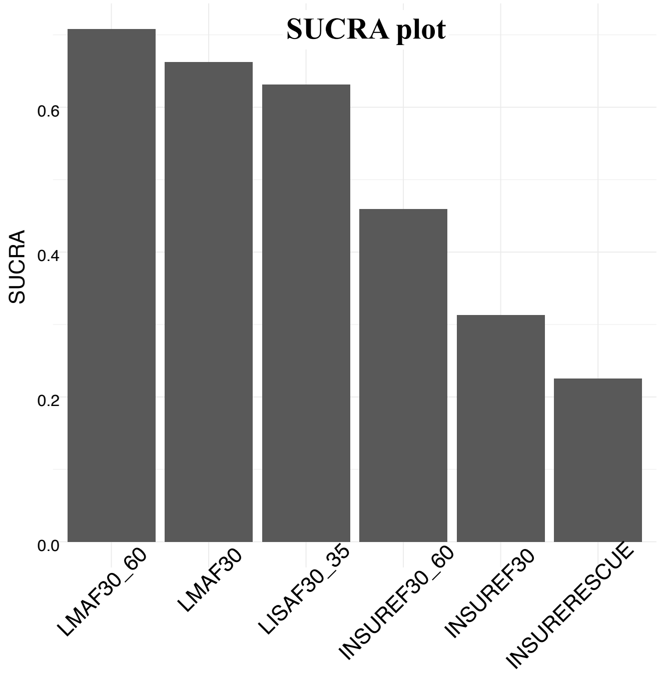

eFigure 6: Direct evidence from pairwise meta-analysis for the outcome requirement of IMV for the sub-group >30 weeks for sub-network 1

Study	TE	seTE	Experimental Total	Control Total	RR	95%-CI
**INSUREF40:MVRESCUE**						
TEXAS GROUP 2004	−0.50	0.2510	65	67	0.60	[0.37; 0.99]
Khosravi 2008	0.04	0.4013	22	23	1.05	[0.48; 2.30]
**Random effects model**			**87**	**90**	**0.73**	**[0.44; 1.21]**
Heterogeneity: $I^2 = 25\%$, $\tau^2 = 0.0382$, $p = 0.25$						
**MVF40_45:NEBF22_30**						
Minocchieri 2019	0.69	0.2718	32	32	2.00	[1.17; 3.41]
**MVRESCUE:INSUREF22_30**						
Reininger 2005	0.33	0.1655	53	52	1.40	[1.01; 1.93]
**INSUREF40:LISAF40**						
Pareek 2021	0.00	0.4830	20	20	1.00	[0.39; 2.58]
Choupani 2018	0.49	0.4043	52	52	1.62	[0.74; 3.59]
Yang 2019	−0.35	0.7362	50	47	0.70	[0.17; 2.98]
**Random effects model**			**122**	**119**	**1.21**	**[0.69; 2.12]**
Heterogeneity: $I^2 = 0\%$, $\tau^2 = 0$, $p = 0.54$						
**LMAF40:MVRESCUE**						
Roberts 2017	−0.52	0.2078	50	53	0.59	[0.39; 0.89]
**LMAF40:MVF40_45**						
Barbosa 2017	−1.03	0.2599	26	22	0.36	[0.22; 0.60]

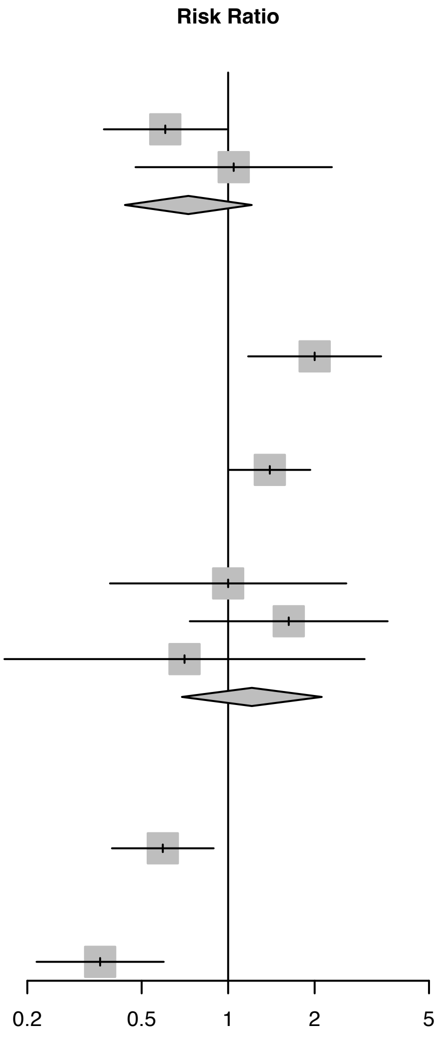

eFigure 7: Direct evidence from pairwise meta-analysis for the outcome requirement of IMV for the sub-group >30 weeks for sub-network 2

Study	TE	seTE	Experimental Total	Control Total	RR	95%–CI
**INSURERESCUE:LISAF30_35**						
Olivier 2017	0.94	0.3021	21	24	2.57	[1.42; 4.65]
**INSUREF30_60:LMAF30_60**						
Amini 2019	–0.15	0.4928	30	30	0.86	[0.33; 2.25]
Gallup 2021	0.41	0.3719	40	50	1.50	[0.72; 3.11]
Pinheiro 2015	0.94	0.2965	30	30	2.56	[1.43; 4.57]
**Random effects model**			**100**	**110**	**1.63**	**[0.90; 2.97]**
Heterogeneity: $I^2 = 49\%$, $\tau^2 = 0.1357$, $p = 0.14$						
**INSURERESCUE:LMAF30_60**						
Attridge 2012	1.10	1.0860	13	13	3.00	[0.36; 25.21]
**LMAF30:INSUREF30**						
Sadeghnia 2014	0.00	4.4657	35	35	1.00	[0.00; 6327.72]
**LISAF30_35:INSUREF30**						
Jena 2019	–0.75	0.1821	175	175	0.47	[0.33; 0.67]

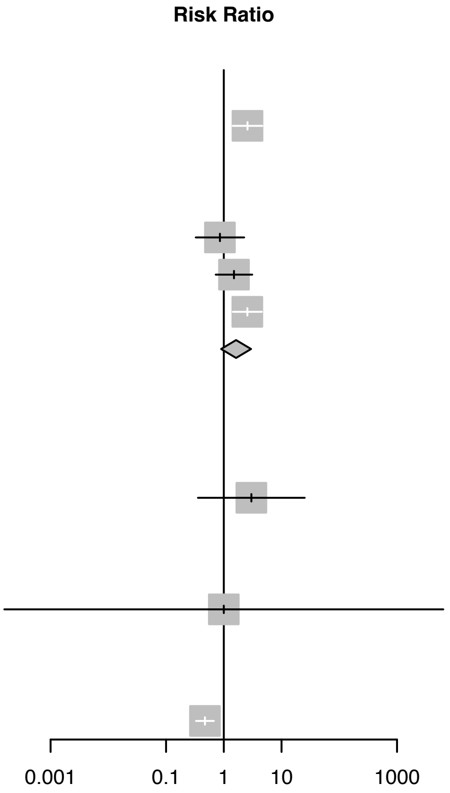

eFigure 8: Network plot, Forest plot depicting the network effect estimates and SUCRA plot for the outcome of mortality in preterm neonates ≤ 30 weeks

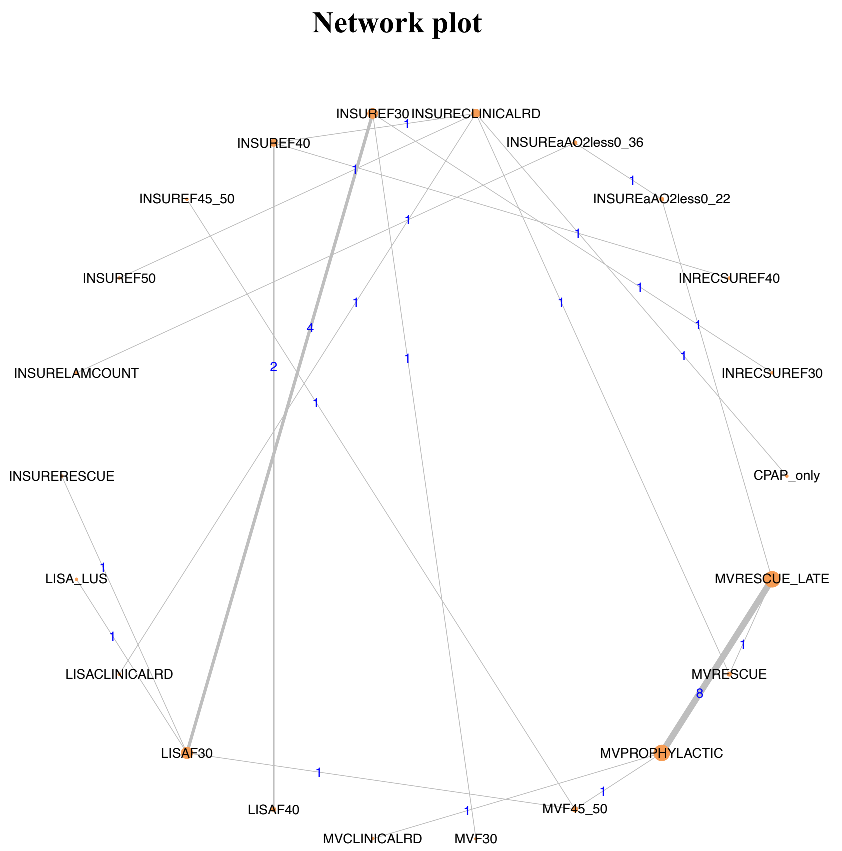

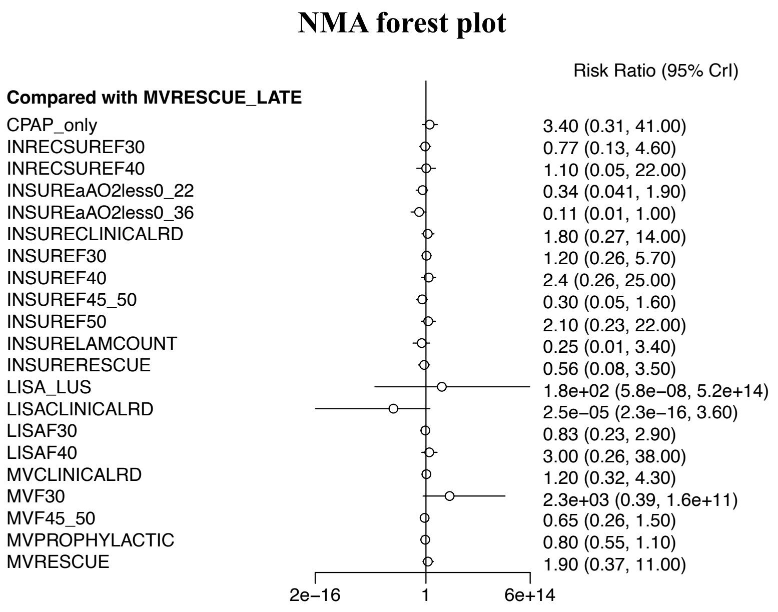

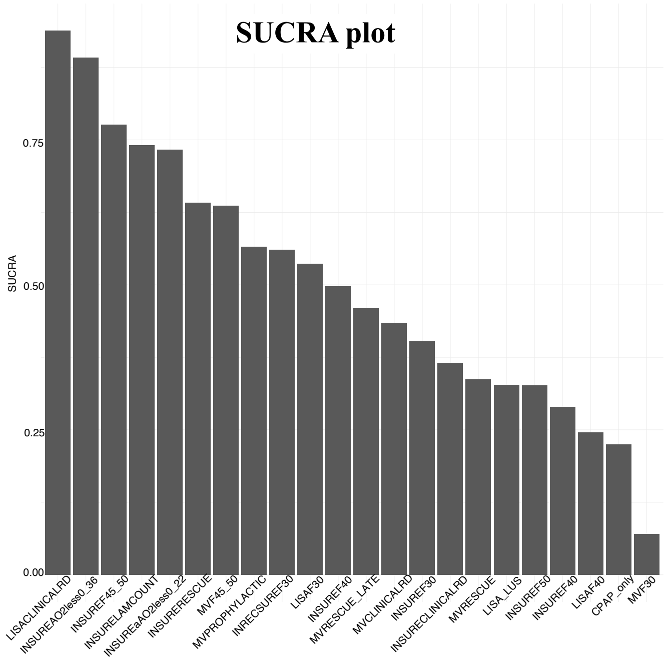

eFigure 9: Direct evidence from pairwise meta-analysis for the outcome of mortality for the sub-group ≤30 weeks

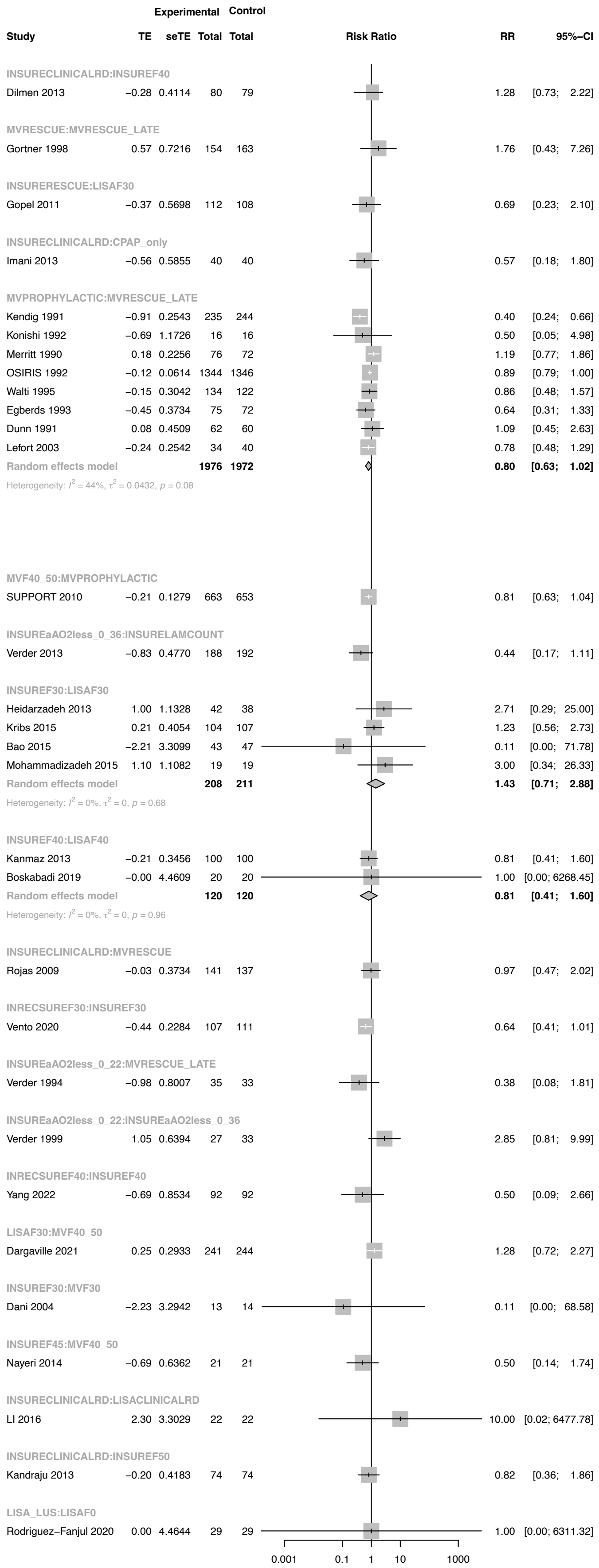

eFigure 10: Inconsistency assessment for the outcome of mortality

Study	P−value	Odds Ratio (95% CrI)
**INSUREF40 vs INSURECLINICALRD**		
direct		1.4 (0.42, 4.8)
indirect	0.79	0.96 (0.07, 14.)
network		1.3 (0.46, 3.8)
**MVRESCUE vs INSURECLINICALRD**		
direct		0.91 (0.33,2.5)
indirect	0.84	1.20 (0.09, 18.)
network		0.97 (0.37, 2.4)
**MVRESCUE vs INSUREF40**		
direct		0.94 (0.08, 10.)
indirect	0.81	0.65 (0.14, 3.1)
network		0.73 (0.20, 2.7)

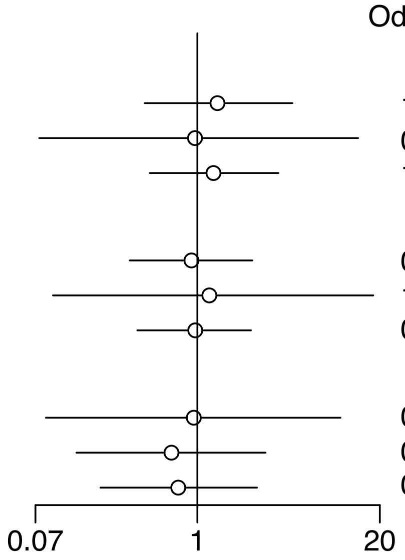

eFigure 11: Metaregression at different gestational ages for the outcome of mortality for the sub-group ≤30 weeks

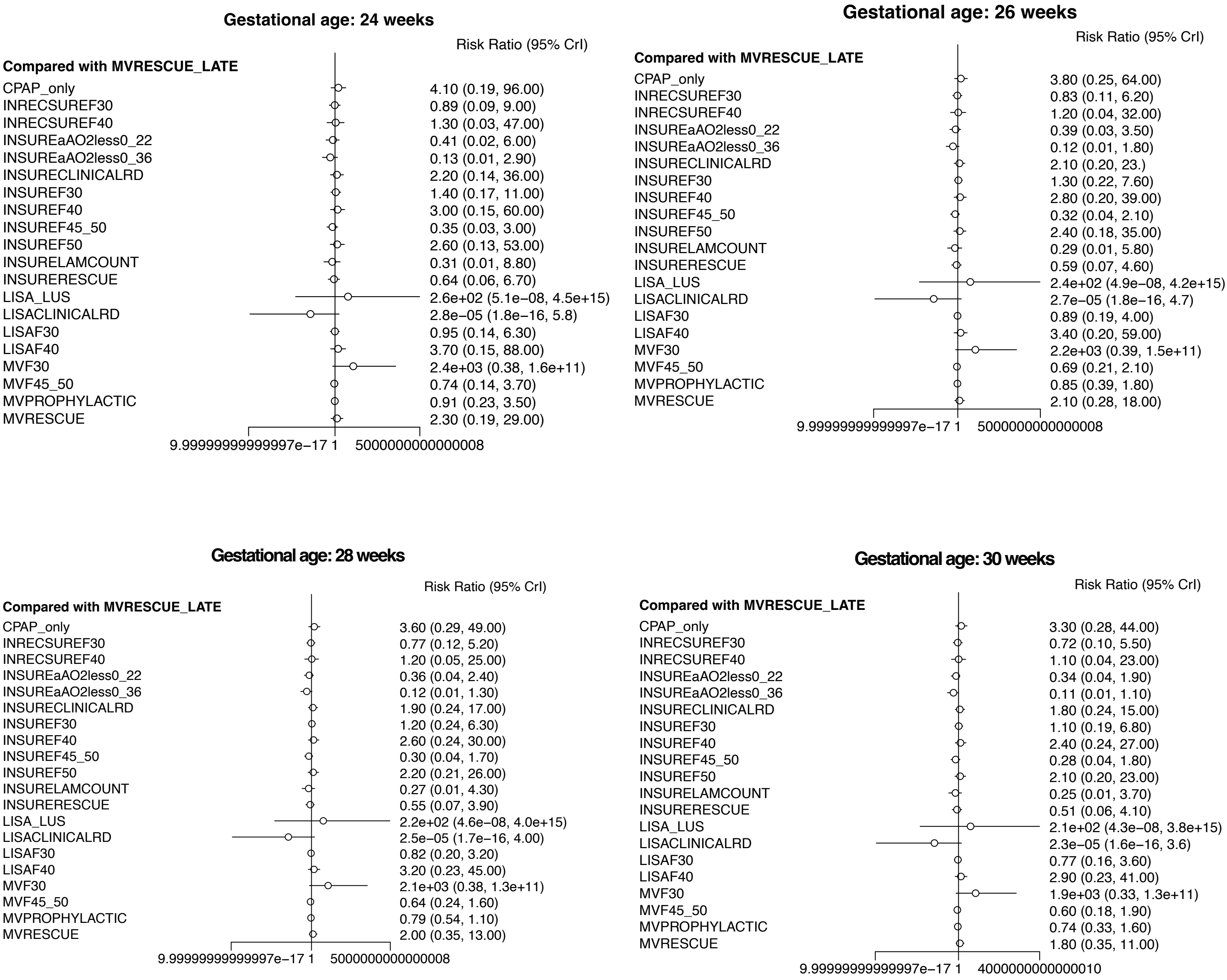

eFigure 12: Network plot, Forest plot depicting the network effect estimates and SUCRA plot for the outcome of mortality in preterm neonates > 30 weeks' gestation

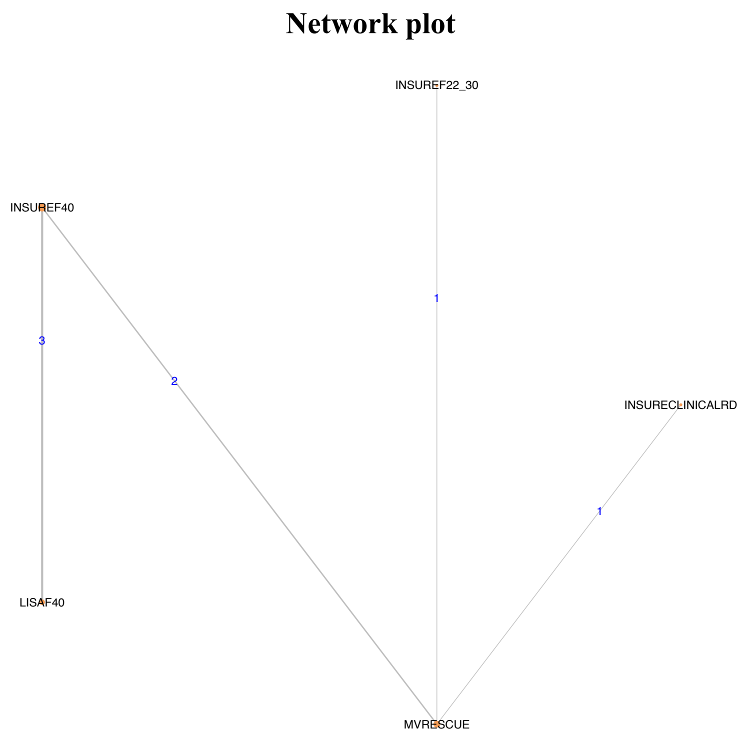

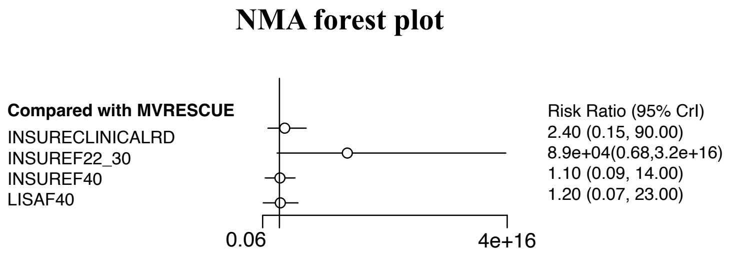

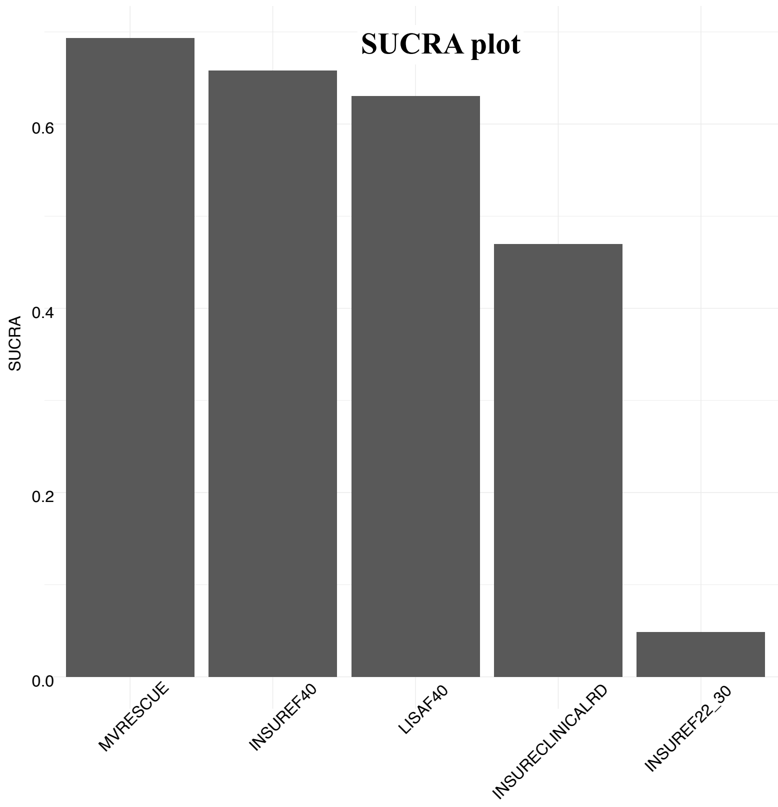

eFigure 13: Direct evidence from pairwise meta-analysis for the outcome of mortality for the sub-group >30 weeks

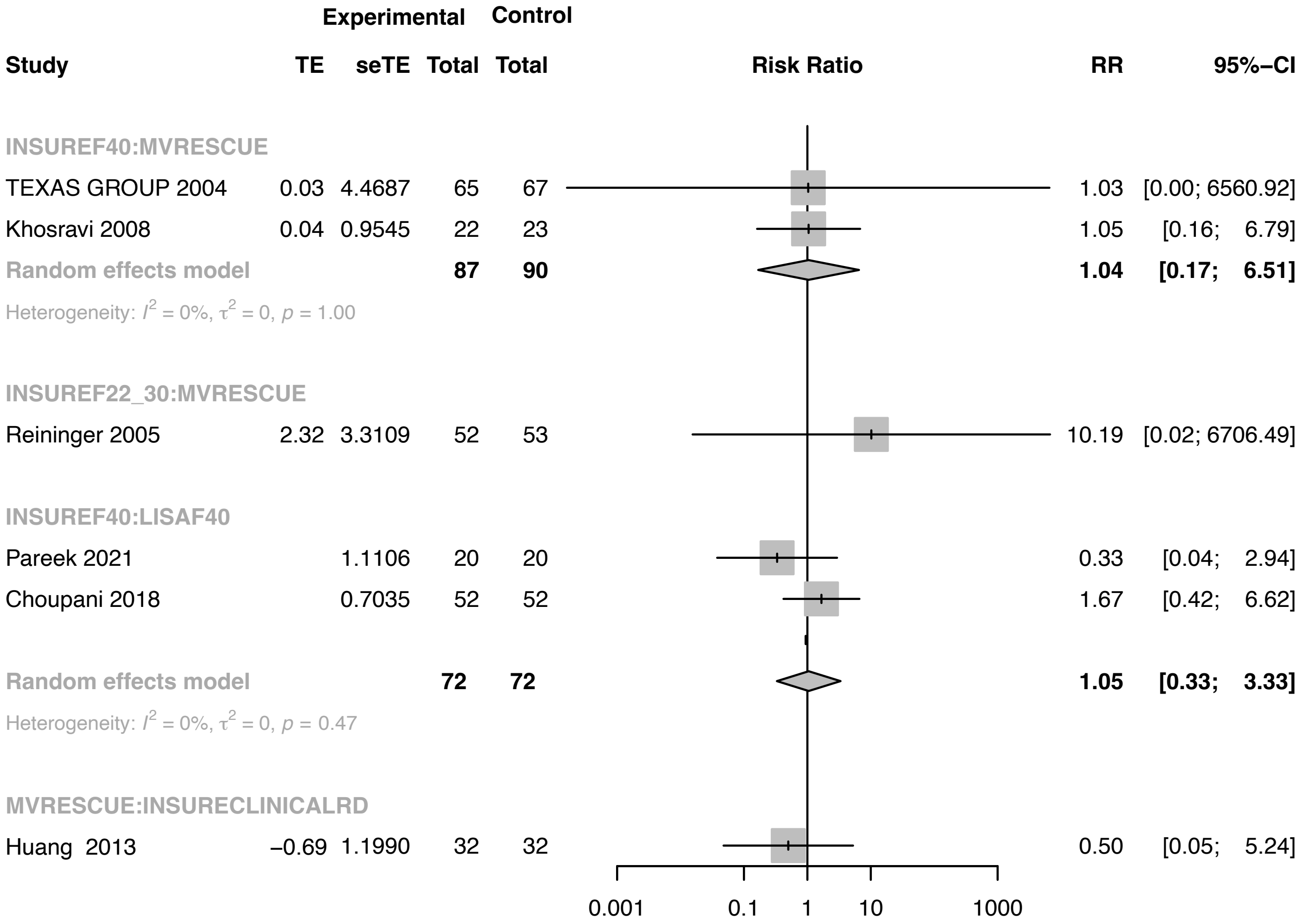

eFigure 14 : Network plot, Forest plot depicting the network effect estimates and SUCRA plot for the outcome of mortality or bronchopulmonary dysplasia (BPD) in preterm neonates ≤ 36 weeks' gestation

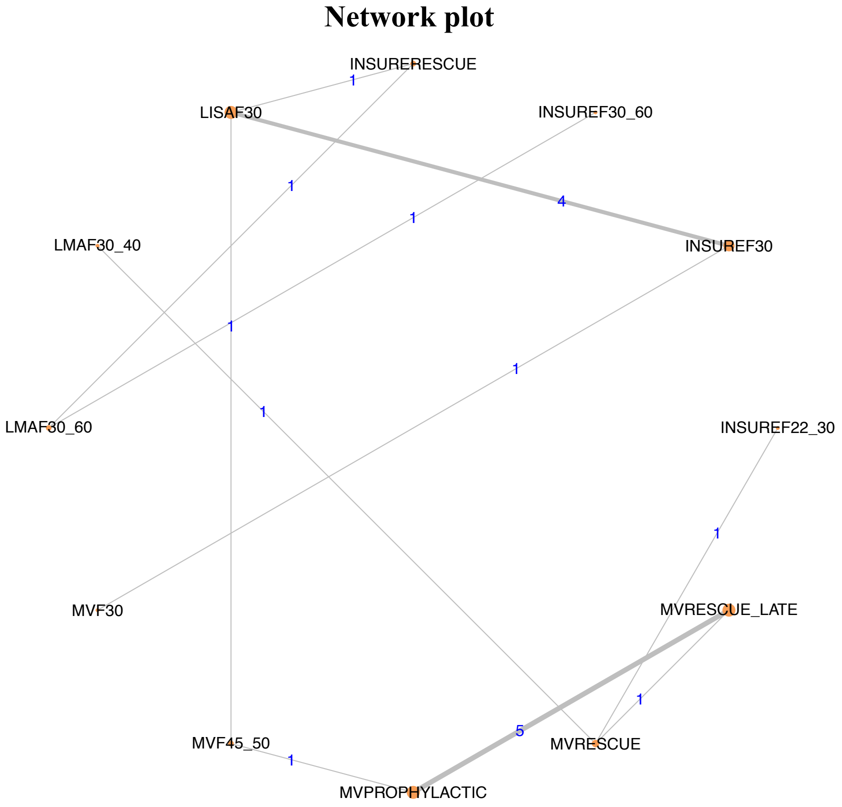

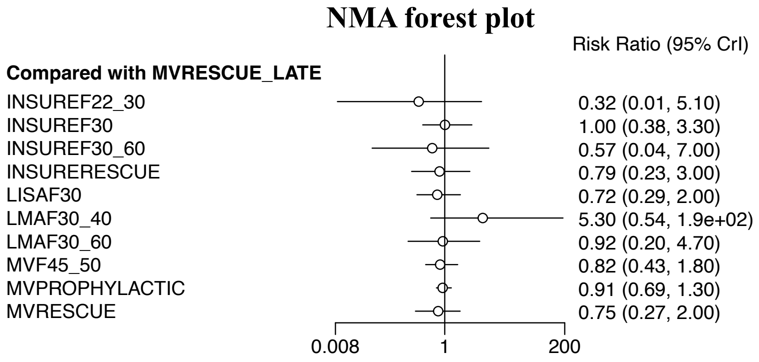

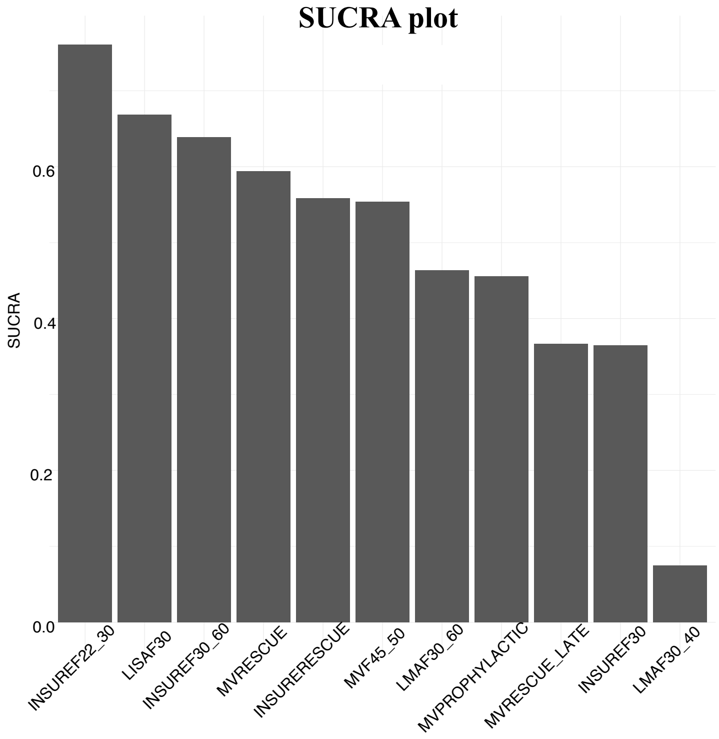

eFigure 15: Direct evidence from pairwise meta-analysis for the outcome of mortality or BPD in preterm neonates ≤ 36 weeks

Study	TE	seTE	Total	Total	RR	95%-CI
**MVRESCUE:MVRESCUE_LATE**						
Gortner 1998	−0.28	0.3985	154	163	0.76	[0.35; 1.65]
**INSURERESCUE:LISAF30**						
Gopel 2011	0.09	0.3276	112	108	1.09	[0.58; 2.08]
**MVF45_50:MVPROPHYLACTIC**						
SUPPORT 2010	−0.10	0.0538	663	653	0.90	[0.81; 1.00]
**MVPROPHYLACTIC:MVRESCUE_LATE**						
Merritt 1990	0.35	0.2372	76	72	1.42	[0.89; 2.26]
OSIRIS 1992	−0.18	0.0528	1344	1346	0.84	[0.75; 0.93]
Walti 1995	−0.28	0.1953	134	122	0.76	[0.51; 1.11]
Egberds 1993	−0.20	0.2857	75	72	0.82	[0.47; 1.43]
Dunn 1991	0.25	0.3362	62	60	1.29	[0.67; 2.49]
**Random effects model**			**1691**	**1672**	**0.92**	**[0.74; 1.14]**
Heterogeneity: $I^2 = 40\%$, $\tau^2 = 0.0230$, $p = 0.15$						
**INSUREF30:LISAF30**						
Gupta 2020	0.81	0.5405	29	29	2.25	[0.78; 6.49]
Kribs 2015	0.23	0.1813	104	107	1.26	[0.89; 1.80]
Bao 2015	−0.60	1.2064	43	47	0.55	[0.05; 5.81]
Mohammadizadeh 2015	0.56	0.5363	19	19	1.75	[0.61; 5.01]
**Random effects model**			**195**	**202**	**1.35**	**[0.98; 1.86]**
Heterogeneity: $I^2 = 0\%$, $\tau^2 = 0$, $p = 0.61$						
**INSUREF30_60:LMAF30_60**						
Pinheiro 2015	−0.41	0.8756	30	30	0.67	[0.12; 3.71]
**MVRESCUE:INSUREF22_30**						
Reininger 2005	0.67	1.2091	53	52	0.55	[0.05; 5.55]
**LISAF30:MVF45_50**						
Dargaville 2021	−0.13	0.0977	241	244	0.88	[0.73; 1.06]
**INSUREF30:MVF30**						
Dani 2004	−2.01	1.4497	13	14	0.13	[0.01; 2.31]
**LMAF30_40:MVRESCUE**						
Roberts 2017	1.67	1.0776	50	53	5.30	[0.64; 43.80]
**INSURERESCUE:LMAF30_60**						
Attridge 2012	−0.13	0.3377	13	13	0.87	[0.45; 1.70]

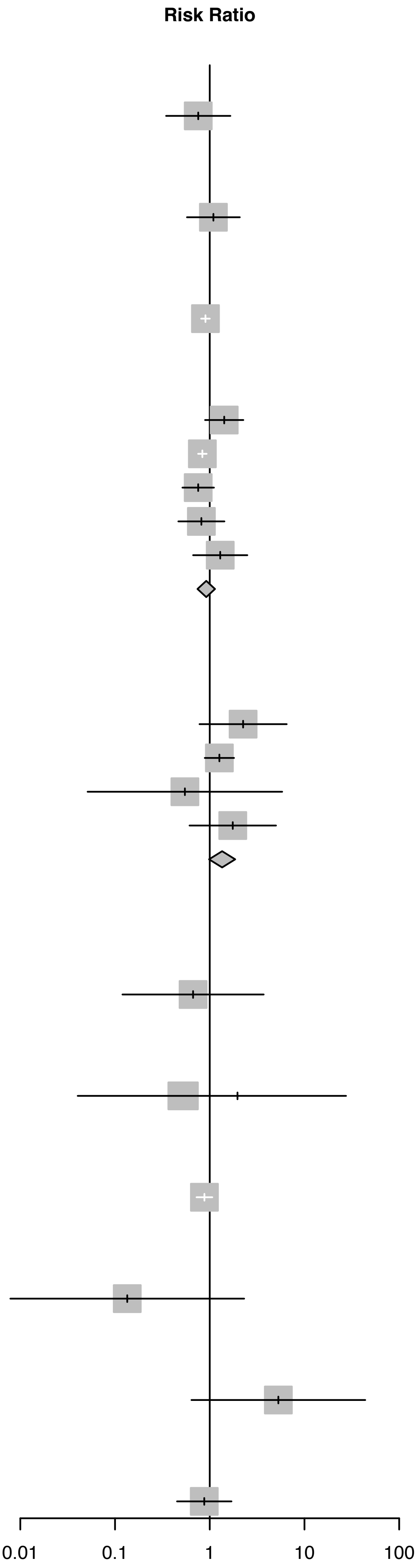

eFigure 16: Network plot, Forest plot depicting the network effect estimates and SUCRA plot for the outcome of Intraventricular Hemorrhage (IVH) > Grade 2 in preterm neonates ≤ 30 weeks' gestation

Network plot

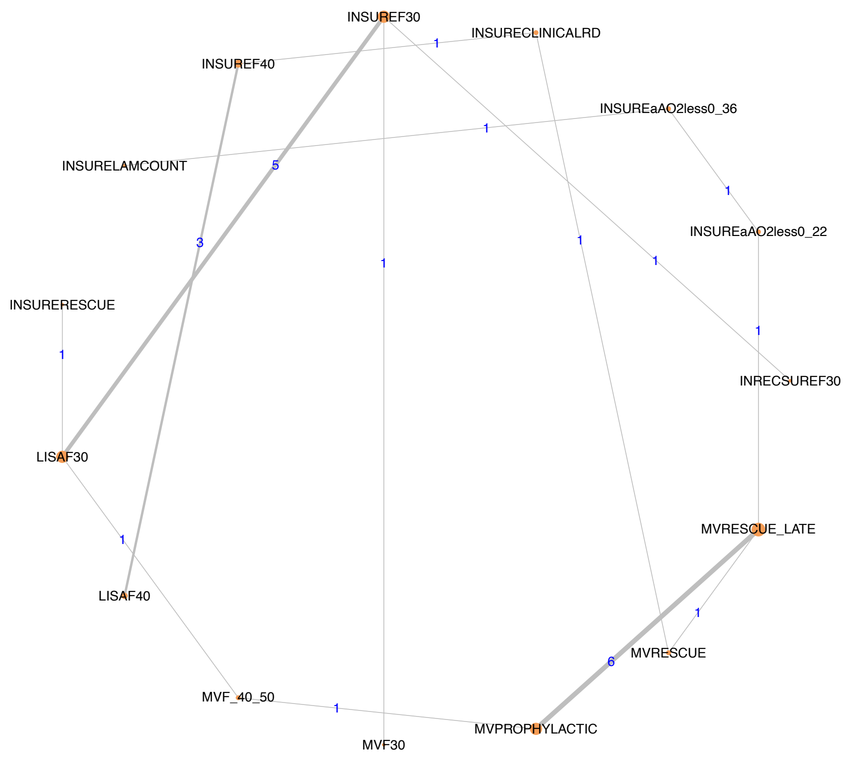

NMA forest plot

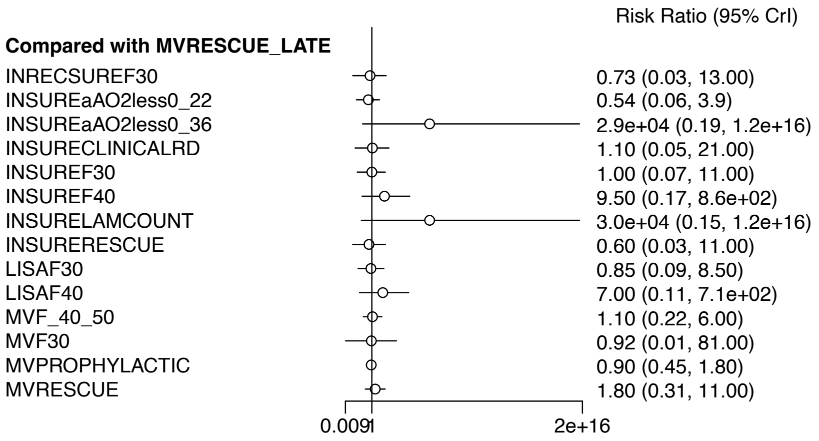

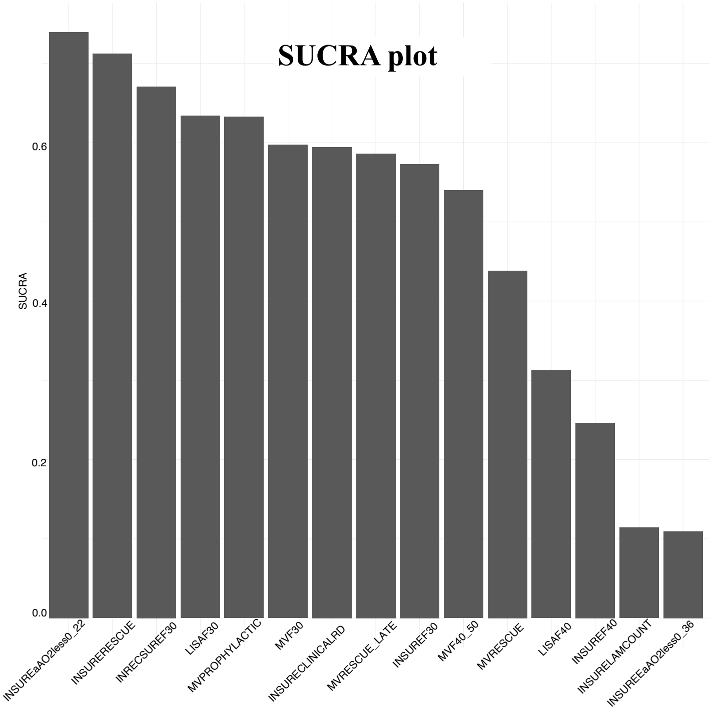

eFigure 17: Direct evidence from pairwise meta-analysis the outcome of IVH > Grade 2 in preterm neonates ≤ 30 weeks

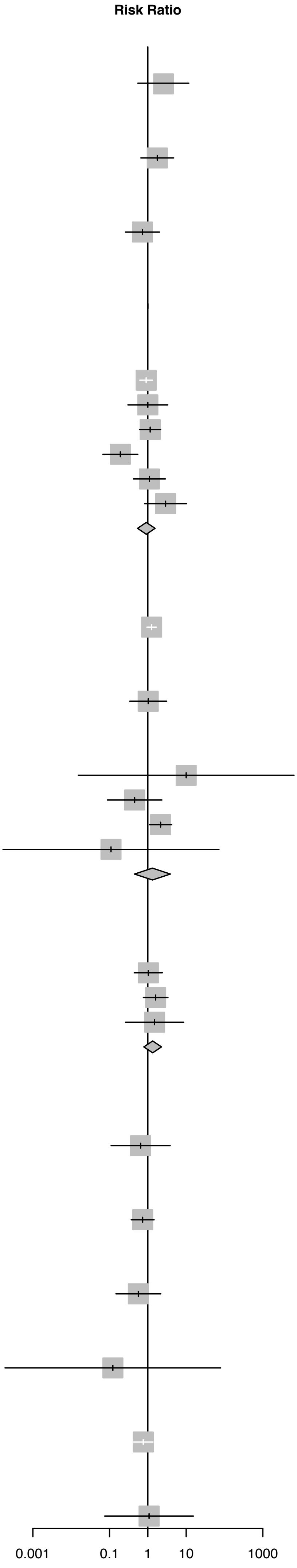

Study	TE	seTE	Experimental Total	Control Total	RR	95%-CI
**INSURECLINICALRD:INSUREF40**						
Dilmen 2013	−1.78	1.0684	79	80	5.88	[0.78; 50]
**MVRESCUE:MVRESCUE_LATE**						
Gortner 1998	0.57	0.5040	154	163	1.76	[0.66; 4.74]
**INSURERESCUE:LISAF30**						
Gopel 2011	−0.32	0.5230	112	108	0.72	[0.26; 2.02]
**MVPROPHYLACTIC:MVRESCUE_LATE**						
Kendig 1991	−0.10	0.1959	235	244	0.90	[0.61; 1.33]
Konishi 1992	0.00	0.6124	16	16	1.00	[0.30; 3.32]
Merritt 1990	0.14	0.3213	76	72	1.15	[0.61; 2.16]
Walti 1995	−1.65	0.5357	134	122	0.19	[0.07; 0.55]
Egberds 1993	0.09	0.4905	75	72	1.10	[0.42; 2.87]
Dunn 1991	1.07	0.6416	62	60	2.90	[0.83; 10.21]
Random effects model			**598**	**586**	**0.91**	**[0.54; 1.54]**
Heterogeneity: $I^2 = 60\%$, $\tau^2 = 0.2344$, $p = 0.03$						
**MVF_40_50:MVPROPHYLACTIC**						
SUPPORT 2010	0.23	0.1474	663	653	1.26	[0.94; 1.68]
**INSUREaAO2less_0_36:INSURELAMCOUNT**						
Verder 2013	0.02	0.5682	188	192	1.02	[0.34; 3.11]
**INSUREF30:LISAF30**						
Gupta 2020	2.30	3.3062	29	29	10.00	[0.02; 6520.07]
Heidarzadeh 2013	−0.79	0.8366	42	38	0.45	[0.09; 2.33]
Kribs 2015	0.77	0.3397	104	107	2.15	[1.11; 4.19]
Bao 2015	−2.21	3.3099	43	47	0.11	[0.00; 71.78]
Random effects model			**218**	**221**	**1.32**	**[0.45; 3.88]**
Heterogeneity: $I^2 = 24\%$, $\tau^2 = 0.3460$, $p = 0.27$						
**INSUREF40:LISAF40**						
Han 2020	0.03	0.4319	147	151	1.03	[0.44; 2.40]
Kanmaz 2013	0.47	0.3775	100	100	1.60	[0.76; 3.35]
Sabzehei 2022	0.41	0.8931	56	56	1.50	[0.26; 8.64]
Random effects model			**303**	**307**	**1.34**	**[0.79; 2.27]**
Heterogeneity: $I^2 = 0\%$, $\tau^2 = 0$, $p = 0.74$						
**INSURECLINICALRD:MVRESCUE**						
Rojas 2009	−0.43	0.9050	141	137	0.65	[0.11; 3.82]
**INRECSUREF30:INSUREF30**						
Vento 2020	−0.31	0.3519	107	111	0.73	[0.37; 1.46]
**INSUREaAO2less_0_22:MVRESCUE_LATE**						
Verder 1994	−0.57	0.6888	35	33	0.57	[0.15; 2.18]
**INSUREaAO2less_0_22:INSUREaAO2less_0_36**						
Verder 1999	−2.10	3.3065	27	33	0.12	[0.00; 79.73]
**LISAF30:MVF_40_50**						
Dargaville 2021	−0.28	0.2983	241	244	0.76	[0.42; 1.36]
**INSUREF30:MVF30**						
Dani 2004	0.07	1.3608	13	14	1.08	[0.07; 15.50]

eFigure 18: Metaregression at different gestational ages for the outcome of IVH > Grade 2 for the sub-group ≤ 30 weeks

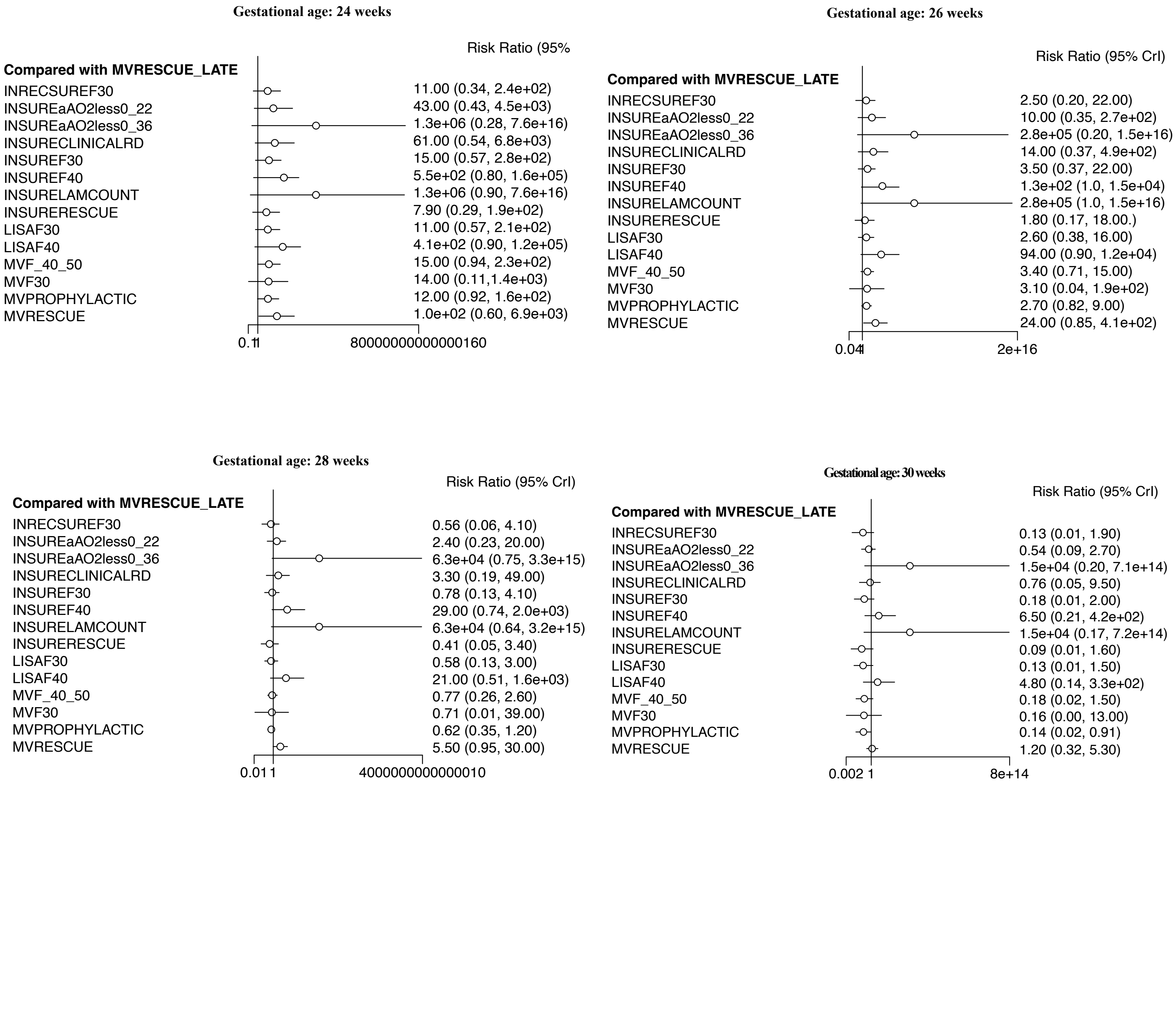

eFigure 19: Network plot, Forest plot depicting the network effect estimates and SUCRA plot for the outcome of IVH > Grade 2 in preterm neonates > 30 weeks' gestation

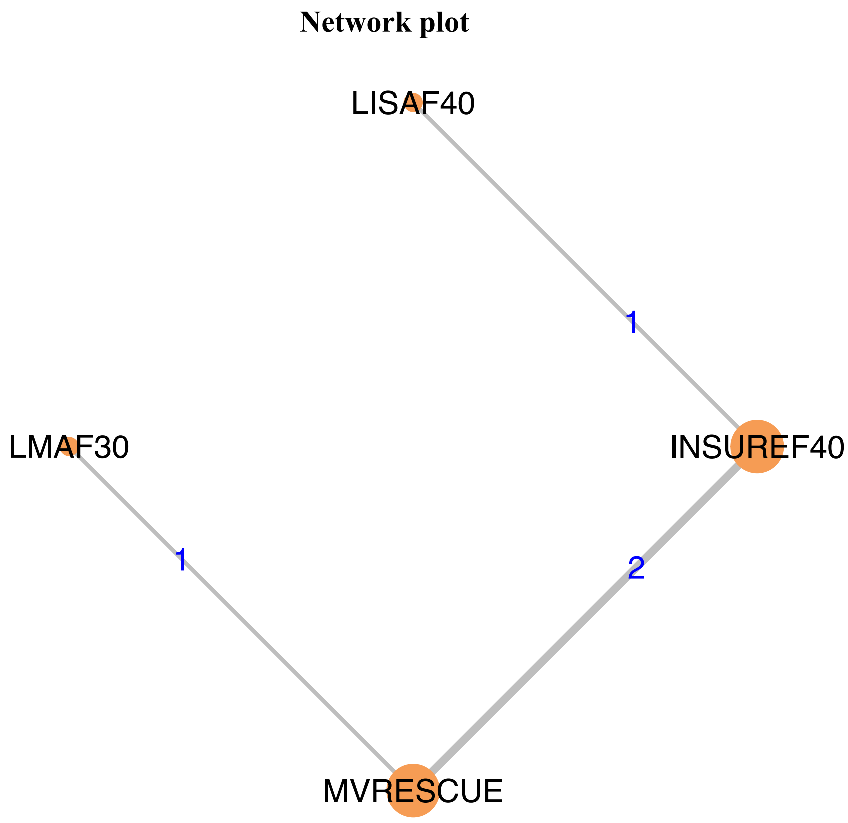

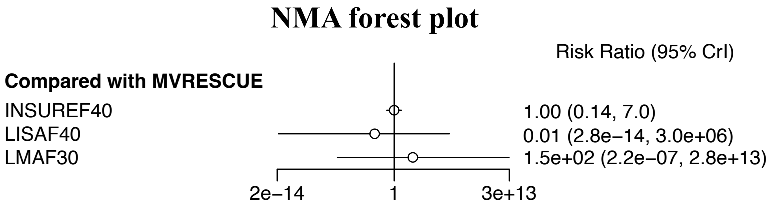

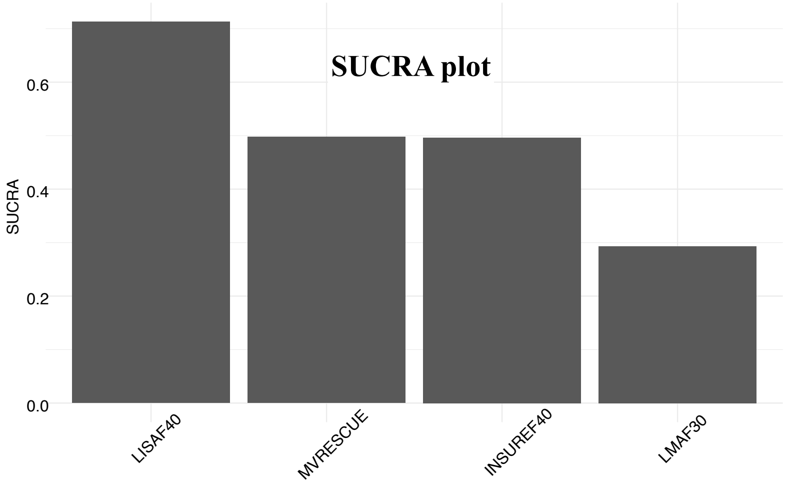

eFigure 20: Direct evidence from pairwise meta-analysis for the outcome of IVH > Grade 2 in preterm neonates > 30 weeks' gestation

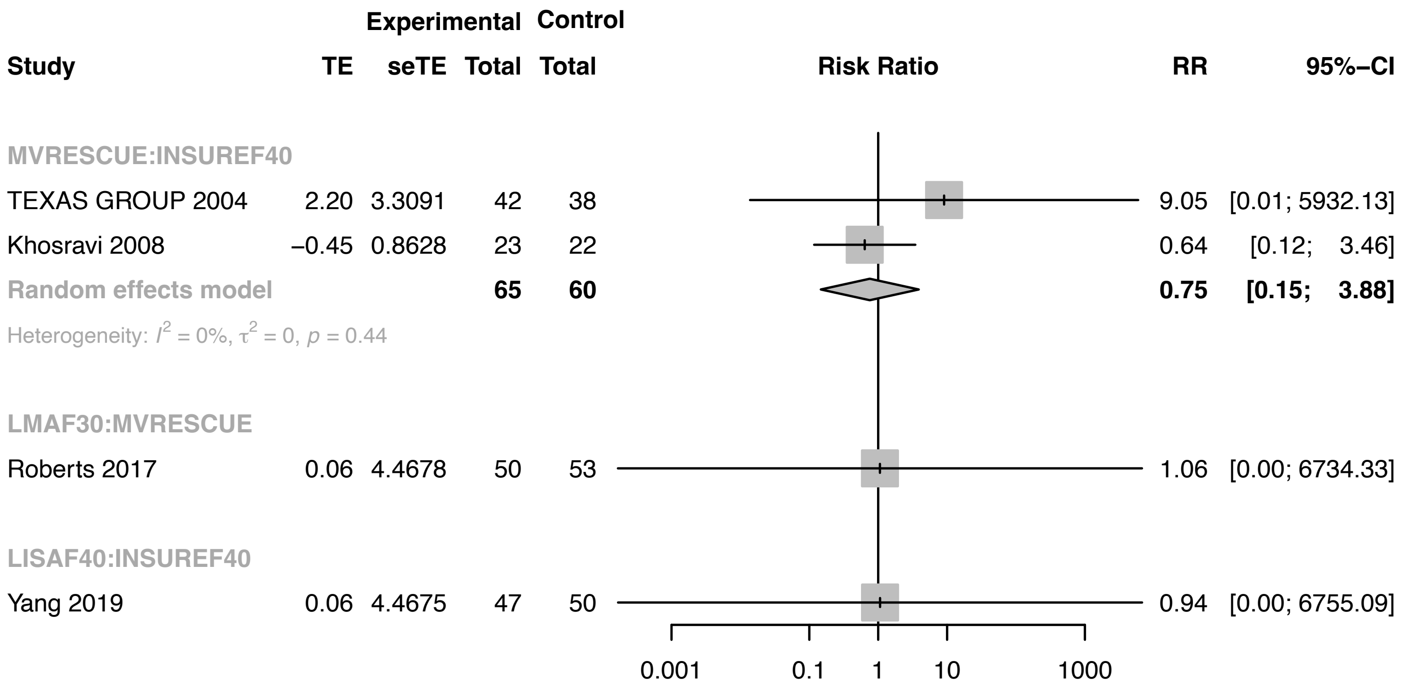

eFigure 21: Network plot, Forest plot depicting the network effect estimates and SUCRA plot for the outcome of air leak in preterm neonates ≤ 36 weeks' gestation

Network plot

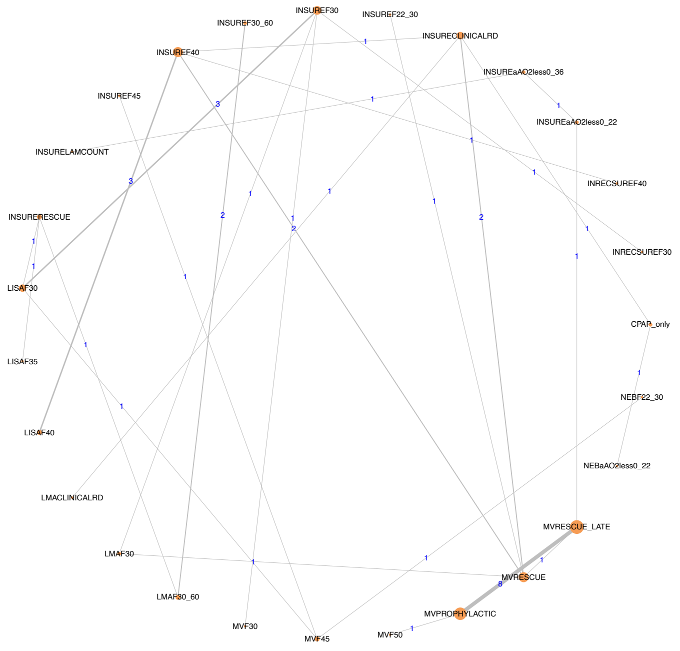

NMA Forest plot

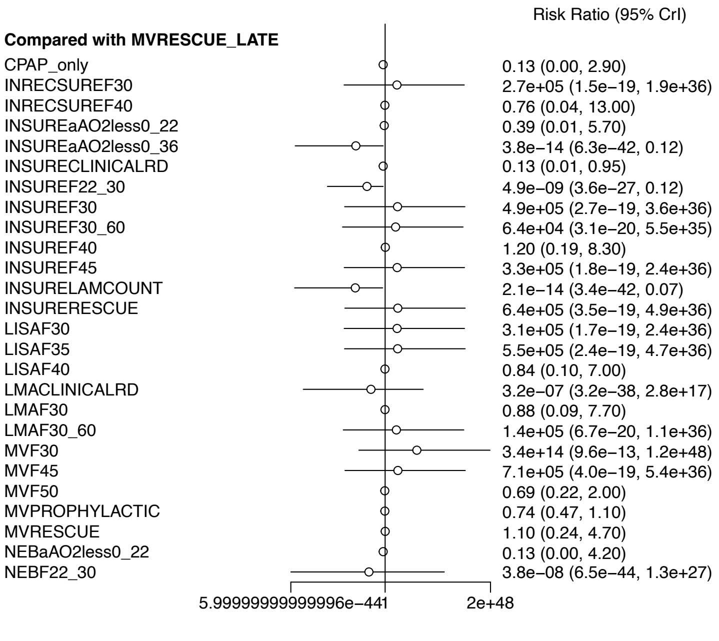

SUCRA plot

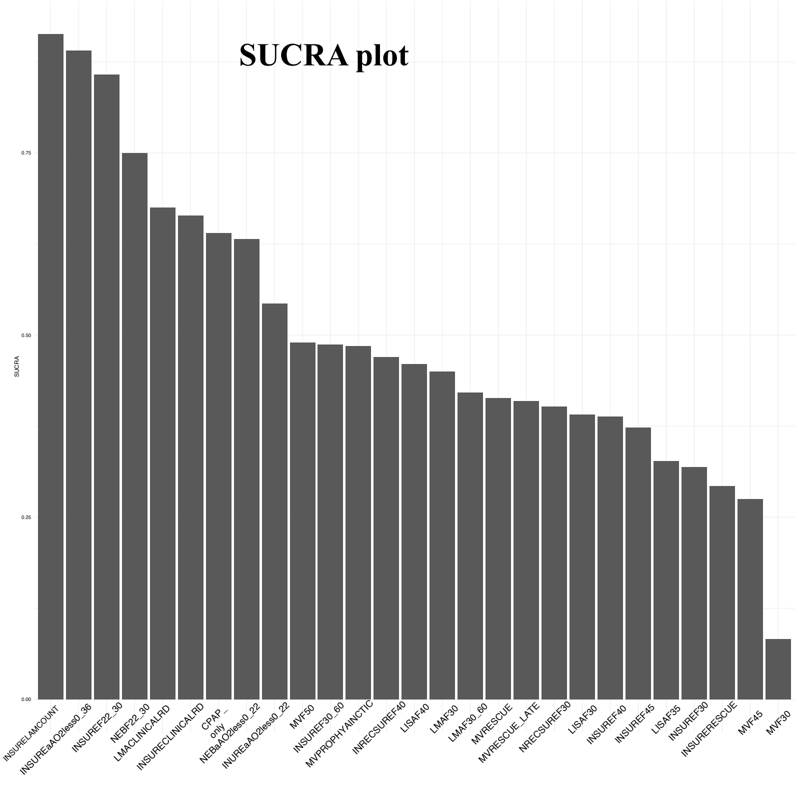

eFigure 22: Inconsistency assessment for the outcome of air leak

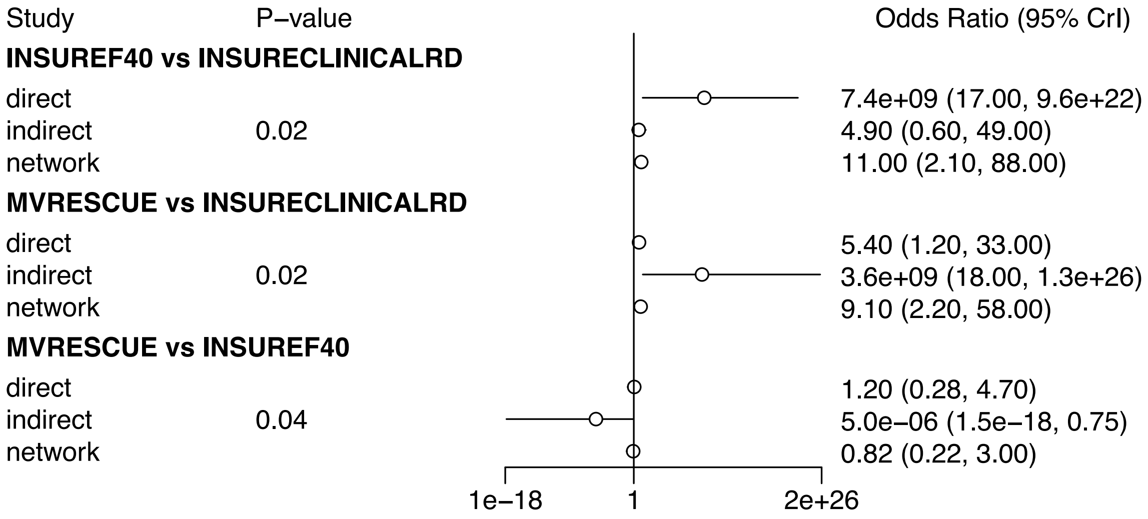

eFigure 23: Direct evidence from pairwise meta-analysis for the outcome of air leak in preterm neonates ≤ 36 weeks' gestation

Study	TE	seTE	Experimental Total	Control Total	RR	95%-CI
**INSURECLINICALRD:INSUREF40**						
Dilmen 2013	−4.24	3.1808	79	80	100	[0.13; -
**MVRESCUE:MVRESCUE_LATE**						
Gortner 1998	0.06	0.5663	154	163	1.06	[0.35; 3.21]
**INSURERESCUE:LISAF30**						
Gopel 2011	0.66	0.5973	112	108	1.93	[0.60; 6.22]
**INSURECLINICALRD:CPAP_only**						
Imani 2013	−0.00	0.9747	40	40	1.00	[0.15; 6.76]
**MVPROPHYLACTIC:MVRESCUE_LATE**						
Kendig 1991	−0.56	0.2977	235	244	0.57	[0.32; 1.03]
Konishi 1992	−1.39	1.0607	16	16	0.25	[0.03; 2.00]
Merritt 1990	0.53	0.2252	76	72	1.71	[1.10; 2.65]
OSIRIS 1992	−0.32	0.0674	1344	1346	0.73	[0.64; 0.83]
Walti 1995	−0.55	0.2652	134	122	0.58	[0.34; 0.97]
Dunn 1991	−0.73	0.5169	62	60	0.48	[0.18; 1.33]
Lefort 2003	−0.16	0.4019	34	40	0.86	[0.39; 1.88]
Random effects model			**1901**	**1900**	**0.76**	**[0.55; 1.04]**
Heterogeneity: $I^2 = 59\%$, $\tau^2 = 0.1007$, $p = 0.02$						
**INSURERESCUE:LISAF35**						
Olivier 2017	0.13	1.3823	21	24	1.14	[0.08; 17.16]
**MVF50:MVPROPHYLACTIC**						
SUPPORT 2010	−0.08	0.2000	663	652	0.92	[0.62; 1.36]
**INSUREF40:MVRESCUE**						
TEXAS GROUP 2004	0.03	0.7977	65	67	1.03	[0.22; 4.92]
Khosravi 2008	−0.24	0.7031	22	23	0.78	[0.20; 3.11]
Random effects model			**87**	**90**	**0.88**	**[0.31; 2.48]**
Heterogeneity: $I^2 = 0\%$, $\tau^2 = 0$, $p = 0.80$						
**INSUREaAO2less_0_36:INSURELAMCOUNT**						
Verder 2013	0.53	0.7231	188	192	1.70	[0.41; 7.02]
**INSUREF30_60:LMAF30_60**						
Amini 2019	−3.00	3.2301	30	30	0.05	[0.00; 28.08]
Pinheiro 2015	−0.41	0.5916	30	30	0.67	[0.21; 2.13]
Random effects model			**60**	**60**	**0.61**	**[0.20; 1.92]**
Heterogeneity: $I^2 = 0\%$, $\tau^2 = 0$, $p = 0.43$						
**NEBaAO2less0_22:CPAP_only**						
Berggren 2000	0.00	0.6124	16	16	1.00	[0.30; 3.32]
**INSUREF30:LISAF30**						
Heidarzadeh 2013	−0.10	0.9746	42	38	0.90	[0.13; 6.11]
Kribs 2015	0.98	0.5079	104	107	2.67	[0.99; 7.24]
Bao 2015	−0.20	0.7340	43	47	0.82	[0.19; 3.46]
Random effects model			**189**	**192**	**1.59**	**[0.71; 3.55]**
Heterogeneity: $I^2 = 9\%$, $\tau^2 = 0.0494$, $p = 0.33$						
**INSUREF40:LISAF40**						
Kanmaz 2013	0.37	0.4099	100	100	1.44	[0.65; 3.23]
Sabzehei 2022	0.41	0.8931	56	56	1.50	[0.26; 8.64]
Yang 2019	3.34	3.2081	50	47	28.20	[0.05; 15170.81]
Random effects model			**206**	**203**	**1.51**	**[0.73; 3.12]**
Heterogeneity: $I^2 = 0\%$, $\tau^2 = 0$, $p = 0.66$						
**NEBF22_30:MVF45**						
Minocchieri 2019	3.00	3.2307	32	32	20.00	[0.04; 11246.49]
**INSUREF22_30:MVRESCUE**						
Reininger 2005	−3.67	3.1956	52	53	0.03	[0.00; 13.38]
**INSURECLINICALRD:MVRESCUE**						
Rojas 2009	−1.42	0.6343	141	137	0.24	[0.07; 0.84]
Xi-Lin 2013	0.00	1.3919	32	32	1.00	[0.07; 15.30]
Random effects model			**173**	**169**	**0.31**	**[0.10; 0.96]**
Heterogeneity: $I^2 = 0\%$, $\tau^2 = 0$, $p = 0.35$						
**INSUREF30:INRECSUREF30**						
Vento 2020	−0.59	0.4163	111	107	1.81	[0.80; 4.17]
**INSUREaAO2less_0_22:MVRESCUE_LATE**						
Verder 1994	−0.75	1.2005	35	33	0.47	[0.04; 4.96]
**INSUREaAO2less_0_22:INSUREaAO2less_0_36**						
Verder 1999	2.50	3.3065	27	33	12.22	[0.02; 7972.82]
**INRECSUREF40:INSUREF40**						
Yang 2022	−0.41	0.9009	92	92	0.67	[0.11; 3.90]
**LISAF30:MVF45**						
Dargaville 2021	−0.81	0.3502	241	244	0.45	[0.22; 0.88]
**INSUREF30:MVF30**						
Dani 2004	−2.23	3.2942	13	14	0.11	[0.00; 68.58]
**LMAF30:MVRESCUE**						
Roberts 2017	−0.16	0.6412	50	53	0.85	[0.24; 2.98]
**INSUREF45:MVF45**						
Nayeri 2014	−0.69	0.6362	21	21	0.50	[0.14; 1.74]
**INSUREF30:LMAF30**						
Sadeghnia 2014	−0.00	4.4657	35	35	1.00	[0.00; 6327.72]
**INSURECLINICALRD:LMACLINICALRD**						
Garehbaghi 2018	−0.00	4.4632	25	25	1.00	[0.00; 6296.05]

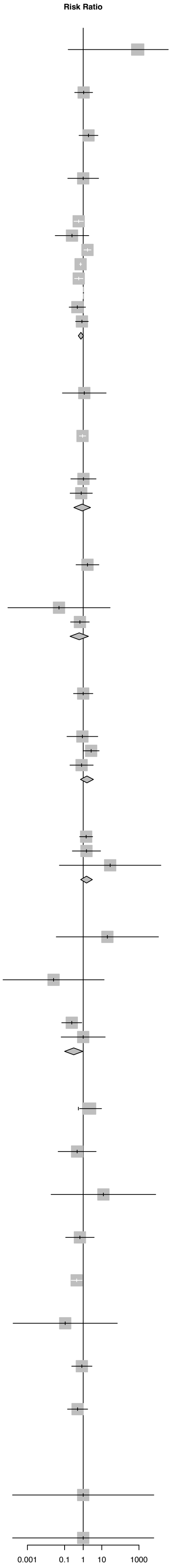

eFigure 24: Metaregression at different gestational ages for the outcome of air leak in preterm neonates ≤ 36 weeks' gestation

Gestational age: 28 weeks

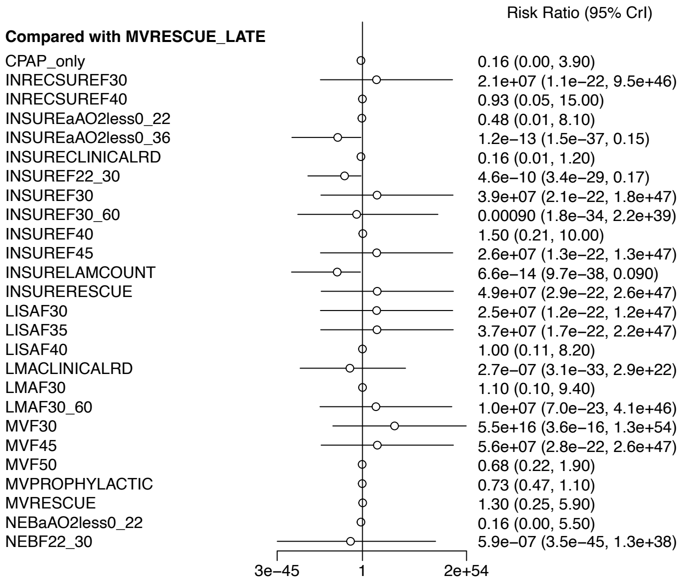

Gestational age: 32 weeks

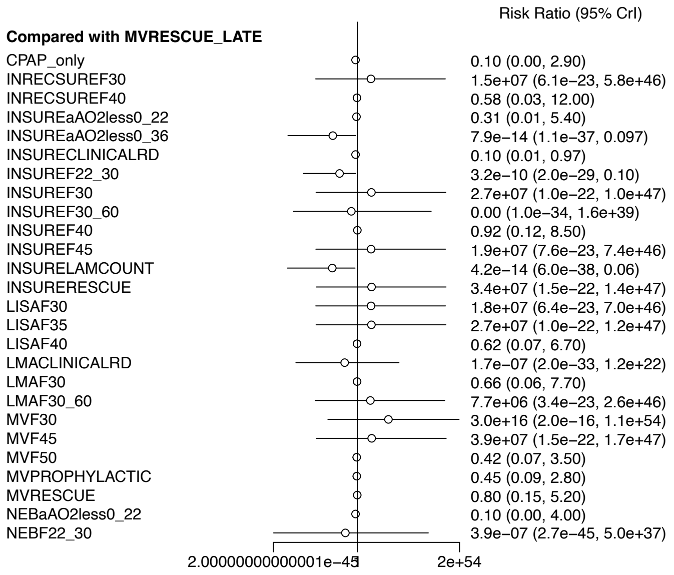

Gestational age: 36 weeks

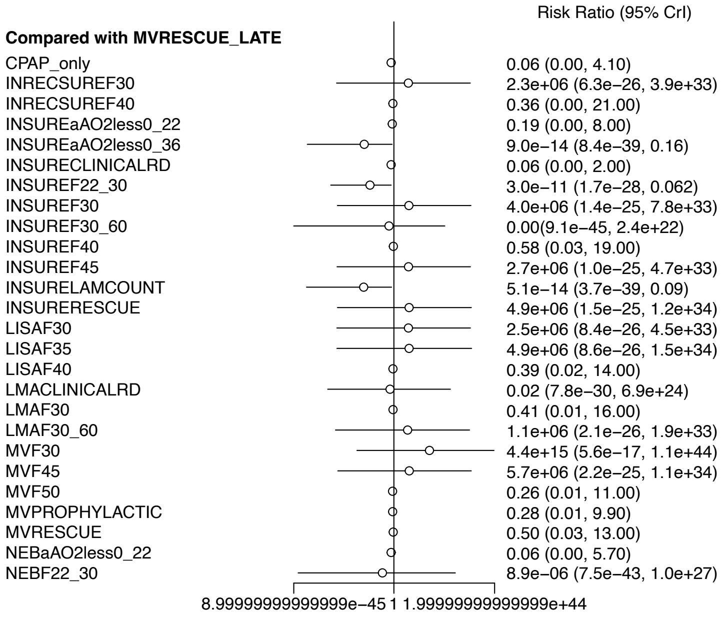

eFigure 25: Network plot, Forest plot depicting the network effect estimates and SUCRA plot for the outcome of receipt of multiple doses of surfactant in preterm neonates ≤ 36 weeks' gestation

Network plot

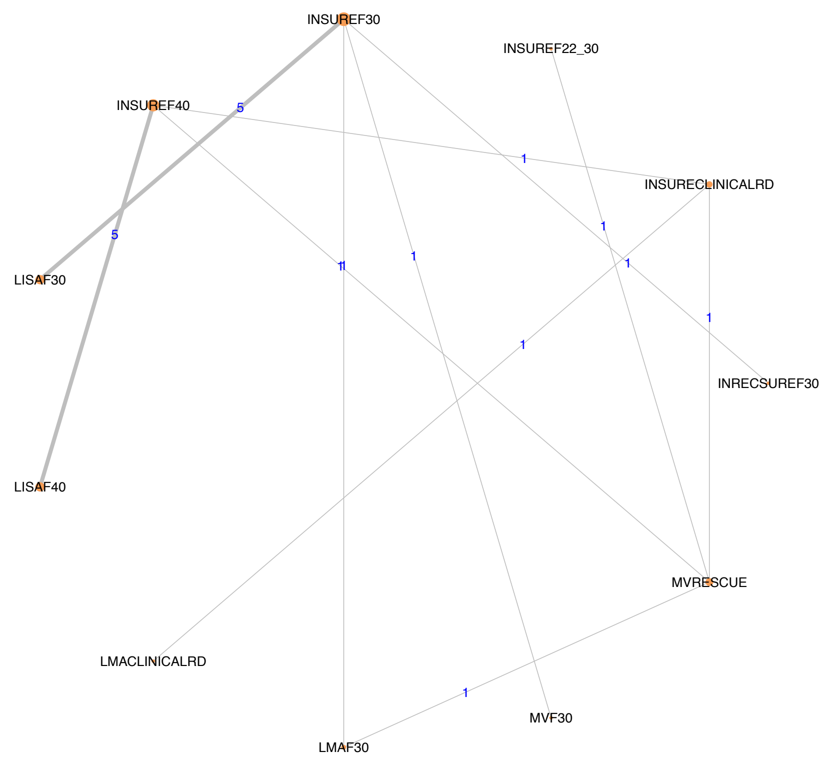

NMA forest plot

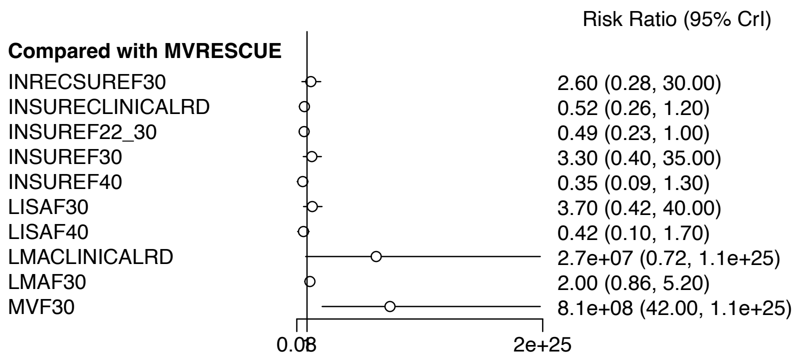

SUCRA plot

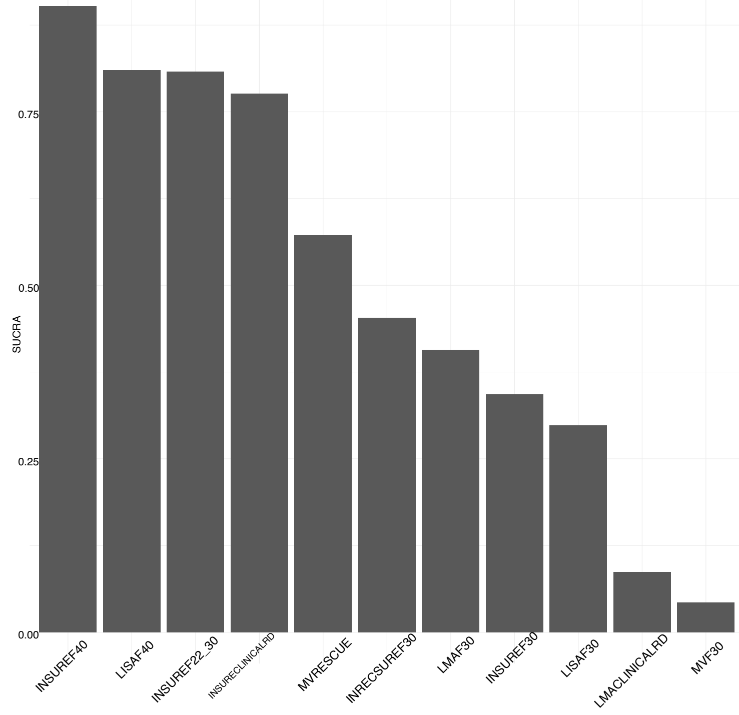

eFigure 26: Inconsistency assessment for the outcome of receipt of multiple doses of surfactant in preterm neonates ≤ 36 weeks' gestation

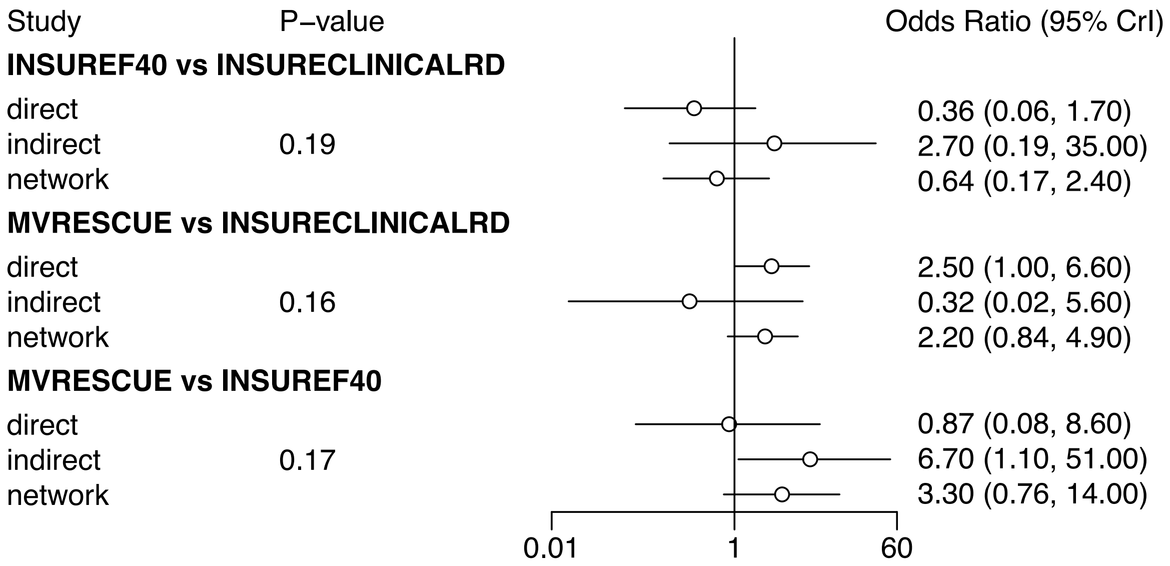

eFigure 27: Direct evidence from pairwise meta-analysis for the outcome of receipt of multiple doses of surfactant in preterm neonates ≤ 36 weeks' gestation

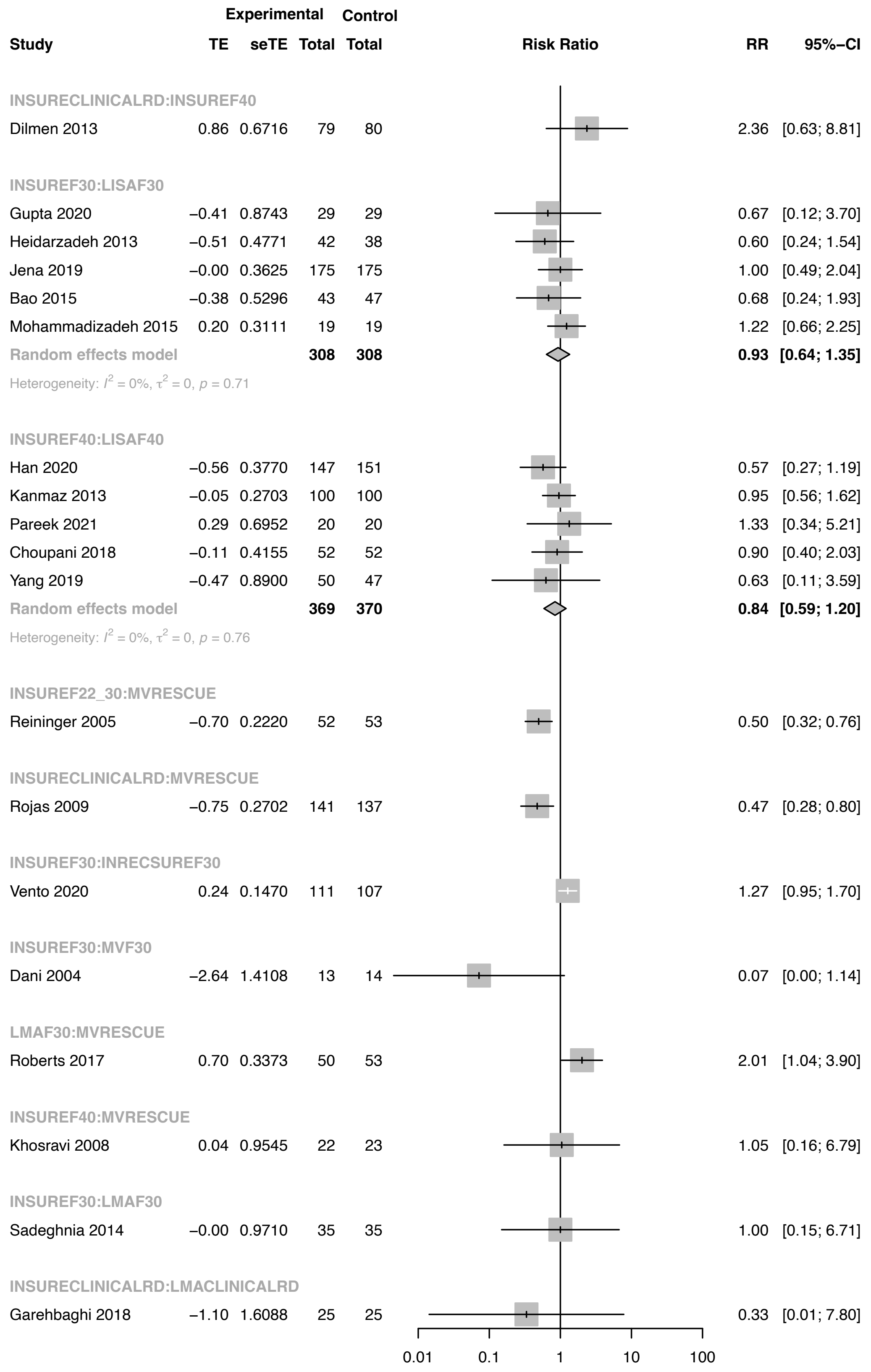

eFigure 28: Metaregression at different gestational ages for the outcome of receipt of multiple doses of surfactant in preterm neonates ≤ 36 weeks' gestation

Gestational age: 28 weeks

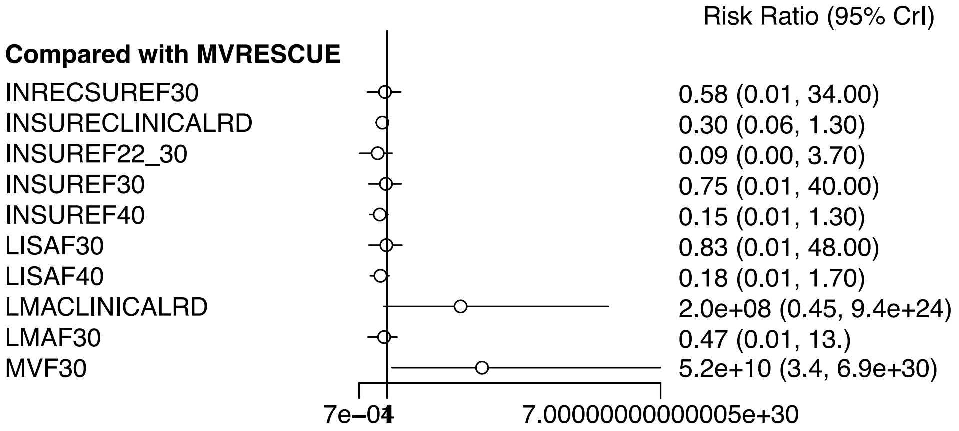

Gestational age: 32 weeks

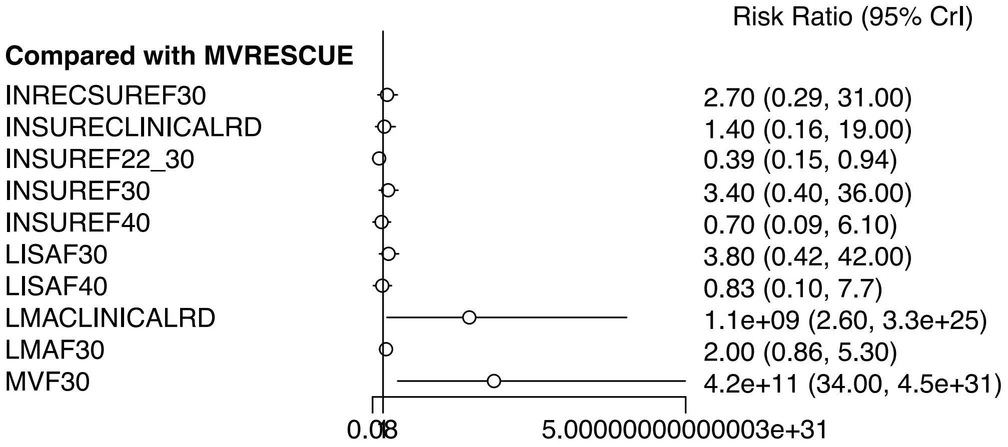

Gestational age: 36 weeks

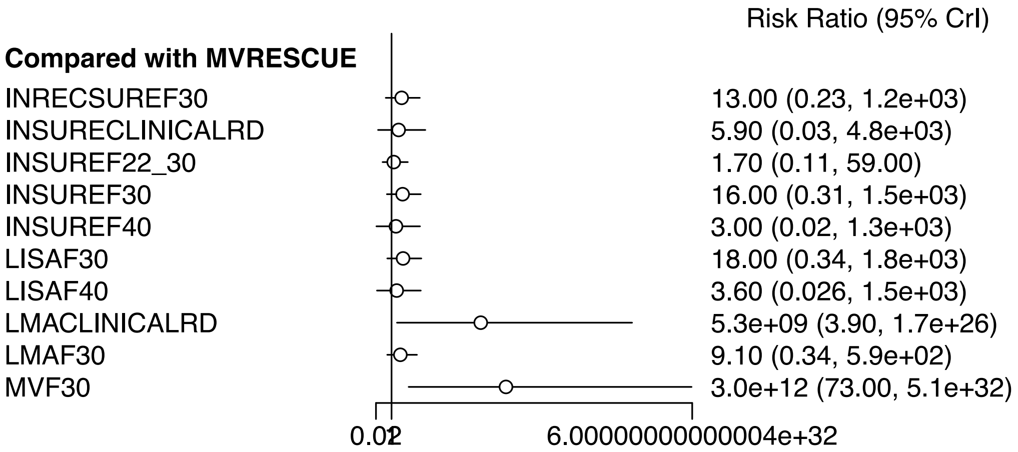

eTable 1: Literature search strategy

Database(s): Ovid MEDLINE(R) ALL 1946 to August 12, 2022<br>The initial search was dated Feb 10, 2022, and was later updated.		
#	Searches	Results
1	exp Pulmonary Surfactants/	13883
2	exp Pulmonary Alveoli/	27265
3	exp Surface-Active Agents/	138971
4	(surfactant or surfactants).ab,ti.	65735
5	1 or 2 or 3 or 4	211291
6	exp respiratory distress syndrome, newborn/ or exp hyaline membrane disease/ or exp "transient tachypnea of the newborn"/	15802
7	("Respiratory Distress" or RDS or "Pulmonary Inflammations" or "Pulmonary Inflammation" or "Lung Inflammation" or "lung inflammations" or "Hyaline Membrane Disease" or "Hyaline Membrane Diseases" or HMD).ab,ti.	67367
8	6 or 7	74669
9	infant, newborn/ or infant, low birth weight/ or infant, small for gestational age/ or infant, very low birth weight/ or infant, premature/ or infant, extremely premature/	657934
10	(Neonate* or Newborn* or Preterm* or term or prematur* or "Low birth weight" or lbw or vlbw or "Low birth weights" or "Low birthweight" or "Low birthweights" or "pre-terms" or "Pre-term" or "Small gestational age" or SGA or "Extremely premature").ab,ti.	1694919
11	9 or 10	2093431
12	5 and 8 and 11	4683
13	randomized controlled trial.pt.	574945
14	controlled clinical trial.pt.	94985
15	(random* adj3 (group* or control* or trial* or placebo)).ab,ti.	574053
16	13 or 14 or 15	982229
17	exp animals/ not humans.sh.	5036395
18	16 not 17	949160
19	12 and 18	779
20	limit 19 to dt="20220201-20230101"	12

Database(s): Embase Classic+Embase 1947 to 2022 August 12<br>The initial search was dated Feb 10, 2022, and was later updated.		
#	Searches	Results
1	exp lung surfactant/ or exp respiratory tract agent/ or exp artificial lung surfactant/ or exp lung surfactant extract/	1219990
2	(surfactant or surfactants).ab,ti.	81032
3	1 or 2	1285975
4	neonatal pneumothorax/ or neonatal respiratory distress syndrome/ or exp respiratory distress syndrome/ or exp neonatal respiratory failure/	96784
5	exp hyaline membrane disease/ or exp lung malformation/ or exp respiratory distress syndrome/	117085
6	("Respiratory Distress" or RDS or "Pulmonary Inflammations" or "Pulmonary Inflammation" or "Lung Inflammation" or "lung inflammations" or "Hyaline Membrane Disease" or "Hyaline Membrane Diseases" or HMD).ab,ti.	98263
7	4 or 5 or 6	170203
8	exp prematurity/	131398
9	(Preterm* or term or prematur* or "Low birth weight" or lbw or vlbw or "Low birth weights" or "Low birthweight" or "Low birthweights" or "pre-terms" or "Pre-term" or "Small gestational age" or SGA or "Extremely premature").ab,ti.	2099181
10	8 or 9	2121769
11	3 and 7 and 10	8033
12	randomized controlled trial.mp. or exp randomized controlled trial/	972494
13	controlled clinical trial.mp. or exp controlled clinical trial/	933354
14	(random* adj3 (group* or control* or trial* or placebo)).ab,ti.	799513
15	12 or 13 or 14	1450767
16	11 and 15	1455
17	limit 16 to dc="20220201-20230101"	48

Cochrane Library, February 11, 2022		
ID	Search	Hits
#1	MeSH descriptor: [Pulmonary Surfactants] explode all trees	562
#2	("surfactant" OR surfactants):ti	1137
#3	("surfactant" OR surfactants):ab	1746
#4	{OR #1-#3}	2150
#5	MeSH descriptor: [Infant, Newborn] explode all trees	17137
#6	(neonat* or newborn* or Preterm* or premature or "Low birth weight" or lbw or vlbw or "Low birth weights" or "Low birthweight" or "Low birthweights" or "pre-terms" or "Pre-term" or "Small gestational age" or SGA or "Extremely premature"):ti,ab	47312
#7	{OR #5-#6}	53287
#8	MeSH descriptor: [Respiratory Distress Syndrome, Newborn] 3 tree(s) exploded	1743
#9	("Respiratory Distress" OR RDS OR "Pulmonary Inflammations" OR "Pulmonary Inflammation" OR "Lung Inflammation" OR "lung inflammations" OR "Hyaline Membrane Disease" OR "Hyaline Membrane Diseases"):ab,ti	6494
#10	{OR #8-#9}	7062
#11	#4 AND #7 AND #10 (1005 Trials and 45 reviews)	1050

CINAHL, February 10, 2022 (Ebsco)		
#	Query	Results
S15	S11 AND S14	317
S14	S12 OR S13	4,45,487
S13	TI ((random* N3 (group* or control* or trial* or placebo)) OR AB ((random* N3 (group* or control* or trial* or placebo))	2,51,528
S12	(MH "Clinical Trials+") OR (MH "Randomized Controlled Trials+")	3,32,357
S11	S3 AND S6 AND S10	1,110
S10	S7 OR S8 OR S9	16,553
S9	TI (("Respiratory Distress" OR RDS OR "Pulmonary Inflammations" OR "Pulmonary Inflammation" OR "Lung Inflammation" OR "lung inflammations" OR "Hyaline Membrane Disease" OR "Hyaline Membrane Diseases")) OR AB (("Respiratory Distress" OR RDS OR "Pulmonary Inflammations" OR "Pulmonary Inflammation" OR "Lung Inflammation" OR "lung inflammations" OR "Hyaline Membrane Disease" OR "Hyaline Membrane Diseases"))	14,812
S8	(MH "Hyaline Membrane Disease")	141
S7	(MH "Respiratory Distress Syndrome+")	3,653
S6	S4 OR S5	2,34,782
S5	TI ((neonat* or newborn* or Preterm* or premature or "Low birth weight" or lbw or vlbw or "Low birth weights" or "Low birthweight" or "Low birthweights" or "pre-terms" or "Pre-term" or "Small gestational age" or SGA or "Extremely premature")) OR AB ((neonat* or newborn* or Preterm* or premature or "Low birth weight" or lbw or vlbw or "Low birth weights" or "Low birthweight" or "Low birthweights" or "pre-terms" or "Pre-term" or "Small gestational age" or SGA or "Extremely premature"))	1,44,009
S4	(MH "Infant, Newborn, Diseases+") OR (MH "Infant, Newborn+") OR (MH "Infant, High Risk") OR (MH "Infant, Premature") OR (MH "Infant, Very Low Birth Weight")	1,67,246
S3	S1 OR S2	3,395
S2	TI surfactant OR AB surfactant	3,003
S1	(MH "Pulmonary Surfactants")	1,648

*The literature search was updated until 16 May 2023 for all the databases. No additional eligible trials were identified.

eTable 2: Risk of bias assessment of the included studies

Study ID	Randomization process	Deviation from intended intervention	Missing outcome data	Measurement of outcome	Selection of reported results	Overall risk of bias
Amini 2019	Low risk	Low risk	Low risk	Low risk	Low risk	Low risk
Attridge 2012	Low risk	Low risk	Low risk	Low risk	Some concerns	Some concerns
Bao 2015	Low risk	Low risk	Low risk	Low risk	Low risk	Low risk
Barbosa 2017	Low risk	Low risk	Low risk	Low risk	Low risk	Low risk
Berggren 2000	Low risk	Low risk	Low risk	Low risk	Some concerns	Some concerns
Boskabadi 2019	Some concerns	Low risk	Low risk	Low risk	Some concerns	High risk
Choupani 2018	Some concerns	Low risk	Low risk	Low risk	Some concerns	High risk
Dani 2004	Low risk	Low risk	Low risk	Low risk	Some concerns	Some concerns
Dargaville 2021	Low risk	Low risk	Low risk	Low risk	Low risk	Low risk
Dilmen 2013	Low risk	Low risk	Low risk	Low risk	Some concerns	Some concerns
Dunn 1990	Low risk	Low risk	Low risk	Low risk	Some concerns	Some concerns
Egberts 1993	Low risk	Low risk	Low risk	Low risk	Some concerns	Some concerns
Gharehbaghi 2018	Low risk	Low risk	Low risk	Low risk	Low risk	Low risk
Gopel 2011, Herting 2020	Low risk	Low risk	Low risk	Low risk	Low risk	Low risk
Gortner 1998, Hentschel 2009	Low risk	Low risk	Low risk	Low risk	Some concerns	Some concerns
Gupta 2020	Low risk	Low risk	Low risk	Low risk	Low risk	Low risk
Gallup 2021	NA	NA	NA	NA	NA	NA
Han 2020	Low risk	Low risk	Low risk	Some concerns	Low risk	Some concerns
Heidarzadeh 2013	Some concerns	Low risk	Low risk	Low risk	Some concerns	High risk
Huang 2013	Some concerns	Low risk	Low risk	Low risk	Some concerns	High risk
Imani 2013	Some concerns	Low risk	Low risk	Low risk	Some concerns	High risk
Jena 2019	Low risk	Low risk	Low risk	Low risk	Low risk	Low risk
Kandraju 2013	Low risk	Low risk	Low risk	Low risk	Low risk	Low risk
Kanmaz 2013	Low risk	Low risk	Low risk	Low risk	Low risk	Low risk
Kendig 1991	Low risk	Low risk	Low risk	Low risk	Some concerns	Some concerns
Khosravi 2008	Some concerns	Low risk	Low risk	Low risk	Some concerns	High risk
Konishi 1992	Some concerns	Low risk	Low risk	Low risk	Some concerns	High risk
Kribs 2015, Mehler 2020	Low risk	Low risk	Low risk	Low risk	Low risk	Low risk
Lefort 2003	Low risk	Low risk	Low risk	Low risk	Some concerns	Some concerns
Li 2016	Some concerns	Low risk	Low risk	Low risk	Some concerns	High risk
Merritt 1991, Vaucher 1993	Low risk	Low risk	Low risk	Low risk	Some concerns	Some concerns
Minocchieri 2019	Low risk	Low risk	Low risk	Low risk	Low risk	Low risk
Mohammadizadeh 2014	Some concerns	Low risk	Low risk	Low risk	Some concerns	High risk
Tapia 2012	Low risk	Low risk	Low risk	Low risk	Low risk	Low risk
Nayeri 2014	Some concerns	Low risk	Low risk	Low risk	Some concerns	High risk
Olivier 2017	Low risk	Low risk	Low risk	Low risk	Some concerns	Some concerns
OSIRIS 1992	Low risk	Low risk	Low risk	Low risk	Some concerns	Some concerns
Pareek 2021	Low risk	Low risk	Low risk	Low risk	Low risk	Low risk
Pinheiro 2016	Some concerns	Low risk	Low risk	Low risk	Some concerns	High risk
Reininger 2005	Low risk	Low risk	Low risk	Low risk	Some concerns	Some concerns
Roberts 2017	Low risk	Low risk	Low risk	Low risk	Low risk	Low risk
Rodriguez-Fanjul 2020	Low risk	Low risk	Low risk	Low risk	Low risk	Low risk
Rojas 2009	Low risk	Low risk	Low risk	Low risk	Low risk	Low risk
Sabzehei 2022	Low risk	Low risk	Low risk	Low risk	Low risk	Low risk
Sadeghnia 2014	Some concerns	Low risk	Low risk	Low risk	Some concerns	High risk
SUPPORT 2010	Low risk	Low risk	Low risk	Low risk	Low risk	Low risk
Texas 2004	Low risk	Low risk	Low risk	Low risk	Some concerns	Some concerns
Vento 2020	Low risk	Low risk	Low risk	Low risk	Low risk	Low risk
Verder 1994	Low risk	Low risk	Low risk	Low risk	Some concerns	Some concerns
Verder 1999	Low risk	Low risk	Low risk	Low risk	Some concerns	Some concerns
Verder 2013	Low risk	Low risk	Low risk	Low risk	Some concerns	Some concerns
Walti 1995	Low risk	Low risk	Low risk	Low risk	Some concerns	Some concerns
Yang 2019	Some concerns	Low risk	Low risk	Low risk	Some concerns	High risk
Yang 2022	Low risk	Low risk	Low risk	Low risk	Low risk	Low risk

eTable 3: Network characteristics for the outcome of requirement of IMV for the sub-group ≤30 weeks

comparison	n.studies	n.patients	n.outcomes	proportion
CPAP_only vs. INSURECLINICALRD	1	80	10	0.1250000
INRECSURE_F30 vs. INSUREF30	1	218	103	0.4724771
INRECSURE_RESCUE vs. INSURERESCUE	1	184	56	0.3043478
INSUREaAO2less0_22 vs. INSUREaAO2less0_36	1	60	25	0.4166667
INSUREaAO2less0_22 vs. MVRESCUE_LATE	1	68	43	0.6323529
INSUREaAO2less0_36 vs. INSURELAMCOUNT	1	380	129	0.3394737
INSURECLINICALRD vs. INSUREF40	1	159	39	0.2452830
INSURECLINICALRD vs. INSUREF45_50	1	153	37	0.2418301
INSURECLINICALRD vs. MVRESCUE	1	278	90	0.3237410
INSUREF30 vs. LISAF30	5	477	228	0.4779874
INSUREF40 vs. LISAF40	3	352	134	0.3806818
INSUREF45_50 vs. MVF45	1	42	19	0.4523810
INSURERESCUE vs. LISAF30	1	220	118	0.5363636
LISA_LUS vs. LISAF30	1	58	15	0.2586207
LISAF30 vs. MVF45	1	485	264	0.5443299
MVRESCUE vs. MVRESCUE_LATE	1	317	205	0.6466877

Characteristic	Value
Number of Interventions	17
Number of Studies	22
Total Number of Patients in Network	3531
Total Possible Pairwise Comparisons	136
Total Number of Pairwise Comparisons With Direct Data	16
Is the network connected?	TRUE
Number of Two-arm Studies	22
Number of Multi-Arms Studies	0
Total Number of Events in Network	1515
Number of Studies With No Zero Events	21
Number of Studies With At Least One Zero Event	1
Number of Studies with All Zero Events	0

eTable 4: Network characteristics for the outcome of requirement of IMV for the sub-group >30 weeks for subnetwork 1

comparison	n.studies	n.patients	n.outcomes	proportion
INSUREF22_30 vs. MVRESCUE	1	105	63	0.6000000
INSUREF40 vs. LISAF40	3	241	40	0.1659751
INSUREF40 vs. MVRESCUE	2	177	62	0.3502825
LMAF40 vs. MVF40_45	1	48	31	0.6458333
LMAF40 vs. MVRESCUE	1	103	53	0.5145631
MVF40_45 vs. NEBF22_30	1	64	33	0.5156250

**Characteristic**	**Value**
Number of Interventions	7
Number of Studies	9
Total Number of Patients in Network	738
Total Possible Pairwise Comparisons	21
Total Number of Pairwise Comparisons With Direct Data	6
Is the network connected?	TRUE
Number of Two-arm Studies	9
Number of Multi-Arms Studies	0
Total Number of Events in Network	282
Number of Studies With No Zero Events	9
Number of Studies With At Least One Zero Event	0
Number of Studies with All Zero Events	0

eTable 5: Network characteristics for the outcome of requirement of IMV for the sub-group >30 weeks for subnetwork 2

comparison	n.studies	n.patients	n.outcomes	proportion
INSUREF30 vs. LISAF30_35	1	350	103	0.2942857
INSUREF30 vs. LMAF30	1	70	0	0.0000000
INSUREF30_60 vs. LMAF30_60	3	210	67	0.3190476
INSURERESCUE vs. LISAF30_35	1	45	26	0.5777778
INSURERESCUE vs. LMAF30_60	1	26	4	0.1538462

Characteristic	Value
Number of Interventions	6
Number of Studies	7
Total Number of Patients in Network	701
Total Possible Pairwise Comparisons	15
Total Number of Pairwise Comparisons With Direct Data	5
Is the network connected?	TRUE
Number of Two-arm Studies	7
Number of Multi-Arms Studies	0
Total Number of Events in Network	200
Number of Studies With No Zero Events	6
Number of Studies With At Least One Zero Event	1
Number of Studies with All Zero Events	1

eTable 6: Matrix plot depicting the NMA effect estimate for the outcome of requirement of IMV for the sub-group >30 weeks for subnetwork 1

INSUREF22_30	1.02 (0.28, 4.12)	0.87 (0.2, 4.56)	0.82 (0.18, 3.75)	43207.48 (3.1, 601571399468434)	1.4 (0.48, 4.08)	21216.52 (1.39, 303831246653593)
0.98 (0.24, 3.59)	INSUREF40	0.85 (0.39, 1.97)	0.81 (0.2, 2.98)	41419.3 (3.08, 572654688722996)	1.37 (0.57, 3.02)	20418.8 (1.37, 292812968738608)
1.16 (0.22, 5.08)	1.18 (0.51, 2.57)	LISAF40	0.95 (0.18, 4.23)	48447.45 (3.4, 693034245573786)	1.61 (0.47, 4.8)	23798.64 (1.5, 346352079196944)
1.21 (0.27, 5.58)	1.24 (0.34, 5.11)	1.05 (0.24, 5.65)	LMAF40	51702.41 (4.39, 700729510456708)	1.7 (0.58, 5.07)	25624.44 (1.95, 353541874246666)
0 (0, 0.32)	0 (0, 0.32)	0 (0, 0.29)	0 (0, 0.23)	MVF40_45	0 (0, 0.42)	0.49 (0.16, 1.5)
0.71 (0.25, 2.06)	0.73 (0.33, 1.74)	0.62 (0.21, 2.13)	0.59 (0.2, 1.72)	30462.52 (2.38, 425558979683038)	MVRESCUE	15047.55 (1.05, 210603947515271)
0 (0, 0.72)	0 (0, 0.73)	0 (0, 0.67)	0 (0, 0.51)	2.03 (0.67, 6.38)	0 (0, 0.95)	NEBF22_30

eTable 7: Matrix plot depicting the NMA effect estimate for the outcome of requirement of IMV for the sub-group >30 weeks for subnetwork 2

INSUREF30	0.53 (0.01, 9.97)	1.23 (0.23, 6.68)	0.47 (0.15, 1.46)	0.01 (0, 1397090.61)	0.32 (0.01, 5.61)
1.9 (0.1, 86.89)	INSUREF30_60	2.32 (0.2, 75.92)	0.89 (0.06, 34.66)	0.02 (0, 3973715.29)	0.59 (0.3, 1.33)
0.81 (0.15, 4.27)	0.43 (0.01, 4.89)	INSURERESCUE	0.38 (0.11, 1.27)	0.01 (0, 1220243.56)	0.26 (0.01, 2.66)
2.13 (0.68, 6.65)	1.12 (0.03, 17.36)	2.63 (0.78, 9.22)	LISAF30_35	0.02 (0, 3077390.41)	0.68 (0.02, 9.58)
105.36 (0, 1897887769357.21)	51.23 (0, 1163760104937.7)	131.44 (0, 2448015264733.61)	49.48 (0, 907077727717.41)	LMAF30	30.93 (0, 698963524986.94)
3.16 (0.18, 131.7)	1.68 (0.75, 3.38)	3.82 (0.38, 116.35)	1.47 (0.1, 52.72)	0.03 (0, 6638175.92)	LMAF30_60

eTable 8: Certainty of evidence (CoE) for various comparisons for the outcome of IMV for the sub-group >30 weeks for sub-network 1 and 2

Comparison	CoE for direct evidence	CoE for indirect evidence	CoE for direct evidence
INSUREF40:LISAF40	Low	-	Low
INSUREF40:MVRESCUE	Very low	-	Very low
MVRESCUE:INSUREF22_30	Low	-	Low
MVRESCUE:LMAF40	Moderate	-	Moderate
MVF40-45:LMAF40	Moderate	-	Moderate
MVF40-45:NEBF22_30	Moderate	-	Moderate
INSUREF30_60: LMAF30_60	Low	-	Low
INSURERESCUE:LMAF30_60	Very low	-	Very low
INSURERESCUE:LISAF30_35	Very low	-	Very low
INSUREF30:LISAF30_35	Moderate	-	Moderate
INSUREF30:LMAF30	Very low	-	Very low
LMAF30:LMAF30_60	-	Very low	Very low
LISAF30_35:LMAF30	-	Very low	Very low
LISAF30_35:LMAF30_60	-	Very low	Very low
LISAF30_35:LMAF30_60	-	Very low	Very low
LISAF30_35:LMAF30	-	Very low	Very low
INSURERESCUE:LMAF30	-	Very low	Very low
INSUREF40:MVF40_45	-	Very low	Very low
INSUREF30_60:LMAF30	-	Very low	Very low
INSUREF30_60:LISAF30_35	-	Very low	Very low
INSUREF30_60:LMAF30	-	Very low	Very low
INSUREF30:LMAF30_60	-	Very low	Very low
INSUREF30:INSUREF30_60	-	Very low	Very low
INSUREF22_30:NEBF22_30	-	Low	Low
MVRESCUE:NEBFF22_30	-	Moderate	Moderate
MVF40_45:MVRESCUE	-	Moderate	Moderate
LMAF30:LMAF30_60	-	Very low	Very low
LISAF40:MVF40_45	-	Very low	Very low
INSUREF40:NEBF22_30	-	Very low	Very low
INSUREF40:MVF40_45	-	Very low	Very low
INSUREF30_60:LMAF30	-	Very low	Very low
INSUREF30_60:LISAF30_35	-	Very low	Very low
INSUREF30_60:INSURERESCUE	-	Very low	Very low
INSUREF30:LMAF30_60	-	Very low	Very low
INSUREF30:INSURERESCUE	-	Very low	Very low
INSUREF30:INSUREF30_60	-	Very low	Very low
INSUREF22_30:NEBF22_30	-	Low	Low
INSUREF22_30:MVF40_45	-	Low	Low
INSUREF22_30:LISAF40	-	Very low	Very low
INSUREF22_30:INSUREF40	-	Very low	Very low

eTable 9: Network characteristics for the outcome of mortality for the sub-group ≤30 weeks

comparison	n.studies	n.patients	n.outcomes	proportion
CPAP_only vs. INSURECLINICALRD	1	80	11	0.13750000
INRECSUREF30 vs. INSUREF30	1	218	60	0.27522936
INRECSUREF40 vs. INSUREF40	1	184	6	0.03260870
INSUREaAO2less0_22 vs. INSUREaAO2less0_36	1	60	10	0.16666667
INSUREaAO2less0_22 vs. MVRESCUE_LATE	1	68	7	0.10294118
INSUREaAO2less0_36 vs. INSURELAMCOUNT	1	380	20	0.05263158
INSURECLINICALRD vs. INSUREF40	1	159	21	0.13207547
INSURECLINICALRD vs. INSUREF50	1	153	20	0.13071895
INSURECLINICALRD vs. LISACLINICALRD	1	44	1	0.02272727
INSURECLINICALRD vs. MVRESCUE	1	278	26	0.09352518
INSUREF30 vs. LISAF30	4	419	31	0.07398568
INSUREF30 vs. MVF30	1	27	1	0.03703704
INSUREF40 vs. LISAF40	2	240	29	0.12083333
INSUREF45_50 vs. MVF45_50	1	42	9	0.21428571
INSURERESCUE vs. LISAF30	1	220	12	0.05454545
LISA_LUS vs. LISAF30	1	58	0	0.00000000
LISAF30 vs. MVF45_50	1	485	43	0.08865979
MVCLINICALRD vs. MVPROPHYLACTIC	1	207	15	0.07246377
MVF45_50 vs. MVPROPHYLACTIC	1	1316	208	0.15805471
MVPROPHYLACTIC vs. MVRESCUE_LATE	8	3949	1000	0.25322867
MVRESCUE vs. MVRESCUE_LATE	1	317	8	0.02523659

Characteristic	Value
Number of Interventions	22
Number of Studies	32
Total Number of Patients in Network	8904
Total Possible Pairwise Comparisons	231
Total Number of Pairwise Comparisons With Direct Data	21
Number of Two-arm Studies	32
Number of Multi-Arms Studies	0
Total Number of Events in Network	1538
Number of Studies With No Zero Events	27
Number of Studies With At Least One Zero Event	5
Number of Studies with All Zero Events	2

eTable 10: Matrix plot depicting the network estimates for the outcome of mortality for the sub-group ≤ 30 weeks

CPAP_only	0.23 (0.01, 4.5)	0.33 (0.02, 4.31)	0.1 (0, 1.92)	0.03 (0, 0.87)	0.55 (0.12, 2.16)	0.36 (0.02, 6.18)	0.73 (0.11, 4.29)	0.09 (0, 1.66)	0.63 (0.1, 3.74)	0.07 (0, 2.62)	0.17 (0.01, 3.35)	53.44 (0, 163789049485343)	0 (0, 0.91)	0.25 (0.01, 3.62)	0.89 (0.11, 6.83)	0.35 (0.02, 5.15)	704.9 (0.07, 57043977009.88)	0.19 (0.01, 2.37)	0.24 (0.02, 2.57)	0.56 (0.09, 3.17)	0.3 (0.02, 3.21)
4.35 (0.22, 91.83)	INRECSUREF30	1.41 (0.04, 45.1)	0.43 (0.03, 5.08)	0.14 (0.01, 2.44)	2.35 (0.17, 34.73)	1.56 (0.65, 3.78)	3.14 (0.18, 57.56)	0.39 (0.04, 3.2)	2.72 (0.16, 50.85)	0.33 (0.01, 7.52)	0.72 (0.11, 4.38)	230.07 (0, 658242073226653)	0 (0, 5.7)	1.07 (0.31, 3.54)	3.87 (0.19, 84.14)	1.51 (0.17, 12.47)	2919.91 (0.58, 201255562304.38)	0.83 (0.17, 3.78)	1.03 (0.18, 5.68)	2.41 (0.22, 28.95)	1.29 (0.22, 7.52)
3.07 (0.23, 49.79)	0.71 (0.02, 24.27)	INRECSUREF40	0.3 (0.01, 10.3)	0.1 (0, 4.45)	1.65 (0.19, 18.14)	1.11 (0.04, 34.19)	2.17 (0.35, 18.88)	0.27 (0.01, 9.03)	1.93 (0.17, 27.04)	0.23 (0, 12.97)	0.51 (0.01, 18.06)	171.07 (0, 543593357963256)	0 (0, 3.56)	0.76 (0.03, 20.61)	2.7 (0.33, 28.96)	1.07 (0.04, 29.62)	2218.83 (0.19, 197176732117.54)	0.59 (0.03, 14.12)	0.72 (0.04, 15.77)	1.7 (0.16, 23.29)	0.92 (0.05, 19.66)
10.27 (0.52, 255.06)	2.33 (0.2, 35.46)	3.34 (0.1, 123.67)	INSUREaAO2less0_22	0.33 (0.06, 1.37)	5.51 (0.41, 98.45)	3.64 (0.36, 48.18)	7.38 (0.43, 162.11)	0.9 (0.07, 12.94)	6.42 (0.37, 141.26)	0.77 (0.1, 5.21)	1.68 (0.13, 26.76)	557.27 (0, 1730129116618140)	0 (0, 13.8)	2.47 (0.29, 28.28)	9.12 (0.44, 233.06)	3.49 (0.4, 40.24)	7224.94 (0.93, 551053098299.5)	1.9 (0.28, 18.23)	2.33 (0.41, 19.67)	5.66 (0.52, 83.64)	2.94 (0.54, 24.49)
32.3 (1.15, 1156.29)	7.31 (0.41, 170.99)	10.44 (0.22, 539.45)	3.04 (0.73, 16.28)	INSUREaAO2less0_36	17.36 (0.86, 464.05)	11.41 (0.73, 237.81)	23.19 (0.94, 744.7)	2.81 (0.15, 62.52)	20.18 (0.82, 649.09)	2.36 (0.73, 8.34)	5.28 (0.27, 127.48)	1782.14 (0, 5730592793739900)	0 (0, 51.43)	7.75 (0.57, 141.58)	28.65 (0.98, 1056.76)	10.97 (0.78, 200.08)	22950.96 (2.51, 1805690088475.75)	6.01 (0.52, 95.06)	7.37 (0.74, 104.85)	17.82 (1.06, 401.34)	9.29 (0.97, 130.64)
1.82 (0.46, 8.1)	0.42 (0.03, 5.89)	0.61 (0.06, 5.27)	0.18 (0.01, 2.46)	0.06 (0, 1.16)	INSURECLINICALRD	0.67 (0.05, 7.91)	1.33 (0.44, 4.11)	0.16 (0.01, 2.16)	1.15 (0.38, 3.59)	0.14 (0, 3.54)	0.31 (0.02, 4.39)	98.49 (0, 306604796851005)	0 (0, 1.47)	0.45 (0.04, 4.53)	1.64 (0.37, 7.41)	0.64 (0.06, 6.48)	1277.46 (0.15, 100164783509.69)	0.35 (0.04, 2.89)	0.44 (0.05, 3.05)	1.03 (0.36, 2.95)	0.55 (0.07, 3.77)
2.78 (0.16, 52.02)	0.64 (0.26, 1.53)	0.9 (0.03, 25.87)	0.27 (0.02, 2.78)	0.09 (0, 1.37)	1.5 (0.13, 19.23)	INSUREF30	2 (0.13, 32.46)	0.25 (0.03, 1.7)	1.74 (0.11, 28.32)	0.21 (0.01, 4.26)	0.46 (0.09, 2.25)	145.88 (0, 420078822913019)	0 (0, 3.44)	0.69 (0.28, 1.57)	2.47 (0.14, 47.63)	0.96 (0.13, 6.59)	1850.91 (0.4, 124388342866.1)	0.53 (0.14, 1.86)	0.66 (0.14, 2.87)	1.54 (0.16, 15.8)	0.83 (0.17, 3.81)
1.38 (0.23, 8.73)	0.32 (0.02, 5.51)	0.46 (0.05, 2.88)	0.14 (0.01, 2.32)	0.04 (0, 1.07)	0.75 (0.24, 2.26)	0.5 (0.03, 7.49)	INSUREF40	0.12 (0.01, 2.01)	0.87 (0.18, 4.21)	0.1 (0, 3.24)	0.23 (0.01, 4.09)	74.18 (0, 229102314815022)	0 (0, 1.21)	0.34 (0.02, 4.4)	1.23 (0.45, 3.37)	0.48 (0.03, 6.24)	954.98 (0.11, 77176021047.57)	0.27 (0.02, 2.84)	0.33 (0.03, 3.06)	0.77 (0.17, 3.53)	0.41 (0.04, 3.82)
11.32 (0.6, 252.25)	2.58 (0.31, 24.96)	3.69 (0.11, 122.54)	1.11 (0.08, 14)	0.36 (0.02, 6.78)	6.13 (0.46, 96.14)	4.03 (0.59, 33.03)	8.2 (0.5, 160.41)	INSUREF45_50	7.1 (0.43, 139.94)	0.85 (0.03, 20.61)	1.86 (0.2, 19.12)	620.33 (0, 1777966963865386)	0 (0, 15.11)	2.74 (0.48, 18.72)	10.13 (0.51, 231.03)	3.87 (0.49, 35.8)	7859.02 (1.19, 579691440589.74)	2.11 (0.5, 11.32)	2.62 (0.5, 16.53)	6.3 (0.6, 80.83)	3.3 (0.62, 21.64)
1.58 (0.27, 10.09)	0.37 (0.02, 6.38)	0.52 (0.04, 6.05)	0.16 (0.01, 2.67)	0.05 (0, 1.22)	0.87 (0.28, 2.64)	0.57 (0.04, 8.7)	1.15 (0.24, 5.6)	0.14 (0.01, 2.34)	INSUREF50	0.12 (0, 3.73)	0.26 (0.01, 4.74)	86.08 (0, 264842559426330)	0 (0, 1.39)	0.39 (0.03, 5.05)	1.42 (0.22, 9.25)	0.55 (0.04, 7.21)	1105.19 (0.12, 88898948027.1)	0.31 (0.03, 3.29)	0.38 (0.04, 3.56)	0.89 (0.19, 4.12)	0.48 (0.05, 4.41)
13.58 (0.38, 590.32)	3.07 (0.13, 89.48)	4.37 (0.08, 268.75)	1.3 (0.19, 9.87)	0.42 (0.12, 1.36)	7.3 (0.28, 241.89)	4.81 (0.23, 124.92)	9.77 (0.31, 380.59)	1.18 (0.05, 32.37)	8.47 (0.27, 332.01)	INSURELAMCOUNT	2.22 (0.09, 65.7)	755.29 (0, 2419641866860016)	0 (0, 23.31)	3.27 (0.18, 74.65)	12.02 (0.33, 537.94)	4.62 (0.25, 107.09)	9757.62 (0.94, 798503609377.26)	2.53 (0.16, 50.29)	3.12 (0.23, 56.2)	7.52 (0.34, 210.57)	3.94 (0.29, 69.94)
6.05 (0.3, 139.98)	1.39 (0.23, 9.06)	1.96 (0.06, 68.13)	0.59 (0.04, 7.92)	0.19 (0.01, 3.76)	3.26 (0.23, 53.36)	2.17 (0.45, 11.38)	4.36 (0.24, 88.19)	0.54 (0.05, 5.05)	3.79 (0.21, 77.35)	0.45 (0.02, 11.41)	INSURERESCUE	321.87 (0, 961958391083220)	0 (0, 8.14)	1.48 (0.38, 6.11)	5.39 (0.25, 128.34)	2.09 (0.23, 19.7)	4093.38 (0.69, 297972493023.82)	1.15 (0.22, 6.27)	1.42 (0.23, 9.22)	3.36 (0.29, 44.89)	1.78 (0.28, 12.11)
0.02 (0, 65789735.96)	0 (0, 13164165.17)	0.01 (0, 22077983.11)	0 (0, 5667600.26)	0 (0, 1979634.53)	0.01 (0, 35137419.39)	0.01 (0, 20685081.17)	0.01 (0, 48420311.88)	0 (0, 5024294.54)	0.01 (0, 42325140.9)	0 (0, 4986990.59)	0 (0, 9671205.18)	LISA_LUS	0 (0, 14031.75)	0 (0, 14109578.8)	0.02 (0, 60933008.41)	0.01 (0, 19964137.24)	26.55 (0, 31212245129599.6)	0 (0, 11041661.35)	0 (0, 13549939.55)	0.01 (0, 34881222.55)	0.01 (0, 17199523.87)
137665.06 (1.1, 14146780283534682)	31771.94 (0.18, 3627820113870202)	43845.03 (0.28, 5044825232591995)	12841.69 (0.07, 1516068875751933)	4120.08 (0.02, 506696645165575)	74252.34 (0.68, 7431911135910357)	49527.98 (0.29, 5602658701540000)	98609.92 (0.83, 10515298318277506)	12010.03 (0.07, 1372044183841253)	85965.96 (0.72, 9048484619125544)	9984.7 (0.04, 1222993278117658)	22542.06 (0.12, 2508501637953804)	34478489.48 (0, 1.26695304062168e+23)	LISACLINICALRD	33321.13 (0.21, 3785933109530622)	121981.13 (0.95, 12850208762517476)	47681.45 (0.28, 5255006982034971)	447388172.91 (9.34, 1.84900479836928e+21)	25751.73 (0.17, 2788400748300232)	31721.85 (0.21, 3386757159005555)	76521.05 (0.65, 8012161588688291)	40252.45 (0.28, 4313217957508552)
4.06 (0.28, 67.97)	0.93 (0.28, 3.24)	1.32 (0.05, 34.3)	0.41 (0.04, 3.49)	0.13 (0.01, 1.77)	2.2 (0.22, 24.76)	1.46 (0.64, 3.53)	2.94 (0.23, 41.95)	0.37 (0.05, 2.07)	2.55 (0.2, 36.76)	0.31 (0.01, 5.53)	0.68 (0.16, 2.61)	214.26 (0, 612817239323606)	0 (0, 4.82)	LISAF30	3.62 (0.23, 62.37)	1.41 (0.24, 8.11)	2730.27 (0.55, 186189294673.56)	0.78 (0.3, 2)	0.96 (0.28, 3.3)	2.26 (0.29, 20.09)	1.2 (0.34, 4.42)
1.12 (0.15, 9.15)	0.26 (0.01, 5.34)	0.37 (0.03, 3.06)	0.11 (0, 2.25)	0.03 (0, 1.02)	0.61 (0.13, 2.72)	0.4 (0.02, 7.35)	0.81 (0.3, 2.23)	0.1 (0, 1.96)	0.71 (0.11, 4.59)	0.08 (0, 3.02)	0.19 (0.01, 4)	60.04 (0, 188454531400011)	0 (0, 1.06)	0.28 (0.02, 4.32)	LISAF40	0.39 (0.02, 6.14)	783.74 (0.08, 63921753766.96)	0.22 (0.01, 2.84)	0.26 (0.02, 3.09)	0.63 (0.1, 3.92)	0.33 (0.03, 3.87)
2.89 (0.19, 48.38)	0.66 (0.08, 5.75)	0.94 (0.03, 24.49)	0.29 (0.02, 2.51)	0.09 (0, 1.28)	1.56 (0.15, 17.65)	1.04 (0.15, 7.47)	2.08 (0.16, 30.31)	0.26 (0.03, 2.05)	1.81 (0.14, 26.2)	0.22 (0.01, 3.98)	0.48 (0.05, 4.41)	153.96 (0, 444567484518302)	0 (0, 3.52)	0.71 (0.12, 4.09)	2.57 (0.16, 44.47)	MVCLINICALRD	1972.66 (0.3, 144248699118.41)	0.55 (0.13, 2.41)	0.68 (0.19, 2.37)	1.61 (0.2, 14.31)	0.86 (0.23, 3.15)
0 (0, 13.66)	0 (0, 1.71)	0 (0, 5.37)	0 (0, 1.08)	0 (0, 0.4)	0 (0, 6.47)	0 (0, 2.5)	0 (0, 9.37)	0 (0, 0.84)	0 (0, 8.18)	0 (0, 1.06)	0 (0, 1.45)	0.04 (0, 1005532879619.49)	0 (0, 0.11)	0 (0, 1.83)	0 (0, 12.26)	0 (0, 3.31)	MVF30	0 (0, 1.53)	0 (0, 1.98)	0 (0, 6.08)	0 (0, 2.54)
5.22 (0.42, 74.68)	1.2 (0.26, 5.75)	1.7 (0.07, 38.4)	0.53 (0.05, 3.59)	0.17 (0.01, 1.91)	2.82 (0.35, 26.3)	1.88 (0.54, 6.92)	3.77 (0.35, 45.54)	0.47 (0.09, 2)	3.27 (0.3, 39.8)	0.39 (0.02, 6.1)	0.87 (0.16, 4.51)	275.72 (0, 805028185494678)	0 (0, 5.91)	1.29 (0.5, 3.33)	4.65 (0.35, 68.97)	1.81 (0.42, 7.99)	3549.01 (0.65, 246374003290.87)	MVF45_50	1.23 (0.55, 2.76)	2.89 (0.47, 20.98)	1.54 (0.66, 3.79)
4.24 (0.39, 53.76)	0.98 (0.18, 5.71)	1.38 (0.06, 28.08)	0.43 (0.05, 2.46)	0.14 (0.01, 1.36)	2.29 (0.33, 18.52)	1.52 (0.35, 7.06)	3.06 (0.33, 32.63)	0.38 (0.06, 1.98)	2.65 (0.28, 28.5)	0.32 (0.02, 4.42)	0.71 (0.11, 4.37)	226.47 (0, 648106035144950)	0 (0, 4.66)	1.04 (0.3, 3.6)	3.78 (0.32, 49.62)	1.47 (0.42, 5.21)	2879.13 (0.5, 206179819890.33)	0.81 (0.36, 1.82)	MVPROPHYLACTIC	2.35 (0.46, 14.38)	1.25 (0.91, 1.81)
1.78 (0.32, 10.91)	0.41 (0.03, 4.59)	0.59 (0.04, 6.45)	0.18 (0.01, 1.93)	0.06 (0, 0.94)	0.97 (0.34, 2.78)	0.65 (0.06, 6.08)	1.29 (0.28, 6.02)	0.16 (0.01, 1.67)	1.12 (0.24, 5.29)	0.13 (0, 2.92)	0.3 (0.02, 3.44)	94.39 (0, 289951737719061)	0 (0, 1.54)	0.44 (0.05, 3.42)	1.59 (0.25, 10.01)	0.62 (0.07, 4.95)	1231.05 (0.16, 94509400074.54)	0.35 (0.05, 2.12)	0.43 (0.07, 2.18)	MVRESCUE	0.54 (0.09, 2.69)
3.36 (0.31, 41.47)	0.77 (0.13, 4.6)	1.09 (0.05, 21.63)	0.34 (0.04, 1.86)	0.11 (0.01, 1.00)	1.81 (0.27, 14.2)	1.21 (0.26, 5.71)	2.42 (0.26, 25.06)	0.3 (0.05, 1.61)	2.1 (0.23, 21.77)	0.25 (0.01, 3.42)	0.56 (0.08, 3.52)	179.28 (0, 524911720840992)	0 (0, 3.63)	0.83 (0.23, 2.93)	2.99 (0.26, 38.21)	1.16 (0.32, 4.26)	2287.27 (0.39, 163455368168.19)	0.65 (0.26, 1.52)	0.8 (0.55, 1.09)	1.86 (0.37, 11.03)	MVRESCUE_LATE

eTable 11: CoE for various comparisons for the outcome of mortality for the sub-group ≤ 30 weeks

Comparison	CoE for direct evidence	CoE for indirect evidence	CoE for NMA estimate
INRECSUREF30:INSUREF30	Low	-	Low
INRECSUREF40:INSUREF40	Very low	-	Very low
INSUREaAO2less_0_22:INSUREaAO2less_0_36	Very low	-	Very low
INSUREaAO2less_0_22:MVRESCUE_LATE	Very low	-	Very low
INSUREaAO2less_0_36:INSURELAMCOUNT	Low	-	Low
INSURECLINICALRD:LISACLINICALRD	Very low	-	Very low
INSURECLINICALRD:INSUREF45_50	Low	-	Low
INSUREF30:LISAF30	Moderate	-	Moderate
INSUREF30:MVF30	Very low	-	Very low
INSUREF40:LISAF40	Moderate	-	Moderate
INSUREF45_50:MVF45_50	Very low	-	Very low
INSURERESCUE:LISAF30	Low	-	Low
LISAF30:MVF45_50	Moderate	-	Moderate
MVF45_50:MVPROPHYLACTIC	Moderate	-	Moderate
MVPROPHYLACTIC:MVRESCUE_LATE	Moderate	-	Moderate
MVRESCUE:MVRESCUE_LATE	Low	-	Low
INSURECLINICALRD:MVRESCUE	Moderate	Low	Moderate
INSURECLINICALRD:INSUREF40	Low	Low	Low
INSUREF40:MVRESCUE	-	Low	Low
CPAP_only:INRECSUREF30	-	Low	Low
CPAP_only:INRECSUREF40	-	Very low	Very low
CPAP_only:INSUREaAO2less_0_22	-	Very low	Very low
CPAP_only:INSUREaAO2less_0_36	-	Very low	Very low
CPAP_only:INSUREF30	-	Very low	Very low
CPAP_only:INSUREF40	-	Very low	Very low
CPAP_only:INSUREF45_50	-	Very low	Very low
CPAP_only:INSURELAMCOUNT	-	Very low	Very low
CPAP_only:INSURERESCUE	-	Very low	Very low
CPAP_only:LISACLINICALRD	-	Very low	Very low
INSURECLINICALRD: CPAP_only	Very low	-	Very low
CPAP_only:LISAF30	-	Very low	Very low
CPAP_only:LISAF40	-	Very low	Very low
CPAP_only:MVF30	-	Very low	Very low
CPAP_only:MVF45_50	-	Very low	Very low
CPAP_only:MVPROPHYLACTIC	-	Very low	Very low
CPAP_only:MVRESCUE	-	Very low	Very low
CPAP_only:MVRESCUE_LATE	-	Very low	Very low
INRECSUREF30:INRECSUREF40	-	Low	Low
INRECSUREF30:INSUREaAO2less_0_22	-	Very low	Very low
INRECSUREF30:INSUREaAO2less_0_36	-	Very low	Very low
INRECSUREF30:INSURECLINICALRD	-	Low	Low
INRECSUREF30:INSUREF40	-	Low	Low
INRECSUREF30:INSUREF45_50	-	Very low	Very low
INRECSUREF30:INSURELAMCOUNT	-	Very low	Very low
INRECSUREF30:INSURERESCUE	-	Low	Low
INRECSUREF30:LISACLINICALRD	-	Very low	Very low
INRECSUREF30:LISAF30	-	Low	Low
INRECSUREF30:LISAF40	-	Low	Low
INRECSUREF30:MVF30	-	Very low	Very low
INRECSUREF30:MVF45_50	-	Very low	Very low
INRECSUREF30:MVPROPHYLACTIC	-	Low	Low
INRECSUREF30:MVRESCUE	-	Low	Low
INRECSUREF30:MVRESCUE_LATE	-	Low	Low
INRECSUREF40:INSUREaAO2less_0_22	-	Very low	Very low
INRECSUREF40:INSUREaAO2less_0_36	-	Very low	Very low
INRECSUREF40:INSURECLINICALRD	-	Low	Low
INRECSUREF40:INSUREF30	-	Low	Low
INRECSUREF40:INSUREF45_50	-	Very low	Very low
INRECSUREF40:INSURELAMCOUNT	-	Very low	Very low
INRECSUREF40:INSURERESCUE	-	Very low	Very low
INRECSUREF40:LISACLINICALRD	-	Very low	Very low
INRECSUREF40:LISAF30	-	Low	Low
INRECSUREF40:LISAF40	-	Low	Low
INRECSUREF40:MVF30	-	Very low	Very low
INRECSUREF40:MVF45_50	-	Very low	Very low
INRECSUREF40:MVPROPHYLACTIC	-	Low	Low
INRECSUREF40:MVRESCUE	-	Low	Low
INRECSUREF40:MVRESCUE_LATE	-	Low	Low
INSUREaAO2less_0_22:INSURECLINICALRD	-	Very low	Very low
INSUREaAO2less_0_22:INSUREF30	-	Very low	Very low
INSUREaAO2less_0_22:INSUREF40	-	Very low	Very low
INSUREaAO2less_0_22:INSUREF45_50	-	Very low	Very low
INSUREaAO2less_0_22:INSURELAMCOUNT	-	Very low	Very low
INSUREaAO2less_0_22:INSURERESCUE	-	Very low	Very low
INSUREaAO2less_0_22:LISACLINICALRD	-	Very low	Very low
INSUREaAO2less_0_22:LISAF30	-	Very low	Very low
INSUREaAO2less_0_22:LISAF40	-	Very low	Very low
INSUREaAO2less_0_22:MVF30	-	Very low	Very low
INSUREaAO2less_0_22:MVF45_50	-	Very low	Very low
INSUREaAO2less_0_22:MVPROPHYLACTIC	-	Very low	Very low
INSUREaAO2less_0_22:MVRESCUE	-	Very low	Very low
INSUREaAO2less_0_36:INSURECLINICALRD	-	Very low	Very low
INSUREaAO2less_0_36:INSUREF30	-	Very low	Very low
INSUREaAO2less_0_36:INSUREF40	-	Very low	Very low
INSUREaAO2less_0_36:INSUREF45_50	-	Very low	Very low
INSUREaAO2less_0_36:INSURERESCUE	-	Very low	Very low
INSUREaAO2less_0_36:LISACLINICALRD	-	Very low	Very low
INSUREaAO2less_0_36:LISAF30	-	Very low	Very low
INSUREaAO2less_0_36:LISAF40	-	Very low	Very low
INSUREaAO2less_0_36:MVF30	-	Very low	Very low
INSUREaAO2less_0_36:MVF45_50	-	Very low	Very low
INSUREaAO2less_0_36:MVPROPHYLACTIC	-	Very low	Very low
INSUREaAO2less_0_36:MVRESCUE	-	Very low	Very low
INSUREaAO2less_0_36:MVRESCUE_LATE	-	Very low	Very low
INSURECLINICALRD:INSUREF30	-	Low	Low
INSURECLINICALRD:INSUREF45_50	-	Very low	Very low
INSURECLINICALRD:INSURELAMCOUNT	-	Very low	Very low
INSURECLINICALRD:INSURERESCUE	-	Low	Low
INSURECLINICALRD:LISAF30	-	Low	Low
INSURECLINICALRD:LISAF40	-	Low	Low
INSURECLINICALRD:MVF30	-	Very low	Very low
INSURECLINICALRD:MVF45_50	-	Low	Low
INSURECLINICALRD:MVPROPHYLACTIC	-	Low	Low
INSURECLINICALRD:MVRESCUE_LATE	-	Low	Low
INSUREF30:INSUREF40	-	Low	Low
INSUREF30:INSUREF45_50	-	Very low	Very low
INSUREF30:INSURELAMCOUNT	-	Very low	Very low
INSUREF30:INSURERESCUE	-	Low	Low
INSUREF30:LISACLINICALRD	-	Low	Low
INSUREF30:LISAF40	-	Low	Low
INSUREF30:MVF45_50	-	Moderate	Moderate
INSUREF30:MVPROPHYLACTIC	-	Moderate	Moderate
INSUREF30:MVRESCUE	-	Low	Low
INSUREF30:MVRESCUE_LATE	-	Moderate	Moderate
INSUREF40:INSUREF45_50	-	Very low	Very low
INSUREF40:INSURELAMCOUNT	-	Very low	Very low
INSUREF40:INSURERESCUE	-	Low	Low
INSUREF40:LISACLINICALRD	-	Very low	Very low
INSUREF40:LISAF30	-	Low	Low
INSUREF40:MVF30	-	Very low	Very low
INSUREF40:MVF45_50	-	Low	Low
INSUREF40:MVPROPHYLACTIC	-	Low	Low
INSUREF40:MVRESCUE_LATE	-	Low	Low
INSUREF40_45:INSURELAMCOUNT	-	Very low	Very low
INSUREF40_45:INSURERESCUE	-	Very low	Very low
INSUREF40_45:LISACLINICALRD	-	Very low	Very low
INSUREF40_45:LISAF30	-	Very low	Very low
INSUREF40_45:LISAF40	-	Very low	Very low
INSUREF40_45:MVF30	-	Very low	Very low
INSUREF40_45:MVPROPHYLACTIC	-	Very low	Very low
INSUREF40_45:MVRESCUE	-	Very low	Very low
INSUREF40_45:MVRESCUE_LATE	-	Very low	Very low
INSURELAMCOUNT:INSURERESCUE	-	Very low	Very low
INSURELAMCOUNT:LISACLINICALRD	-	Very low	Very low
INSURELAMCOUNT:LISAF30	-	Very low	Very low
INSURELAMCOUNT:LISAF40	-	Very low	Very low
INSURELAMCOUNT:MVF30	-	Very low	Very low
INSURELAMCOUNT:MVF40_50	-	Very low	Very low
INSURELAMCOUNT:MVPROPHYLACTIC	-	Very low	Very low
INSURELAMCOUNT:MVRESCUE	-	Very low	Very low
INSURELAMCOUNT:MVRESCUE_LATE	-	Very low	Very low
INSURERESCUE:LISACLINICALRD	-	Low	Low
INSURERESCUE:LISAF40	-	Low	Low
INSURERESCUE:MVF30	-	Very low	Very low
INSURERESCUE:MVF45_50	-	Low	Low
INSURERESCUE:MVPROPHYLACTIC	-	Low	Low
INSURERESCUE:MVRESCUE	-	Low	Low
INSURERESCUE:MVRESCUE_LATE	-	Low	Low
LISACLINICALRD:LISAF30	-	Low	Low
LISACLINICALRD:LISAF40	-	Low	Low
LISACLINICALRD:MVF30	-	Very low	Very low
LISACLINICALRD:MVF45_50	-	Low	Low
LISACLINICALRD:MVPROPHYLACTIC	-	Low	Low
LISACLINICALRD:MVRESCUE	-	Low	Low
LISACLINICALRD:MVRESCUE_LATE	-	Low	Low
LISAF30:LISAF40	-	Low	Low
LISAF30:LISA_LUS	Low	-	Low
LISAF30:MVF30	-	Low	Low
LISAF30:MVPROPHYLACTIC	-	Low	Low
LISAF30:MVRESCUE	-	Low	Low
LISAF30:MVRESCUE_LATE	-	Low	Low
LISAF40:MVF30	-	Low	Low
LISAF40:MVF45_50	-	Low	Low
LISAF40:MVPROPHYLACTIC	-	Low	Low
LISAF40:MVRESCUE_LATE	-	Low	Low
MVF30:MVF45_50	-	Very low	Very low
MVF30:MVPROPHYLACTIC	-	Very low	Very low
MVF30:MVRESCUE	-	Very low	Very low
MVF30:MVRESCUE_LATE	-	Very low	Very low
MVF45_50:MVRESCUE	-	Low	Low
MVF45_50:MVRESCUE_LATE	-	Low	Low
MVPROPHYLACTIC:MVRESCUE	-	Low	Low

eTable 12: Network characteristics for the outcome of mortality for the sub-group > 30 weeks

comparison	n.studies	n.patients	n.outcomes	proportion
INSURECLINICALRD vs. MVRESCUE	1	64	3	0.04687500
INSUREF22_30 vs. MVRESCUE	1	105	1	0.00952381
INSUREF40 vs. LISAF40	3	241	12	0.04979253
INSUREF40 vs. MVRESCUE	2	177	4	0.02259887

Characteristic	Value
Number of Interventions	5
Number of Studies	7
Total Number of Patients in Network	587
Total Possible Pairwise Comparisons	10
Total Number of Pairwise Comparisons With Direct Data	4
Is the network connected?	TRUE
Number of Two-arm Studies	7
Number of Multi-Arms Studies	0
Total Number of Events in Network	20
Number of Studies With No Zero Events	4
Number of Studies With At Least One Zero Event	3
Number of Studies with All Zero Events	2

eTable 13: Matrix plot depicting the network estimates for the outcome of mortality for the sub-group > 30 weeks

INSURECLINICALRD	35333.65 (0.13, 14516 510075697054)	0.43 (0.01, 19.51)	0.46 (0.01, 28.38)	0.41 (0.01, 6.85)
0 (0, 7.65)	INSUREF22_30	0 (0, 2.46)	0 (0, 3.01)	0 (0, 1.48)
2.31 (0.05, 165.67)	84464.22 (0.41, 31431 128300025560)	INSUREF40	1.06 (0.25, 5.04)	0.92 (0.07, 10.99)
2.18 (0.04, 197.38)	80006.19 (0.33, 30654 353822922416)	0.94 (0.2, 4.06)	LISAF40	0.86 (0.04, 15.1)
2.43 (0.15, 89.59)	88761.85 (0.68, 31626 555107483556)	1.09 (0.09, 13.64)	1.16 (0.07, 22.54)	MVRESCUE

eTable 14: CoE for various comparisons for the outcome of mortality for the sub-group > 30 weeks

Comparison	CoE for direct evidence	CoE for indirect evidence	CoE for NMA estimate
**INSUREF22_30:MVRESCUE**	Low	-	Low
**INSUREF40:LISAF40**	Very low	-	Very low
**INSURECLINICALRD:MVRESCUE**	Moderate	-	Moderate
**INSURECLINICALRD:INSUREF40**	Low	-	Low
**INSUREF40:MVRESCUE**	Very low	-	Very low
**INSURECLINICALRD:INSUREF22_3**	-	Low	Low
**INSURECLINICALRD:LISAF40**	-	Very low	Very low
**INSUREF22_30:INSUREF40**	-	Very low	Very low
**INSUREF22_30:LISAF40**	-	Very low	Very low
**LISAF40:MVRESCUE**	-	Very low	Very low

eTable 15: Network characteristics for the outcome of mortality or BPD in preterm neonates ≤ 36 weeks' gestation

comparison	n.studies	n.patients	n.outcomes	proportion
INSUREF22_30 vs. MVRESCUE	1	105	3	0.02857143
INSUREF30 vs. LISAF30	4	397	105	0.26448363
INSUREF30 vs. MVF30	1	27	4	0.14814815
INSUREF30_60 vs. LMAF30_60	1	60	5	0.08333333
INSURERESCUE vs. LISAF30	1	220	32	0.14545455
INSURERESCUE vs. LMAF30_60	1	26	15	0.57692308
LISAF30 vs. MVF45_50	1	485	226	0.46597938
LMAF30_40 vs. MVRESCUE	1	103	6	0.05825243
MVF45_50 vs. MVPROPHYLACTIC	1	1316	676	0.51367781
MVPROPHYLACTIC vs. MVRESCUE_LATE	5	3363	1133	0.33690158
MVRESCUE vs. MVRESCUE_LATE	1	317	24	0.07570978

Characteristic	Value
Number of Interventions	12
Number of Studies	18
Total Number of Patients in Network	6419
Total Possible Pairwise Comparisons	66
Total Number of Pairwise Comparisons With Direct Data	11
Is the network connected?	TRUE
Number of Two-arm Studies	18
Number of Multi-Arms Studies	0
Total Number of Events in Network	2229
Number of Studies With No Zero Events	17
Number of Studies With At Least One Zero Event	1
Number of Studies with All Zero Events	0

eTable 16: Matrix plot depicting the network estimates for the outcome of mortality or BPD in preterm neonates ≤ 36 weeks' gestation

INSUREF22_30	3.3 (0.17, 137.43)	1.86 (0.04, 139.86)	2.57 (0.12, 114.97)	2.33 (0.13, 94.24)	18.87 (0.58, 1974.37)	2.98 (0.12, 146.35)	2.64 (0.15, 102.16)	2.93 (0.18, 107.43)	2.33 (0.18, 75.47)	3.16 (0.2, 114.36)
0.3 (0.01, 5.8)	INSUREF30	0.56 (0.04, 5.83)	0.78 (0.28, 2.1)	0.71 (0.42, 1.14)	5.23 (0.4, 202.65)	0.9 (0.22, 3.53)	0.81 (0.34, 1.76)	0.9 (0.31, 2.42)	0.73 (0.16, 2.87)	0.99 (0.3, 2.64)
0.54 (0.01, 25.33)	1.79 (0.17, 23.46)	INSUREF30_60	1.39 (0.16, 14.59)	1.27 (0.13, 15.66)	10.05 (0.31, 714.77)	1.59 (0.23, 14.1)	1.45 (0.13, 19.14)	1.61 (0.14, 22.67)	1.3 (0.09, 21.73)	1.74 (0.14, 24.7)
0.39 (0.01, 8.27)	1.28 (0.48, 3.6)	0.72 (0.07, 6.15)	INSURERESCUE	0.91 (0.38, 2.19)	6.77 (0.48, 278.4)	1.15 (0.45, 3.01)	1.04 (0.35, 3.04)	1.15 (0.33, 3.98)	0.94 (0.18, 4.45)	1.26 (0.33, 4.32)
0.43 (0.01, 7.93)	1.4 (0.88, 2.4)	0.79 (0.06, 7.94)	1.1 (0.46, 2.65)	LISAF30	7.37 (0.61, 278.6)	1.26 (0.35, 4.62)	1.14 (0.6, 2.17)	1.26 (0.51, 3.12)	1.03 (0.25, 3.78)	1.39 (0.5, 3.41)
0.05 (0, 1.72)	0.19 (0, 2.49)	0.1 (0, 3.25)	0.15 (0, 2.1)	0.14 (0, 1.65)	LMAF30_40	0.17 (0, 2.87)	0.15 (0, 1.73)	0.17 (0, 1.78)	0.14 (0, 1.09)	0.19 (0.01, 1.86)
0.34 (0.01, 8.2)	1.12 (0.28, 4.46)	0.63 (0.07, 4.27)	0.87 (0.33, 2.2)	0.79 (0.22, 2.83)	5.95 (0.35, 269.6)	LMAF30_60	0.9 (0.22, 3.71)	1 (0.21, 4.66)	0.81 (0.12, 4.96)	1.09 (0.21, 5.05)
0.38 (0.01, 6.56)	1.23 (0.57, 2.9)	0.69 (0.05, 7.54)	0.96 (0.33, 2.83)	0.88 (0.46, 1.67)	6.45 (0.58, 234.12)	1.11 (0.27, 4.65)	MVF45_50	1.11 (0.59, 2.08)	0.9 (0.26, 2.84)	1.23 (0.57, 2.32)
0.34 (0.01, 5.55)	1.11 (0.41, 3.26)	0.62 (0.04, 7.3)	0.87 (0.25, 3.03)	0.79 (0.32, 1.95)	5.8 (0.56, 200.39)	1 (0.21, 4.76)	0.9 (0.48, 1.69)	MVPROPHYLACTIC	0.81 (0.28, 2.19)	1.1 (0.75, 1.44)
0.43 (0.01, 5.69)	1.37 (0.35, 6.35)	0.77 (0.05, 11.48)	1.07 (0.22, 5.62)	0.97 (0.26, 3.99)	7.07 (0.92, 220.56)	1.23 (0.2, 8.39)	1.11 (0.35, 3.88)	1.23 (0.46, 3.63)	MVRESCUE	1.34 (0.51, 3.65)
0.32 (0.01, 5.08)	1.01 (0.38, 3.31)	0.57 (0.04, 7)	0.79 (0.23, 3.03)	0.72 (0.29, 1.99)	5.35 (0.54, 185.15)	0.92 (0.2, 4.68)	0.82 (0.43, 1.77)	0.91 (0.69, 1.33)	0.75 (0.27, 1.97)	MVRESCUE_LATE

eTable 17: CoE for various comparisons for the outcome of mortality or BPD in preterm neonates ≤ 36 weeks' gestation

Comparison	CoE for direct evidence	CoE for indirect evidence	CoE for the network meta-
INSUREF22_30:MVRESCUE	Very low	-	Very low
INSUREF30:LISAF30	Moderate	-	Moderate
INSUREF30:MVF30	Very low	-	Very low
INSUREF30_60:LMAF30_60	Very low	-	Very low
INSURERESCUE:LISAF30	Moderate	-	Moderate
INSURERESCUE:LMAF30_60	Very low	-	Very low
LISAF30:MVF45_50	Moderate	-	Moderate
LMAF30_40:MVRESCUE	Very low	-	Very low
MVF45_50:MVPROPHYLACTIC	Moderate	-	Moderate
MVPROPHYLACTIC:MVRESCUE_LATE	Moderate	-	Moderate
MVRESCUE:MVRESCUE_LATE	Low	-	Low
INSUREF22_30:INSUREF30	-	Very low	Very low
INSUREF22_30:INSUREF30_60	-	Very low	Very low
INSUREF22_30:INSURERESCUE	-	Very low	Very low
INSUREF22_30:LISAF30	-	Very low	Very low
INSUREF22_30:LMAF30_40	-	Very low	Very low
INSUREF22_30:LMAF30_60	-	Very low	Very low
INSUREF22_30:MVF30	-	Very low	Very low
INSUREF22_30:MVF45_50	-	Very low	Very low
INSUREF22_30:MVPROPHYLACTIC	-	Very low	Very low
INSUREF22_30:MVRESCUE_LATE	-	Very low	Very low
INSUREF30:INSUREF30_60	-	Very low	Very low
INSUREF30:INSURERESCUE	-	Moderate	Moderate
INSUREF30:LMAF30_40	-	Very low	Very low
INSUREF30:LMAF30_60	-	Very low	Very low
INSUREF30:MVF45_50	-	Moderate	Moderate
INSUREF30:MVPROPHYLACTIC	-	Moderate	Moderate
INSUREF30:MVRESCUE	-	Low	Low
INSUREF30:MVRESCUE_LATE	-	Moderate	Moderate
INSUREF30_60:INSURERESCUE	-	Very low	Very low
INSUREF30_60:LISAF30	-	Very low	Very low
INSUREF30_60:LMAF30_40	-	Very low	Very low
INSUREF30_60:MVF30	-	Very low	Very low
INSUREF30_60:MVF45_50	-	Very low	Very low
INSUREF30_60:MVPROPHYLACTIC	-	Very low	Very low
INSUREF30_60:MVRESCUE	-	Very low	Very low
INSUREF30_60:MVRESCUE_LATE	-	Very low	Very low
INSURERESCUE:LMAF30_40	-	Very low	Very low
INSURERESCUE:MVF30	-	Very low	Very low
INSURERESCUE:MVF45_50	-	Moderate	Moderate
INSURERESCUE:MVPROPHYLACTIC	-	Moderate	Moderate
INSURERESCUE:MVRESCUE	-	Low	Low
INSURERESCUE:MVRESCUE_LATE	-	Moderate	Moderate
LISAF30:LMAF30_40	-	Very low	Very low
LISAF30:LMAF30_60	-	Very low	Very low
LISAF30:MVF30	-	Very low	Very low
LISAF30:MVPROPHYLACTIC	-	Moderate	Moderate
LISAF30:MVRESCUE	-	Low	Low
LISAF30:MVRESCUE_LATE	-	Moderate	Moderate
LMAF30_40:LMAF30_60	-	Very low	Very low
LMAF30_40:MVF30	-	Very low	Very low
LMAF30_40:MVF45_50	-	Very low	Very low
LMAF30_40:MVPROPHYLACTIC	-	Very low	Very low
LMAF30_40:MVRESCUE_LATE	-	Very low	Very low
LMAF30_60:MVF30	-	Very low	Very low
LMAF30_60:MVF45_50	-	Very low	Very low
LMAF30_60:MVPROPHYLACTIC	-	Very low	Very low
LMAF30_60:MVRESCUE	-	Very low	Very low
LMAF30_60:MVRESCUE_LATE	-	Very low	Very low
MVF30:MVF45_50	-	Very low	Very low
MVF30:MVPROPHYLACTIC	-	Very low	Very low
MVF30:MVRESCUE	-	Very low	Very low
MVF30:MVRESCUE_LATE	-	Very low	Very low
MVF45_50:MVRESCUE	-	Low	Low
MVF45_50:MVRESCUE_LATE	-	Low	Low
MVPROPHYLACTIC:MVRESCUE	-	Low	Low

eTable 18: Network characteristics for the outcome of IVH for the sub-group ≤ 30 weeks

treatment	n.studies	n.events	n.patients	min.outcome	max.outcome	av.outcome
INRECSUREF30	1	12	107	0.11214953	0.11214953	0.11214953
INSUREaAO2less0_22	2	3	62	0.00000000	0.08571429	0.04838710
INSUREaAO2less0_36	2	7	221	0.03030303	0.03191489	0.03167421
INSURECLINICALRD	2	3	220	0.01265823	0.01418440	0.01363636
INSUREF30	7	48	517	0.00000000	0.22115385	0.09284333
INSUREF40	4	35	383	0.05357143	0.16000000	0.09138381
INSURELAMCOUNT	1	6	192	0.03125000	0.03125000	0.03125000
INSURERESCUE	1	6	112	0.05357143	0.05357143	0.05357143
LISAF30	7	47	745	0.00000000	0.10526316	0.06308725
LISAF40	3	22	307	0.03571429	0.10000000	0.07166124
MVF_40_50	2	116	886	0.09836066	0.14330218	0.13092551
MVF30	1	1	14	0.07142857	0.07142857	0.07142857
MVPROPHYLACTIC	7	154	1221	0.03100775	0.25000000	0.12612613
MVRESCUE	2	13	291	0.02189781	0.06493506	0.04467354
MVRESCUE_LATE	8	104	776	0.03680982	0.25000000	0.13402062

Characteristic	Value
Number of Interventions	15
Number of Studies	25
Total Number of Patients in Network	6054
Total Possible Pairwise Comparisons	105
Total Number of Pairwise Comparisons With Direct Data	14
Is the network connected?	TRUE
Number of Two-arm Studies	25
Number of Multi-Arms Studies	0
Total Number of Events in Network	577
Number of Studies With No Zero Events	22
Number of Studies With At Least One Zero Event	3
Number of Studies with All Zero Events	0

eTable 19: Matrix plot depicting the network estimates for the outcome of IVH for the sub-group ≤ 30 weeks

INRECSUREF30	0.74 (0.02, 28.67)	44181.95 (0.17, 19610549132008756)	1.53 (0.02, 109.15)	1.38 (0.27, 7.04)	13.46 (0.1, 3051.49)	44992.76 (0.14, 19669406254881316)	0.84 (0.07, 12.6)	1.17 (0.19, 8.8)	10.03 (0.06, 2472.81)	1.55 (0.14, 20.71)	1.29 (0.02, 79.91)	1.24 (0.08, 25.1)	2.51 (0.09, 89.09)	1.38 (0.08, 30.08)
1.35 (0.03, 47.45)	INSUREaAO2less0_22	54522.47 (0.49, 20888917009552720)	2.07 (0.05, 79.02)	1.87 (0.07, 44.85)	18.08 (0.2, 2572.68)	55070.92 (0.38, 21249506937451864)	1.13 (0.03, 41.42)	1.59 (0.08, 35.65)	13.45 (0.13, 2117.54)	2.1 (0.16, 30.74)	1.73 (0.01, 243.5)	1.67 (0.21, 15.67)	3.41 (0.24, 53.94)	1.86 (0.25, 15.54)
0 (0, 5.78)	0 (0, 2.05)	INSUREaAO2less0_36	0 (0, 9.66)	0 (0, 7.07)	0 (0, 143.66)	0.98 (0.15, 6.23)	0 (0, 4.99)	0 (0, 5.92)	0 (0, 112.68)	0 (0, 6.8)	0 (0, 14.2)	0 (0, 4.85)	0 (0, 11.48)	0 (0, 5.24)
0.66 (0.01, 43.84)	0.48 (0.01, 19.06)	28906.21 (0.1, 1227418776632542 0)	INSURECLINICALRD	0.91 (0.02, 44.41)	8.14 (0.72, 275.72)	29000.67 (0.09, 1265236501775362 8)	0.55 (0.01, 37.88)	0.77 (0.02, 36.05)	6.09 (0.43, 237.1)	1.03 (0.03, 33.86)	0.84 (0, 186.72)	0.82 (0.04, 19.34)	1.64 (0.15, 21.04)	0.91 (0.05, 19.85)
0.72 (0.14, 3.67)	0.54 (0.02, 14.46)	31710.85 (0.14, 13685943880681600)	1.1 (0.02, 57.51)	INSUREF30	9.72 (0.09, 1735.79)	31704.7 (0.11, 1394398366254075 8)	0.61 (0.08, 5.2)	0.86 (0.34, 2.56)	7.24 (0.06, 1417.83)	1.12 (0.19, 8.16)	0.93 (0.02, 42.87)	0.89 (0.09, 11.11)	1.82 (0.09, 43.76)	1 (0.09, 13.52)
0.07 (0, 10.23)	0.06 (0, 4.97)	3220.91 (0.01, 1500725401510080)	0.12 (0, 1.38)	0.1 (0, 10.84)	INSUREF40	3203.38 (0.01, 1544554742979688)	0.06 (0, 8.93)	0.09 (0, 8.9)	0.74 (0.26, 2.09)	0.12 (0, 8.98)	0.09 (0, 38.38)	0.09 (0, 5.55)	0.19 (0, 6.89)	0.11 (0, 5.77)
0 (0, 7.12)	0 (0, 2.66)	1.02 (0.16, 6.57)	0 (0, 11.68)	0 (0, 8.74)	0 (0, 174.56)	INSURELAMCOUNT	0 (0, 6.2)	0 (0, 7.32)	0 (0, 135.9)	0 (0, 8.48)	0 (0, 17.13)	0 (0, 6)	0 (0, 14.22)	0 (0, 6.54)
1.19 (0.08, 15.15)	0.89 (0.02, 30.04)	52173.74 (0.2, 2298250141219414 0)	1.81 (0.03, 115.61)	1.64 (0.19, 12.04)	16.04 (0.11, 3298.18)	52037.32 (0.16, 23019514503222228)	INSURERESCUE	1.4 (0.23, 8.62)	11.9 (0.08, 2668.08)	1.87 (0.17, 20.83)	1.51 (0.02, 111.86)	1.49 (0.09, 25.44)	3.04 (0.1, 91.96)	1.66 (0.09, 30.34)
0.85 (0.11, 5.18)	0.63 (0.03, 12.92)	35936.8 (0.17, 1520101220573705 4)	1.29 (0.03, 54.08)	1.17 (0.39, 2.97)	11.29 (0.11, 1707.18)	36071.62 (0.14, 15773918902528508)	0.71 (0.12, 4.27)	LISAF30	8.41 (0.07, 1397.07)	1.33 (0.27, 6.47)	1.07 (0.02, 54.66)	1.06 (0.12, 9.45)	2.15 (0.12, 39.1)	1.18 (0.12, 11.61)
0.1 (0, 15.71)	0.07 (0, 7.54)	4392.02 (0.01, 2058974248104998)	0.16 (0, 2.33)	0.14 (0, 16.58)	1.35 (0.48, 3.87)	4350.55 (0.01, 2159064423061259)	0.08 (0, 13.3)	0.12 (0, 13.52)	LISAF40	0.16 (0, 13.68)	0.13 (0, 56.7)	0.13 (0, 8.55)	0.26 (0, 10.78)	0.14 (0, 8.92)
0.64 (0.05, 6.92)	0.48 (0.03, 6.24)	26619.11 (0.15, 10797971786696462)	0.98 (0.03, 28.62)	0.89 (0.12, 5.21)	8.46 (0.11, 1017.55)	26700.34 (0.12, 11049597104335474)	0.54 (0.05, 5.83)	0.75 (0.15, 3.65)	6.29 (0.07, 824.82)	MVF_40_50	0.82 (0.01, 54.37)	0.8 (0.18, 3.61)	1.62 (0.14, 18.24)	0.89 (0.17, 4.61)
0.78 (0.01, 48.62)	0.58 (0, 87.69)	37094.78 (0.07, 17009659617679290)	1.19 (0.01, 279.01)	1.07 (0.02, 49.17)	10.64 (0.03, 6177.56)	37132.98 (0.06, 17302157143894580)	0.66 (0.01, 51.98)	0.93 (0.02, 50.17)	7.91 (0.02, 4974.56)	1.23 (0.02, 90.02)	MVF30	0.98 (0.01, 92.73)	1.99 (0.02, 272.27)	1.09 (0.01, 108.71)
0.81 (0.04, 13.08)	0.6 (0.06, 4.88)	32930.08 (0.21, 13074644382847722)	1.22 (0.05, 25.39)	1.12 (0.09, 11.09)	10.55 (0.18, 1002.61)	33146.07 (0.17, 13431796986185328)	0.67 (0.04, 11.34)	0.95 (0.11, 8.36)	7.85 (0.12, 829.15)	1.25 (0.28, 5.66)	1.02 (0.01, 85.58)	MVPROPHYLACTIC	2.02 (0.3, 13.63)	1.11 (0.55, 2.21)
0.4 (0.01, 11.58)	0.29 (0.02, 4.23)	16631.25 (0.09, 6674949872321061)	0.61 (0.05, 6.48)	0.55 (0.02, 10.87)	5.15 (0.15, 330.66)	16666.37 (0.07, 6749610385614670)	0.33 (0.01, 10.13)	0.47 (0.03, 8.59)	3.84 (0.09, 275.93)	0.62 (0.05, 7.05)	0.5 (0, 61.68)	0.49 (0.07, 3.32)	MVRESCUE	0.55 (0.09, 3.22)
0.73 (0.03, 12.83)	0.54 (0.06, 3.93)	29491.35 (0.19, 11889850481059400)	1.1 (0.05, 21.37)	1 (0.07, 11.07)	9.49 (0.17, 864.81)	29660.79 (0.15, 11956325903934630)	0.6 (0.03, 11.13)	0.85 (0.09, 8.47)	7.04 (0.11, 712.11)	1.12 (0.22, 5.98)	0.92 (0.01, 81.37)	0.9 (0.45, 1.82)	1.82 (0.31, 10.83)	MVRESCUE_LATE

eTable 20: CoE for various comparisons for the outcome of IVH > grade 2 for the sub-group ≤ 30 weeks

Comparison	CoE for direct evidence	CoE for indirect evidence	CoE for NMA estimate
**INRECSUREF30:INSUREF30**	Low	-	Low
**INSUREaAO2less_0_22:INSUREaAO2less_0_36**	Very low	-	Very low
**INSUREaAO2less_0_22:MVRESCUE_LATE**	Very low	-	Very low
**INSUREaAO2less_0_36:INSURELAMCOUNT**	Very low	-	Very low
**INSUREF30:LISAF30**	Moderate	-	Moderate
**INSUREF30:MVF30**	Very low	-	Very low
**INSUREF40:LISAF40**	Moderate	-	Very low
**MVF40_50:MVPROPHYLACTIC**	Moderate	-	Very low
**MVPROPHYLACTIC:MVRESCUE_LATE**	Low	-	Low
**MVRESCUE:MVRESCUE_LATE**	Low	-	Low
**INRECSUREF30:INSUREaAO2less_0_22**	-	Very low	Very low
**INRECSUREF30:INSUREaAO2less_0_36**	-	Very low	Very low
**INRECSUREF30:INSURECLINICALRD**	-	Low	Low
**INRECSUREF30:INSUREF40**	-	Low	Low
**INRECSUREF30:INSURELAMCOUNT**	-	Very low	Very low
**INRECSUREF30:INSURERESCUE**	-	Low	Low
**INRECSUREF30:LISAF30**	-	Low	Low
**INRECSUREF30:LISAF40**	-	Low	Low
**INRECSUREF30:MVF30**	-	Very low	Very low
**INRECSUREF30:MVF40_50**	-	Low	Low
**INRECSUREF30:MVPROPHYLACTIC**	-	Low	Low
**INRECSUREF30:MVRESCUE**	-	Low	Low
**INRECSUREF30:MVRESCUE_LATE**	-	Low	Low
**INSUREaAO2less_0_22:INSURECLINICALRD**	-	Very low	Very low
**INSUREaAO2less_0_22:INSUREF30**	-	Very low	Very low
**INSUREaAO2less_0_22:INSUREF40**	-	Very low	Very low
**INSUREaAO2less_0_22:INSURELAMCOUNT**	-	Very low	Very low
**INSUREaAO2less_0_22:INSURERESCUE**	-	Very low	Very low
**INSUREaAO2less_0_22:INSURECLINICALRD**	-	Very low	Very low
**INSUREaAO2less_0_22:LISAF30**	-	Very low	Very low
**INSUREaAO2less_0_22:LISAF40**	-	Very low	Very low
**INSUREaAO2less_0_22:MVF30**	-	Very low	Very low
**INSUREaAO2less_0_22:MVF40_50**	-	Very low	Very low
**INSUREaAO2less_0_22:MVPROPHYLACTIC**	-	Very low	Very low
**INSUREaAO2less_0_22:MVRESCUE**	-	Very low	Very low
**INSUREaAO2less_0_22:INSUREF30**	-	Very low	Very low
**INSUREaAO2less_0_22:INSUREF40**	-	Very low	Very low
**INSUREaAO2less_0_22:INSURERESCUE**	-	Very low	Very low
**INSUREaAO2less_0_36:INSURECLINICALRD**	-	Very low	Very low
**INSUREaAO2less_0_36:INSUREF30**	-	Very low	Very low
**INSUREaAO2less_0_36:INSUREF40**	-	Very low	Very low
**INSUREaAO2less_0_36:INSUREF45_50**	-	Very low	Very low
**INSUREaAO2less_0_36:INSURERESCUE**	-	Very low	Very low
**INSUREaAO2less_0_36:LISAF30**	-	Very low	Very low
**INSUREaAO2less_0_36:LISAF40**	-	Very low	Very low
**INSUREaAO2less_0_36:MVF30**	-	Very low	Very low
**INSUREaAO2less_0_36:MVF45_50**	-	Very low	Very low
**INSUREaAO2less_0_36:MVPROPHYLACTIC**	-	Very low	Very low
**INSUREaAO2less_0_36:MVRESCUE**	-	Very low	Very low
**INSUREaAO2less_0_36:MVRESCUE_LATE**	-	Very low	Very low
**INSURECLINICALRD:INSUREF30**	-	Low	Low
**INSURECLINICALRD:INSURELAMCOUNT**	-	Very low	Very low
**INSURECLINICALRD:INSURERESCUE**	-	Low	Low
**INSURECLINICALRD:LISAF30**	-	Low	Low
**INSURECLINICALRD:LISAF40**	-	Low	Low
**INSURECLINICALRD:INSUREF40**	Low	-	Low
**INSURECLINICALRD:MVF40_50**	-	Low	Low
**INSURECLINICALRD:MVPROPHYLACTIC**	-	Low	Low
**INSURECLINICALRD:MVRESCUE_LATE**	-	Low	Low
**INSURECLINICALRD:MVF30**	-	Very low	Very low
**INSURECLINICALRD:MVFRESCUE**	Low	-	Low
**INSUREF30:INSUREF40**	-	Low	Low
**INSUREF30:INSUREF40_50**	-	Moderate	Moderate
**INSUREF30:INSURELAMCOUNT**	-	Very low	Very low
**INSUREF30:INSURERESCUE**	-	Low	Low
**INSUREF30:LISAF40**	-	Low	Low
**INSUREF30:MVF40_50**	-	Moderate	Moderate
**INSUREF30:MVPROPHYLACTIC**	-	Moderate	Moderate
**INSUREF30:MVRESCUE**	-	Low	Low
**INSUREF30:MVRESCUE_LATE**	-	Low	Low
**INSUREF40:INSUREF40_50**	-	Low	Low
**INSUREF40:INSURELAMCOUNT**	-	Very low	Very low
**INSUREF40:INSURERESCUE**	-	Low	Low
**INSUREF40:LISAF30**	-	Low	Low
**INSUREF40:MVF30**	-	Very low	Very low
**INSUREF40:MVF40_50**	-	Low	Low
**INSUREF40:MVPROPHYLACTIC**	-	Low	Low
**INSUREF40:MVRESCUE_LATE**	-	Low	Low
**INSUREF40:MVRESCUE**	-	Low	Low
**INSURELAMCOUNT:INSURERESCUE**	-	Very low	Very low
**INSURELAMCOUNT:LISAF30**	-	Very low	Very low
**INSURELAMCOUNT:LISAF40**	-	Very low	Very low
**INSURELAMCOUNT:MVF30**	-	Very low	Very low
**INSURELAMCOUNT:MVF40_50**	-	Very low	Very low
**INSURELAMCOUNT:MVPROPHYLACTIC**	-	Very low	Very low
**INSURELAMCOUNT:MVRESCUE**	-	Very low	Very low
**INSURELAMCOUNT:MVRESCUE_LATE**	-	Very low	Very low
**INSURERESCUE:LISAF40**	-	Low	Low
**INSURERESCUE:LISAF30**	Low	-	Low
**INSURERESCUE:MVF30**	-	Very low	Very low
**INSURERESCUE:MVF40_50**	-	Low	Low
**INSURERESCUE:MVPROPHYLACTIC**	-	Low	Low
**INSURERESCUE:MVRESCUE**	-	Low	Low
**INSURERESCUE:MVRESCUE_LATE**	-	Low	Low
**LISAF30:LISAF40**	-	Low	Low
**LISAF30:MVF40_50**	Moderate	-	Moderate
**LISAF30:MVF30**	-	Very low	Very low
**LISAF30:MVPROPHYLACTIC**	-	Moderate	Moderate
**LISAF30:MVRESCUE**	-	Low	Low
**LISAF30:MVRESCUE_LATE**	-	Low	Low
**LISAF40:MVF30**	-	Very low	Very low
**LISAF40:MVF40_50**	-	Low	Low
**LISAF40:MVPROPHYLACTIC**	-	Low	Low
**LISAF40:MVRESCUE_LATE**	-	Low	Low
**LISAF40:MVRESCUE**	-	Low	Low
**MVF30:MVF40_50**	-	Very low	Very low
**MVF30:MVPROPHYLACTIC**	-	Very low	Very low
**MVF30:MVRESCUE**	-	Very low	Very low
**MVF30:MVRESCUE_LATE**	-	Very low	Very low

eTable 21: Network characteristics for the outcome of IVH for the sub-group > 30 weeks

comparison	n.studies	n.patients	n.outcomes	proportion
INRECSUREF30 vs. INSUREF30	1	218	29	0.13302752
INSUREaAO2less0_22 vs. INSUREaAO2less0_36	1	60	1	0.01666667
INSUREaAO2less0_22 vs. MVRESCUE_LATE	1	68	8	0.11764706
INSUREaAO2less0_36 vs. INSURELAMCOUNT	1	380	12	0.03157895
INSURECLINICALRD vs. INSUREF40	1	159	7	0.04402516
INSURECLINICALRD vs. MVRESCUE	1	278	5	0.01798561
INSUREF30 vs. LISAF30	5	789	51	0.06463878
INSUREF30 vs. MVF30	1	27	2	0.07407407
INSUREF40 vs. LISAF40	3	610	51	0.08360656
INSURERESCUE vs. LISAF30	1	220	14	0.06363636
LISAF30 vs. MVF_40_50	1	485	42	0.08659794
MVF_40_50 vs. MVPROPHYLACTIC	1	1270	164	0.12913386
MVPROPHYLACTIC vs. MVRESCUE_LATE	6	1173	175	0.14919011
MVRESCUE vs. MVRESCUE_LATE	1	317	16	0.05047319

Characteristic	Value
Number of Interventions	4
Number of Studies	4
Total Number of Patients in Network	325
Total Possible Pairwise Comparisons	6
Total Number of Pairwise Comparisons With Direct Data	3
Is the network connected?	TRUE
Number of Two-arm Studies	4
Number of Multi-Arms Studies	0
Total Number of Events in Network	6
Number of Studies With No Zero Events	1
Number of Studies With At Least One Zero Event	3
Number of Studies with All Zero Events	2

eTable 22: Matrix plot depicting the Network estimates for the outcome of IVH for the sub-group > 30 weeks

INSUREF40	0.01 (0, 2685879.91)	154.93 (0, 31313622245302.5)	1 (0.14, 7.37)
191.74 (0, 32930645349582.1)	LISAF40	92351.34 (0, 1.70949298048217e+20)	192.03 (0, 35609025550141)
0.01 (0, 5062463.37)	0 (0, 94249759.41)	LMAF30	0.01 (0, 4517783.87)
1.01 (0.14, 6.97)	0.01 (0, 2951533.54)	154.54 (0, 28454998561966.4)	MVRESCUE

eTable 23: CoE for various comparisons for the outcome of mortality for the sub-group > 30 weeks

Comparison	CoE for direct evidence	CoE for indirect evidence	CoE for NMA estimate
**LMAF30:MVRESCUE**	Low	-	Low
**MVRESCUE:INSUREF40**	Low	-	Low
**INSUREF40:LISAF40**	Very low	-	Very low
**INSUREF40:LMAF40**	-	Very low	Very low
**LISAF40:LMAF30**	-	Very low	Very low
**LISAF40:MVRESCUE**	-	Very low	Very low

eTable 24: Network characteristics for the outcome of air leak in preterm neonates ≤ 36 weeks' gestation

comparison	n.studies	n.patients	n.outcomes	proportion
CPAP_only vs. INSURECLINICALRD	1	80	4	0.05000000
CPAP_only vs. NEBaAO2less0_22	1	32	8	0.25000000
INRECSUREF30 vs. INSUREF30	1	218	23	0.10550459
INRECSUREF40 vs. INSUREF40	1	184	5	0.02717391
INSUREaAO2less0_22 vs. INSUREaAO2less0_36	1	60	1	0.01666667
INSUREaAO2less0_22 vs. MVRESCUE_LATE	1	68	3	0.04411765
INSUREaAO2less0_36 vs. INSURELAMCOUNT	1	380	8	0.02105263
INSURECLINICALRD vs. INSUREF40	1	159	7	0.04402516
INSURECLINICALRD vs. LMACLINICALRD	1	50	0	0.00000000
INSURECLINICALRD vs. MVRESCUE	2	339	16	0.04719764
INSUREF22_30 vs. MVRESCUE	1	105	4	0.03809524
INSUREF30 vs. LISAF30	3	378	29	0.07671958
INSUREF30 vs. LMAF30	1	70	0	0.00000000
INSUREF30 vs. MVF30	1	27	1	0.03703704
INSUREF30_60 vs. LMAF30_60	2	120	12	0.10000000
INSUREF40 vs. LISAF40	3	409	32	0.07823961
INSUREF40 vs. MVRESCUE	2	177	13	0.07344633
INSUREF45 vs. MVF45	1	42	9	0.21428571
INSURERESCUE vs. LISAF30	1	220	12	0.05454545
INSURERESCUE vs. LISAF35	1	45	2	0.04444444
INSURERESCUE vs. LMAF30_60	1	26	4	0.15384615
LISAF30 vs. MVF45	1	485	36	0.07422680
LMAF30 vs. MVRESCUE	1	103	9	0.08737864
MVF45 vs. NEBF22_30	1	64	2	0.03125000
MVF50 vs. MVPROPHYLACTIC	1	1307	93	0.07115532
MVPROPHYLACTIC vs. MVRESCUE_LATE	8	3949	879	0.22258800
MVRESCUE vs. MVRESCUE_LATE	1	317	12	0.03785489

eTable 24: Network characteristics for the outcome of air leak in preterm neonates ≤ 36 weeks' gestation

Characteristic	Value
Number of Interventions	27
Number of Studies	41
Total Number of Patients in Network	9414
Total Possible Pairwise Comparisons	351
Total Number of Pairwise Comparisons With Direct Data	27
Is the network connected?	TRUE
Number of Two-arm Studies	41
Number of Multi-Arms Studies	0
Total Number of Events in Network	1224
Number of Studies With No Zero Events	32
Number of Studies With At Least One Zero Event	9
Number of Studies with All Zero Events	2

eTable 25: Matrix plot depicting the network estimates for the outcome of air leak in preterm neonates ≤ 36 weeks' gestation

CPAP_only	*	6.03 (0.15, 258.97)	2.94 (0.03, 223.13)	0 (0, 1.87)	1 (0.09, 11.28)	0 (0, 1.11)	*	*	9.8 (0.54, 215.02)	*	0 (0, 1.15)	*	*	*	6.63 (0.32, 167.99)	*	6.91 (0.27, 200.44)	*	*	*	5.59 (0.19, 186.61)	5.99 (0.24, 168.55)	8.36 (0.51, 160.53)	7.9 (0.33, 220.06)	1 (0.19, 5.25)	*
*	INRECSUREF30	*	*	*	*	*	1.85 (0.51, 6.94)	0.26 (0, 9.71)	*	1.27 (0.09, 17.73)	*	2.42 (0.27, 25.17)	1.19 (0.24, 6.33)	2.17 (0.03, 169.7)	*	*	*	0.6 (0.01, 17.03)	#	2.72 (0.37, 21.86)	*	*	*	*	*	0 (0, 0.39)
0.17 (0, 6.53)	*	INRECSUREF40	0.49 (0.01, 28.66)	0 (0, 0.27)	0.17 (0.01, 2.69)	0 (0, 0.17)	*	*	1.6 (0.19, 16.44)	*	0 (0, 0.17)	*	*	*	1.09 (0.11, 13.27)	*	1.14 (0.06, 24.16)	*	*	*	0.92 (0.04, 22.6)	0.99 (0.05, 20.05)	1.37 (0.12, 18.24)	1.3 (0.07, 25.97)	0.17 (0, 9.32)	*
0.34 (0, 36.64)	*	2.05 (0.03, 186.53)	INSUREaAO2less0_22	0 (0, 0.33)	0.34 (0.01, 18.93)	0 (0, 0.45)	*	*	3.29 (0.12, 178.36)	*	0 (0, 0.2)	*	*	*	2.24 (0.07, 134.62)	*	2.33 (0.07, 145.94)	*	*	*	1.83 (0.09, 76.53)	1.94 (0.12, 71.39)	2.77 (0.12, 127.79)	2.56 (0.17, 91.81)	0.34 (0, 48.53)	*
*	*	#	#	INSUREaAO2less0_36	*	*	*	*	#	*	0.56 (0.08, 3.19)	*	*	*	#	*	#	*	*	*	#	#	#	#	*	*
1 (0.09, 11.32)	*	6.05 (0.37, 109.99)	2.98 (0.05, 107.67)	0 (0, 1.53)	INSURECLINICALRD	0 (0, 0.85)	*	*	9.52 (2.04, 68.93)	*	0 (0, 0.93)	*	*	*	6.49 (1.06, 58.11)	*	6.86 (0.78, 73.83)	*	*	*	5.54 (0.52, 72.52)	5.93 (0.72, 61.09)	8.13 (2.1, 46.6)	#	1 (0.05, 18.67)	*
*	*	#	#	*	#	INSUREF22_30	*	*	#	*	*	*	*	*	#	*	#	*	*	*	#	#	#	#	*	*
*	0.54 (0.14, 1.96)	*	*	*	*	*	INSUREF30	0.14 (0, 4.05)	*	0.68 (0.07, 6.65)	*	1.3 (0.21, 8.99)	0.64 (0.24, 1.74)	1.17 (0.02, 74.82)	*	*	*	0.32 (0.01, 6.93)	#	1.47 (0.32, 7.3)	*	*	*	*	*	0 (0, 0.2)
*	3.84 (0.1, 286.39)	*	*	*	*	*	*	INSUREF30_60	*	4.88 (0.1, 440.02)	*	9.07 (0.59, 386.24)	4.51 (0.19, 254.77)	8.48 (0.08, 1529.58)	*	*	*	2.23 (0.57, 10.93)	#	10.48 (0.34, 699.65)	*	*	*	*	*	0 (0, 2.22)
0.1 (0, 1.84)	*	0.62 (0.06, 5.24)	0.3 (0.01, 8.67)	0 (0, 0.14)	0.11 (0.01, 0.49)	0 (0, 0.08)	*	*	INSUREF40	*	0 (0, 0.09)	*	*	*	0.68 (0.26, 1.74)	*	0.71 (0.09, 5.31)	*	*	*	0.57 (0.06, 5.32)	0.61 (0.08, 4.36)	0.86 (0.25, 2.77)	0.81 (0.11, 5.52)	0.1 (0, 2.82)	*
*	0.79 (0.06, 11.21)	*	*	*	*	*	1.46 (0.15, 15)	0.21 (0, 9.67)	*	INSUREF45	*	*	0.93 (0.12, 8)	1.72 (0.02, 163.21)	*	*	*	0.47 (0.01, 17.29)	#	2.13 (0.44, 12.84)	*	*	*	*	*	0 (0, 0.29)
*	*	*	#	1.79 (0.31, 12.06)	#	*	*	*	#	*	INSURELAMCOUNT	*	*	*	#	*	#	*	*	*	#	#	#	#	*	*
*	0.41 (0.04, 3.77)	*	*	*	*	*	0.77 (0.11, 4.66)	0.11 (0, 1.71)	*	0.52 (0.04, 6.65)	*	INSURERESCUE	0.5 (0.09, 2.28)	0.89 (0.02, 36.78)	*	*	*	0.26 (0.01, 2.63)	*	1.13 (0.15, 8.13)	*	*	*	*	*	0 (0, 0.15)
*	0.84 (0.16, 4.12)	*	*	*	*	*	1.56 (0.58, 4.13)	0.22 (0, 5.39)	*	1.08 (0.12, 8.03)	*	2.02 (0.44, 10.54)	LISAF30	1.82 (0.03, 103.64)	*	*	*	0.51 (0.01, 9.07)	#	2.3 (0.69, 7.87)	*	*	*	*	*	0 (0, 0.3)
*	0.46 (0.01, 34.87)	*	*	*	*	*	0.86 (0.01, 53.28)	0.12 (0, 12.66)	*	0.58 (0.01, 53.62)	*	1.13 (0.03, 46.91)	0.55 (0.01, 30.93)	LISAF35	*	*	*	0.27 (0, 23.37)	*	1.27 (0.02, 85.87)	*	*	*	*	*	0 (0, 0.26)
0.15 (0.01, 3.14)	*	0.92 (0.08, 9.5)	0.45 (0.01, 14.6)	0 (0, 0.22)	0.15 (0.02, 0.95)	0 (0, 0.13)	*	*	1.47 (0.57, 3.89)	*	0 (0, 0.13)	*	*	*	LISAF40	*	1.04 (0.11, 9.71)	*	*	*	0.84 (0.07, 9.52)	0.9 (0.09, 7.97)	1.26 (0.27, 5.72)	1.19 (0.13, 10.17)	0.15 (0, 4.78)	*
*	*	*	*	*	*	*	*	*	*	*	*	*	*	*	*	LMACLINICALRD	*	*	*	*	*	*	*	*	*	*
0.14 (0, 3.75)	*	0.88 (0.04, 17.48)	0.43 (0.01, 15.09)	0 (0, 0.22)	0.15 (0.01, 1.28)	0 (0, 0.13)	*	*	1.41 (0.19, 11.46)	*	0 (0, 0.13)	*	*	*	0.96 (0.1, 9.49)	*	LMAF30	*	*	*	0.81 (0.06, 10.22)	0.87 (0.09, 8.63)	1.2 (0.23, 6.61)	1.14 (0.12, 11.12)	0.15 (0, 5.53)	*
*	1.67 (0.06, 94.51)	*	*	*	*	*	3.08 (0.14, 142.37)	0.45 (0.09, 1.74)	*	2.14 (0.06, 144.64)	*	3.86 (0.38, 123.93)	1.96 (0.11, 82.28)	3.72 (0.04, 522.72)	*	*	*	LMAF30_60	#	4.56 (0.2, 227.79)	*	*	*	*	*	0 (0, 0.9)
*	0 (0, 0.5)	*	*	0 (0, 129.68)	*	*	0 (0, 0.85)	0 (0, 0.21)	*	0 (0, 0.74)	0 (0, 71.38)	0 (0, 1.37)	0 (0, 0.58)	0 (0, 2.16)	*	*	*	0 (0, 0.44)	MVF30	0 (0, 1.43)	*	*	*	*	*	0 (0, 0)
*	0.37 (0.05, 2.68)	*	*	*	*	*	0.68 (0.14, 3.12)	0.1 (0, 2.94)	*	0.47 (0.08, 2.29)	*	0.88 (0.12, 6.75)	0.44 (0.13, 1.46)	0.79 (0.01, 52.81)	*	*	*	0.22 (0, 5.06)	*	MVF45	*	*	*	*	*	0 (0, 0.12)
0.18 (0.01, 5.27)	*	1.08 (0.04, 25.28)	0.55 (0.01, 10.76)	0 (0, 0.24)	0.18 (0.01, 1.92)	0 (0, 0.16)	*	*	1.75 (0.19, 17.82)	*	0 (0, 0.15)	*	*	*	1.18 (0.11, 14.55)	*	1.23 (0.1, 15.98)	*	*	*	MVF50	1.07 (0.36, 3.2)	1.49 (0.22, 10.58)	1.4 (0.45, 4.86)	0.18 (0, 7.73)	*
0.17 (0.01, 4.16)	*	1.01 (0.05, 19.63)	0.51 (0.01, 8.14)	0 (0, 0.21)	0.17 (0.02, 1.39)	0 (0, 0.14)	*	*	1.63 (0.23, 12.64)	*	0 (0, 0.13)	*	*	*	1.1 (0.13, 10.6)	*	1.15 (0.12, 11.66)	*	*	*	0.93 (0.31, 2.79)	MVPROPHYLACTIC	1.39 (0.29, 7.08)	1.31 (0.85, 2.21)	0.17 (0, 6.16)	*
0.12 (0.01, 1.96)	*	0.73 (0.05, 8.54)	0.36 (0.01, 8.27)	0 (0, 0.16)	0.12 (0.02, 0.48)	0 (0, 0.09)	*	*	1.17 (0.36, 4.01)	*	0 (0, 0.1)	*	*	*	0.79 (0.17, 3.75)	*	0.83 (0.15, 4.27)	*	*	*	0.67 (0.09, 4.48)	0.72 (0.14, 3.48)	MVRESCUE	0.94 (0.21, 4.36)	0.12 (0, 3.06)	*
0.13 (0, 3.02)	*	0.77 (0.04, 14.08)	0.39 (0.01, 5.91)	0 (0, 0.16)	0.13 (0.01, 0.99)	0 (0, 0.11)	*	*	1.24 (0.18, 8.83)	*	0 (0, 0.1)	*	*	*	0.84 (0.1, 7.43)	*	0.88 (0.09, 8.24)	*	*	*	0.71 (0.21, 2.25)	0.77 (0.45, 1.18)	1.06 (0.23, 4.86)	MVRESCUE_LATE	0.13 (0, 4.48)	*
1 (0.19, 5.21)	*	6.03 (0.11, 366.18)	2.93 (0.02, 300.86)	0 (0, 2.03)	1 (0.05, 18.56)	0 (0, 1.25)	*	*	9.83 (0.35, 323.93)	*	0 (0, 1.24)	*	*	*	6.65 (0.21, 246.79)	*	6.9 (0.18, 294.01)	*	*	*	5.62 (0.13, 266.99)	6.01 (0.16, 246.45)	8.39 (0.33, 243.01)	7.91 (0.22, 320.09)	NEBaAO2less0_22	*
*	*	*	*	*	*	*	*	*	*	#	*	#	#	#	*	*	*	#	#	#	*	*	*	*	*	NEBF22_30

eTable 26: CoE for various comparisons for the outcome of air leak in preterm neonates ≤ 36 weeks' gestation

Comparison	CoE for direct	CoE for indirect	CoE for NMA
CPAP_only:INSURECLINICALRD	Very low	-	Very low
CPAP_only:NEBaAO2less0_22	Low	-	Low
INRECSUREF30:INSUREF30	Low	-	Low
INRECSUREF40:INSUREF40	Very low	-	Very low
INSUREaAO2less_0_22:INSUREaAO2less_0_36	Very low	-	Very low
INSUREaAO2less_0_22:MVRESCUE_LATE	Very low	-	Very low
INSUREaAO2less_0_36:INSURELAMCOUNT	Low	-	Low
INSURECLINICALRD:LMACLINICALRD	Low	-	Low
INSUREF22_30:MVRESCUE	Very low	-	Very low
INSUREF30:LISAF30	Low	-	Low
INSUREF30:LMAF30	Very low	-	Very low
INSUREF30:MVF30	Very low	-	Very low
INSUREF30_60:LMAF30_60	Very low	-	Very low
INSUREF40:LISAF40	Moderate	-	Moderate
INSUREF45:MVF45	Very low	-	Very low
INSURERESCUE:LISAF30	Low	-	Low
INSURERESCUE:LISAF35	Very low	-	Very low
INSURERESCUE:LMAF30_60	Very low	-	Very low
LISAF30:MVF45	Moderate	-	Moderate
LMAF30:MVRESCUE	Low	-	Low
MVF45:NEBF22_30	Low	-	Low
MVF50:MVPROPHYLACTIC	Moderate	-	Moderate
MVPROPHYLACTIC:MVRESCUE_LATE	Moderate	-	Moderate
MVRESCUE:MVRESCUE_LATE	Low	-	Low
INSUREF40:MVRESCUE	Very low	Very low	Very low
INSURECLINICALRD:MVRESCUE	Moderate	Very low	Low*
INSURECLINICALRD:INSUREF40	Very low	-	Very low
CPAP_only:INRECSUREF30	-	Very low	Very low
CPAP_only:INRECSUREF40	-	Very low	Very low
CPAP_only:INSUREaAO2less_0_22	-	Very low	Very low
CPAP_only:INSUREaAO2less_0_36	-	Very low	Very low
CPAP_only:INSUREF22_30	-	Very low	Very low
CPAP_only:INSUREF30	-	Very low	Very low
CPAP_only:INSUREF30_60	-	Very low	Very low
CPAP_only:INSUREF40	-	Very low	Very low
CPAP_only:INSUREF45	-	Very low	Very low
CPAP_only:INSURELAMCOUNT	-	Very low	Very low
CPAP_only:INSURERESCUE	-	Very low	Very low
CPAP_only:LISAF30	-	Very low	Very low
CPAP_only:LISAF35	-	Very low	Very low
CPAP_only:LISAF40	-	Very low	Very low
CPAP_only:LMACLINICALRD	-	Very low	Very low
CPAP_only:LMAF30	-	Very low	Very low
CPAP_only:LMAF30_60	-	Very low	Very low
CPAP_only:MVF30	-	Very low	Very low
CPAP_only:MVF45	-	Very low	Very low
CPAP_only:MVF50	-	Very low	Very low
CPAP_only:MVPROPHYLACTIC	-	Very low	Very low
CPAP_only:MVRESCUE	-	Very low	Very low
CPAP_only:MVRESCUE_LATE	-	Very low	Very low
CPAP_only:NEBF22_30	-	Very low	Very low
INRECSUREF30:INRECSUREF40	-	Very low	Very low
INRECSUREF30:INSUREaAO2less_0_22	-	Very low	Very low
INRECSUREF30:INSUREaAO2less_0_36	-	Very low	Very low
INRECSUREF30:INSURECLINICALRD	-	Very low	Very low
INRECSUREF30:INSUREF22_30	-	Low	Low
INRECSUREF30:INSUREF30_60	-	Very low	Very low
INRECSUREF30:INSUREF40	-	Very low	Very low
INRECSUREF30:INSUREF45	-	Very low	Very low
INRECSUREF30:INSURELAMCOUNT	-	Very low	Very low
INRECSUREF30:INSURERESCUE	-	Low	Low
INRECSUREF30:LISAF30	-	Very low	Very low
INRECSUREF30:LISAF35	-	Low	Low
INRECSUREF30:LISAF40	-	Very low	Very low
INRECSUREF30:LMACLINICALRD	-	Low	Low
INRECSUREF30:LMAF30	-	Very low	Very low
INRECSUREF30:LMAF30_60	-	Very low	Very low
INRECSUREF30:MVF30	-	Very low	Very low
INRECSUREF30:MVF45	-	Low	Low
INRECSUREF30:MVF50	-	Very low	Very low
INRECSUREF30:MVPROPHYLACTIC	-	Very low	Very low
INRECSUREF30:MVRESCUE	-	Very low	Very low
INRECSUREF30:MVRESCUE_LATE	-	Very low	Very low
INRECSUREF30:NEBaAO2less0_22	-	Very low	Very low
INRECSUREF30:NEBF22_30	-	Low	Low
INRECSUREF40:INSUREaAO2less_0_22	-	Very low	Very low
INRECSUREF40:INSUREaAO2less_0_36	-	Very low	Very low
INRECSUREF40:INSURECLINICALRD	-	Very low	Very low
INRECSUREF40:INSUREF22_30	-	Very low	Very low
INRECSUREF40:INSUREF30	-	Very low	Very low
INRECSUREF40:INSUREF30_60	-	Very low	Very low
INRECSUREF40:INSUREF45	-	Very low	Very low
INRECSUREF40:INSURELAMCOUNT	-	Very low	Very low
INRECSUREF40:INSURERESCUE	-	Very low	Very low
INRECSUREF40:LISAF30	-	Very low	Very low
INRECSUREF40:LISAF35	-	Very low	Very low
INRECSUREF40:LISAF40	-	Low	Low
INRECSUREF40:LMACLINICALRD	-	Very low	Very low
INRECSUREF40:LMAF30	-	Low	Low
INRECSUREF40:LMAF30_60	-	Very low	Very low
INRECSUREF40:MVF30	-	Very low	Very low
INRECSUREF40:MVF45	-	Very low	Very low
INRECSUREF40:MVF50	-	Very low	Very low
INRECSUREF40:MVPROPHYLACTIC	-	Very low	Very low
INRECSUREF40:MVRESCUE	-	Very low	Very low
INRECSUREF40:MVRESCUE_LATE	-	Very low	Very low
INRECSUREF40:NEBaAO2less0_22	-	Very low	Very low
INRECSUREF40:NEBF22_30	-	Very low	Very low
INSUREaAO2less_0_22:INSURECLINICALRD	-	Very low	Very low
INSUREaAO2less_0_22:INSUREF22_30	-	Very low	Very low
INSUREaAO2less_0_22:INSUREF30	-	Very low	Very low
INSUREaAO2less_0_22:INSUREF30_60	-	Very low	Very low
INSUREaAO2less_0_22:INSUREF40	-	Very low	Very low
INSUREaAO2less_0_22:INSUREF45	-	Very low	Very low
INSUREaAO2less_0_22:INSURELAMCOUNT	-	Very low	Very low
INSUREaAO2less_0_22:INSURERESCUE	-	Very low	Very low
INSUREaAO2less_0_22:LISAF30	-	Very low	Very low
INSUREaAO2less_0_22:LISAF35	-	Very low	Very low
INSUREaAO2less_0_22:LISAF40	-	Very low	Very low
INSUREaAO2less_0_22:LMACLINICALRD	-	Very low	Very low
INSUREaAO2less_0_22:LMAF30	-	Very low	Very low
INSUREaAO2less_0_22:LMAF30_60	-	Very low	Very low
INSUREaAO2less_0_22:MVF30	-	Very low	Very low
INSUREaAO2less_0_22:MVF45	-	Very low	Very low
INSUREaAO2less_0_22:MVF50	-	Very low	Very low
INSUREaAO2less_0_22:MVPROPHYLACTIC	-	Very low	Very low
INSUREaAO2less_0_22:MVRESCUE	-	Very low	Very low
INSUREaAO2less_0_22:NEBaAO2less0_22	-	Very low	Very low
INSUREaAO2less_0_22:NEBF22_30	-	Very low	Very low
INSUREaAO2less_0_36:INSURECLINICALRD	-	Very low	Very low
INSUREaAO2less_0_36:INSUREF22_30	-	Very low	Very low
INSUREaAO2less_0_36:INSUREF30	-	Very low	Very low
INSUREaAO2less_0_36:INSUREF30_60	-	Very low	Very low
INSUREaAO2less_0_36:INSUREF40	-	Very low	Very low
INSUREaAO2less_0_36:INSUREF45	-	Very low	Very low
INSUREaAO2less_0_36:INSURERESCUE	-	Very low	Very low
INSUREaAO2less_0_36:LISAF30	-	Very low	Very low
INSUREaAO2less_0_36:LISAF35	-	Very low	Very low
INSUREaAO2less_0_36:LISAF40	-	Very low	Very low
INSUREaAO2less_0_36:LMACLINICALRD	-	Very low	Very low
INSUREaAO2less_0_36:LMAF30	-	Very low	Very low
INSUREaAO2less_0_36:LMAF30_60	-	Very low	Very low
INSUREaAO2less_0_36:MVF30	-	Very low	Very low
INSUREaAO2less_0_36:MVF45	-	Very low	Very low
INSUREaAO2less_0_36:MVF50	-	Very low	Very low
INSUREaAO2less_0_36:MVPROPHYLACTIC	-	Very low	Very low
INSUREaAO2less_0_36:MVRESCUE	-	Very low	Very low
INSUREaAO2less_0_36:MVRESCUE_LATE	-	Very low	Very low
INSUREaAO2less_0_36:NEBaAO2less0_22	-	Very low	Very low
INSUREaAO2less_0_36:NEBF22_30	-	Very low	Very low
INSURECLINICALRD:INSUREF22_30	-	Very low	Very low
INSURECLINICALRD:INSUREF30	-	Very low	Very low
INSURECLINICALRD:INSUREF30_60	-	Very low	Very low
INSURECLINICALRD:INSUREF45	-	Very low	Very low
INSURECLINICALRD:INSURELAMCOUNT	-	Very low	Very low
INSURECLINICALRD:INSURERESCUE	-	Very low	Very low
INSURECLINICALRD:LISAF30	-	Very low	Very low
INSURECLINICALRD:LISAF35	-	Very low	Very low
INSURECLINICALRD:LISAF40	-	Very low	Very low
INSURECLINICALRD:LMAF30	-	Very low	Very low
INSURECLINICALRD:LMAF30_60	-	Very low	Very low
INSURECLINICALRD:MVF30	-	Very low	Very low
INSURECLINICALRD:MVF45	-	Very low	Very low
INSURECLINICALRD:MVF50	-	Low	Low
INSURECLINICALRD:MVPROPHYLACTIC	-	Low	Low
INSURECLINICALRD:MVRESCUE_LATE	-	Low	Low
INSURECLINICALRD:NEBaAO2less0_22	-	Very low	Very low
INSURECLINICALRD:NEBF22_30	-	Very low	Very low
INSUREF22_30:INSUREF30	-	Very low	Very low
INSUREF22_30:INSUREF30_60	-	Very low	Very low
INSUREF22_30:INSUREF40	-	Very low	Very low
INSUREF22_30:INSUREF45	-	Very low	Very low
INSUREF22_30:INSURELAMCOUNT	-	Very low	Very low
INSUREF22_30:INSURERESCUE	-	Very low	Very low
INSUREF22_30:LISAF30	-	Very low	Very low
INSUREF22_30:LISAF35	-	Very low	Very low
INSUREF22_30:LISAF40	-	Very low	Very low
INSUREF22_30:LMACLINICALRD	-	Very low	Very low
INSUREF22_30:LMAF30	-	Very low	Very low
INSUREF22_30:LMAF30_60	-	Very low	Very low
INSUREF22_30:MVF30	-	Very low	Very low
INSUREF22_30:MVF45	-	Very low	Very low
INSUREF22_30:MVF50	-	Very low	Very low
INSUREF22_30:MVPROPHYLACTIC	-	Very low	Very low
INSUREF22_30:MVRESCUE_LATE	-	Very low	Very low
INSUREF22_30:NEBaAO2less0_22	-	Very low	Very low
INSUREF22_30:NEBF22_30	-	Very low	Very low
INSUREF30:INSUREF30_60	-	Very low	Very low
INSUREF30:INSUREF40	-	Very low	Very low
INSUREF30:INSUREF45	-	Very low	Very low
INSUREF30:INSURELAMCOUNT	-	Very low	Very low
INSUREF30:INSURERESCUE	-	Very low	Very low
INSUREF30:LISAF35	-	Very low	Very low
INSUREF30:LISAF40	-	Very low	Very low
INSUREF30:LMACLINICALRD	-	Very low	Very low
INSUREF30:LMAF30_60	-	Very low	Very low
INSUREF30:MVF45	-	Low	Low
INSUREF30:MVF50	-	Very low	Very low
INSUREF30:MVPROPHYLACTIC	-	Very low	Very low
INSUREF30:MVRESCUE	-	Very low	Very low
INSUREF30:MVRESCUE_LATE	-	Very low	Very low
INSUREF30:NEBaAO2less0_22	-	Very low	Very low
INSUREF30:NEBF22_30	-	Low	Low
INSUREF30_60:INSUREF40	-	Very low	Very low
INSUREF30_60:INSUREF45	-	Very low	Very low
INSUREF30_60:INSURELAMCOUNT	-	Very low	Very low
INSUREF30_60:INSURERESCUE	-	Very low	Very low
INSUREF30_60:LISAF30	-	Very low	Very low
INSUREF30_60:LISAF35	-	Very low	Very low
INSUREF30_60:LISAF40	-	Very low	Very low
INSUREF30_60:LMACLINICALRD	-	Very low	Very low
INSUREF30_60:LMAF30	-	Very low	Very low
INSUREF30_60:MVF30	-	Very low	Very low
INSUREF30_60:MVF45	-	Very low	Very low
INSUREF30_60:MVF50	-	Very low	Very low
INSUREF30_60:MVPROPHYLACTIC	-	Very low	Very low
INSUREF30_60:MVRESCUE	-	Very low	Very low
INSUREF30_60:MVRESCUE_LATE	-	Very low	Very low
INSUREF30_60:NEBaAO2less0_22	-	Very low	Very low
INSUREF30_60:NEBF22_30	-	Very low	Very low
INSUREF40:INSUREF45	-	Very low	Very low
INSUREF40:INSURELAMCOUNT	-	Very low	Very low
INSUREF40:INSURERESCUE	-	Very low	Very low
INSUREF40:LISAF30	-	Very low	Very low
INSUREF40:LISAF35	-	Very low	Very low
INSUREF40:LMACLINICALRD	-	Very low	Very low
INSUREF40:LMAF30	-	Very low	Very low
INSUREF40:LMAF30_60	-	Very low	Very low
INSUREF40:MVF30	-	Very low	Very low
INSUREF40:MVF45	-	Very low	Very low
INSUREF40:MVF50	-	Very low	Very low
INSUREF40:MVPROPHYLACTIC	-	Low	Low
INSUREF40:MVRESCUE_LATE	-	Low	Low
INSUREF40:NEBaAO2less0_22	-	Very low	Very low
INSUREF40:NEBF22_30	-	Low	Low
INSUREF45:INSURELAMCOUNT	-	Very low	Very low
INSUREF45:INSURERESCUE	-	Very low	Very low
INSUREF45:LISAF30	-	Very low	Very low
INSUREF45:LISAF35	-	Very low	Very low
INSUREF45:LISAF40	-	Very low	Very low
INSUREF45:LMACLINICALRD	-	Very low	Very low
INSUREF45:LMAF30	-	Very low	Very low
INSUREF45:LMAF30_60	-	Very low	Very low
INSUREF45:MVF30	-	Very low	Very low
INSUREF45:MVF50	-	Very low	Very low
INSUREF45:MVPROPHYLACTIC	-	Very low	Very low
INSUREF45:MVRESCUE	-	Very low	Very low
INSUREF45:MVRESCUE_LATE	-	Very low	Very low
INSUREF45:NEBaAO2less0_22	-	Very low	Very low
INSUREF45:NEBF22_30	-	Very low	Very low
INSURELAMCOUNT:INSURERESCUE	-	Very low	Very low
INSURELAMCOUNT:LISAF30	-	Very low	Very low
INSURELAMCOUNT:LISAF35	-	Very low	Very low
INSURELAMCOUNT:LISAF40	-	Very low	Very low
INSURELAMCOUNT:LMACLINICALRD	-	Very low	Very low
INSURELAMCOUNT:LMAF30	-	Very low	Very low
INSURELAMCOUNT:LMAF30_60	-	Very low	Very low
INSURELAMCOUNT:MVF30	-	Very low	Very low
INSURELAMCOUNT:MVF45	-	Very low	Very low
INSURELAMCOUNT:MVF50	-	Very low	Very low
INSURELAMCOUNT:MVPROPHYLACTIC	-	Very low	Very low
INSURELAMCOUNT:MVRESCUE	-	Very low	Very low
INSURELAMCOUNT:MVRESCUE_LATE	-	Very low	Very low
INSURELAMCOUNT:NEBaAO2less0_22	-	Very low	Very low
INSURELAMCOUNT:NEBF22_30	-	Very low	Very low
INSURERESCUE:LISAF40	-	Very low	Very low
INSURERESCUE:LMACLINICALRD	-	Very low	Very low
INSURERESCUE:LMAF30	-	Very low	Very low
INSURERESCUE:MVF30	-	Very low	Very low
INSURERESCUE:MVF45	-	Very low	Very low
INSURERESCUE:MVF50	-	Very low	Very low
INSURERESCUE:MVPROPHYLACTIC	-	Very low	Very low
INSURERESCUE:MVRESCUE	-	Very low	Very low
INSURERESCUE:MVRESCUE_LATE	-	Very low	Very low
INSURERESCUE:NEBaAO2less0_22	-	Very low	Very low
INSURERESCUE:NEBF22_30	-	Low	Low
LISAF30:LISAF35	-	Very low	Very low
LISAF30:LISAF40	-	Very low	Very low
LISAF30:LMACLINICALRD	-	Very low	Very low
LISAF30:LMAF30	-	Very low	Very low
LISAF30:LMAF30_60	-	Very low	Very low
LISAF30:MVF30	-	Very low	Very low
LISAF30:MVF50	-	Very low	Very low
LISAF30:MVPROPHYLACTIC	-	Very low	Very low
LISAF30:MVRESCUE	-	Very low	Very low
LISAF30:MVRESCUE_LATE	-	Very low	Very low
LISAF30:NEBaAO2less0_22	-	Very low	Very low
LISAF30:NEBF22_30	-	Low	Low
LISAF35:LISAF40	-	Very low	Very low
LISAF35:LMACLINICALRD	-	Very low	Very low
LISAF35:LMAF30	-	Very low	Very low
LISAF35:LMAF30_60	-	Very low	Very low
LISAF35:MVF30	-	Very low	Very low
LISAF35:MVF45	-	Very low	Very low
LISAF35:MVF50	-	Very low	Very low
LISAF35:MVPROPHYLACTIC	-	Very low	Very low
LISAF35:MVRESCUE	-	Very low	Very low
LISAF35:MVRESCUE_LATE	-	Very low	Very low
LISAF35:NEBaAO2less0_22	-	Very low	Very low
LISAF35:NEBF22_30	-	Very low	Very low
LISAF40:LMACLINICALRD	-	Very low	Very low
LISAF40:LMAF30	-	Very low	Very low
LISAF40:LMAF30_60	-	Very low	Very low
LISAF40:MVF30	-	Very low	Very low
LISAF40:MVF45	-	Very low	Very low
LISAF40:MVF50	-	Very low	Very low
LISAF40:MVPROPHYLACTIC	-	Very low	Very low
LISAF40:MVRESCUE	-	Very low	Very low
LISAF40:MVRESCUE_LATE	-	Very low	Very low
LISAF40:NEBaAO2less0_22	-	Very low	Very low
LISAF40:NEBF22_30	-	Very low	Very low
LMACLINICALRD:LMAF30	-	Low	Low
LMACLINICALRD:LMAF30_60	-	Very low	Very low
LMACLINICALRD:MVF30	-	Very low	Very low
LMACLINICALRD:MVF45	-	Very low	Very low
LMACLINICALRD:MVF50	-	Low	Low
LMACLINICALRD:MVPROPHYLACTIC	-	Low	Low
LMACLINICALRD:MVRESCUE	-	Low	Low
LMACLINICALRD:MVRESCUE_LATE	-	Low	Low
LMACLINICALRD:NEBaAO2less0_22	-	Very low	Very low
LMACLINICALRD:NEBF22_30	-	Very low	Very low
LMAF30:LMAF30_60	-	Very low	Very low
LMAF30:MVF30	-	Very low	Very low
LMAF30:MVF45	-	Very low	Very low
LMAF30:MVF50	-	Very low	Very low
LMAF30:MVPROPHYLACTIC	-	Very low	Very low
LMAF30:MVRESCUE_LATE	-	Very low	Very low
LMAF30:NEBaAO2less0_22	-	Very low	Very low
LMAF30:NEBF22_30	-	Very low	Very low
LMAF30_60:MVF30	-	Very low	Very low
LMAF30_60:MVF45	-	Very low	Very low
LMAF30_60:MVF50	-	Very low	Very low
LMAF30_60:MVPROPHYLACTIC	-	Very low	Very low
LMAF30_60:MVRESCUE	-	Very low	Very low
LMAF30_60:MVRESCUE_LATE	-	Very low	Very low
LMAF30_60:NEBaAO2less0_22	-	Very low	Very low
LMAF30_60:NEBF22_30	-	Very low	Very low
MVF30:MVF45	-	Very low	Very low
MVF30:MVF50	-	Very low	Very low
MVF30:MVPROPHYLACTIC	-	Very low	Very low
MVF30:MVRESCUE	-	Very low	Very low
MVF30:MVRESCUE_LATE	-	Very low	Very low
MVF30:NEBaAO2less0_22	-	Very low	Very low
MVF30:NEBF22_30	-	Very low	Very low
MVF45:MVF50	-	Very low	Very low
MVF45:MVPROPHYLACTIC	-	Very low	Very low
MVF45:MVRESCUE	-	Very low	Very low
MVF45:MVRESCUE_LATE	-	Very low	Very low
MVF45:NEBaAO2less0_22	-	Very low	Very low
MVF50:MVRESCUE	-	Low	Low
MVF50:MVRESCUE_LATE	-	Very low	Very low
MVF50:NEBaAO2less0_22	-	Very low	Very low
MVF50:NEBF22_30	-	Very low	Very low
MVPROPHYLACTIC:MVRESCUE	-	Low	Low
MVPROPHYLACTIC:NEBaAO2less0_22	-	Very low	Very low
MVPROPHYLACTIC:NEBF22_30	-	Very low	Very low
MVRESCUE:NEBaAO2less0_22	-	Very low	Very low
MVRESCUE:NEBF22_30	-	Very low	Very low
MVRESCUE_LATE:NEBaAO2less0_22	-	Very low	Very low
MVRESCUE_LATE:NEBF22_30	-	Very low	Very low
NEBaAO2less0_22:NEBF22_30	-	Very low	Very low
* CoE rated down by one level for inconsistency			

eTable 27: Network characteristics for the outcome of receipt of repeated doses of surfactant in preterm neonates ≤ 36 weeks' gestation

comparison	n.studies	n.patients	n.outcomes	proportion
INRECSUREF30 vs. INSUREF30	1	218	102	0.46788991
INSURECLINICALRD vs. INSUREF40	1	159	10	0.06289308
INSURECLINICALRD vs. LMACLINICALRD	1	50	1	0.02000000
INSURECLINICALRD vs. MVRESCUE	1	278	52	0.18705036
INSUREF22_30 vs. MVRESCUE	1	105	52	0.49523810
INSUREF30 vs. LISAF30	5	616	81	0.13149351
INSUREF30 vs. LMAF30	1	70	5	0.07142857
INSUREF30 vs. MVF30	1	27	7	0.25925926
INSUREF40 vs. LISAF40	5	739	102	0.13802436
INSUREF40 vs. MVRESCUE	1	45	4	0.08888889
LMAF30 vs. MVRESCUE	1	103	29	0.28155340

Characteristic	Value
Number of Interventions	11
Number of Studies	19
Total Number of Patients in Network	2410
Total Possible Pairwise Comparisons	55
Total Number of Pairwise Comparisons With Direct Data	11
Is the network connected?	TRUE
Number of Two-arm Studies	19
Number of Multi-Arms Studies	0
Total Number of Events in Network	445
Number of Studies With No Zero Events	17
Number of Studies With At Least One Zero Event	2
Number of Studies with All Zero Events	0

eTable 28: Matrix plot depicting the network estimates for the outcome of receipt of repeated doses of surfactant in preterm neonates ≤ 36 weeks' gestation

INRECSUREF30	0.2 (0.02, 2.21)	0.19 (0.01, 1.98)	1.27 (0.65, 2.51)	0.13 (0.01, 1.79)	1.41 (0.65, 3.35)	0.16 (0.01, 2.26)	*	0.8 (0.08, 6.09)	#	0.38 (0.03, 3.58)
4.94 (0.45, 62.28)	INSURECLINICALRD	0.93 (0.3, 2.52)	6.24 (0.63, 73.24)	0.66 (0.18, 2.16)	6.98 (0.69, 86.38)	0.79 (0.2, 2.85)	#	3.9 (1.16, 12.42)	#	1.9 (0.84, 3.89)
5.34 (0.5, 68.97)	1.07 (0.4, 3.36)	INSUREF22_30	6.81 (0.71, 80.49)	0.71 (0.15, 3.26)	7.58 (0.77, 95.38)	0.85 (0.17, 4.23)	#	4.21 (1.33, 13.88)	#	2.04 (0.98, 4.38)
0.79 (0.4, 1.55)	0.16 (0.01, 1.58)	0.15 (0.01, 1.41)	INSUREF30	0.1 (0.01, 1.29)	1.11 (0.7, 1.84)	0.12 (0.01, 1.63)	*	0.63 (0.07, 4.28)	#	0.3 (0.03, 2.55)
7.63 (0.56, 119.9)	1.52 (0.46, 5.48)	1.41 (0.31, 6.5)	9.67 (0.77, 142.55)	INSUREF40	10.78 (0.84, 167.47)	1.2 (0.76, 1.93)	#	5.95 (1.21, 30.07)	#	2.88 (0.78, 11.22)
0.71 (0.3, 1.55)	0.14 (0.01, 1.46)	0.13 (0.01, 1.3)	0.9 (0.54, 1.42)	0.09 (0.01, 1.19)	LISAF30	0.11 (0.01, 1.5)	*	0.56 (0.06, 4.04)	#	0.27 (0.02, 2.38)
6.34 (0.44, 103.88)	1.27 (0.35, 4.92)	1.18 (0.24, 5.76)	8.05 (0.61, 123.63)	0.83 (0.52, 1.32)	8.96 (0.67, 144.06)	LISAF40	#	4.94 (0.94, 26.52)	#	2.4 (0.59, 10)
0 (0, 4.14)	0 (0, 0.62)	0 (0, 0.6)	0 (0, 5.22)	0 (0, 0.45)	0 (0, 5.88)	0 (0, 0.54)	LMACLINICALRD	0 (0, 2.55)	*	0 (0, 1.19)
1.26 (0.16, 12.34)	0.26 (0.08, 0.86)	0.24 (0.07, 0.75)	1.59 (0.23, 14.33)	0.17 (0.03, 0.83)	1.78 (0.25, 16.94)	0.2 (0.04, 1.07)	*	LMAF30	#	0.49 (0.19, 1.17)
0 (0, 0.04)	0 (0, 0.01)	0 (0, 0.01)	0 (0, 0.05)	0 (0, 0.01)	0 (0, 0.06)	0 (0, 0.01)	*	0 (0, 0.04)	MVF30	0 (0, 0.02)
2.6 (0.28, 30.14)	0.53 (0.26, 1.19)	0.49 (0.23, 1.02)	3.3 (0.39, 35.22)	0.35 (0.09, 1.29)	3.69 (0.42, 41.6)	0.42 (0.1, 1.69)	*	2.05 (0.86, 5.22)	#	MVRESCUE

eTable 29: CoE for various comparisons for the outcome of receipt of repeated doses of surfactant in preterm neonates ≤ 36 weeks' gestation

	CoE from direct evidence	CoE from indirect evidence	CoE from network estimate
**INRECSUREF30:INSUREF30**	Low	-	Low
**INSURECLINICALRD:LMACLINICALR**	Low	-	Low
**INSUREF22_30:MVRESCUE**	Low	-	Low
**INSUREF30:LISAF30**	Moderate	-	Moderate
**INSUREF30:LMAF30**	Low	-	Low
**INSUREF30:MVF30**	Moderate	-	Moderate
**INSUREF40:LISAF40**	Low	-	Low
**LMAF30:MVRESCUE**	Moderate	-	Moderate
**INSURERESCUE:LISAF30**	Low	-	Low
**INSURECLINICALRD:MVRESCUE**	Moderate	Very low	Moderate
**INSUREF40:MVRESCUE**	Very low	Very low	Very low
**INSURECLINICALRD:INSUREF40**	Very low	Very low	Very low
**INRECSUREF30:INSURECLINICALRD**	-	Low	Low
**INRECSUREF30:INSUREF22_30**	-	Low	Low
**INRECSUREF30:INSUREF40**	-	Very low	Very low
**INRECSUREF30:LISAF30**	-	Low	Low
**INRECSUREF30:LISAF40**	-	Very low	Very low
**INRECSUREF30:LMACLINICALRD**	-	Low	Low
**INRECSUREF30:LMAF30**	-	Low	Low
**INRECSUREF30:MVF30**	-	Low	Low
**INRECSUREF30:MVRESCUE**	-	Low	Low
**INSURECLINICALRD:INSUREF22_30**	-	Low	Low
**INSURECLINICALRD:INSUREF30**	-	Low	Low
**INSURECLINICALRD:LISAF30**	-	Low	Low
**INSURECLINICALRD:LISAF40**	-	Very low	Very low
**INSURECLINICALRD:LMAF30**	-	Moderate	Moderate
**INSURECLINICALRD:MVF30**	-	Very low	Very low
**INSUREF22_30:INSUREF30**	-	Very low	Very low
**INSUREF22_30:INSUREF40**	-	Very low	Very low
**INSUREF22_30:LISAF30**	-	Low	Low
**INSUREF22_30:LISAF40**	-	Very low	Very low
**INSUREF22_30:LMACLINICALRD**	-	Low	Low
**INSUREF22_30:LMAF30**	-	Low	Low
**INSUREF22_30:MVF30**	-	Low	Low
**INSUREF30:INSUREF40**	-	Very low	Very low
**INSUREF30:LISAF40**	-	Very low	Very low
**INSUREF30:LMACLINICALRD**	-	Very low	Very low
**INSUREF30:MVRESCUE**	-	Low	Low
**INSUREF40:LISAF30**	-	Very low	Very low
**INSUREF40:LMACLINICALRD**	-	Very low	Very low
**INSUREF40:LMAF30**	-	Very low	Very low
**INSUREF40:MVF30**	-	Very low	Very low
**LISAF30:LISAF40**	-	Very low	Very low
**LISAF30:LMACLINICALRD**	-	Very low	Very low
**LISAF30:LMAF30**	-	Low	Low
**LISAF30:MVF30**	-	Very low	Very low
**LISAF30:MVRESCUE**	-	Very low	Very low
**LISAF40:LMACLINICALRD**	-	Very low	Very low
**LISAF40:LMAF30**	-	Very low	Very low
**LISAF40:MVF30**	-	Very low	Very low
**LISAF40:MVRESCUE**	-	Very low	Very low
**LMACLINICALRD:LMAF30**	-	Low	Low
**LMACLINICALRD:MVF30**	-	Low	Low
**LMACLINICALRD:MVRESCUE**	-	Low	Low
**LMAF30:MVF30**	-	Low	Low
**MVF30:MVRESCUE**	-	Low	Low

Annexure 1: Interventions evaluated in the network meta-analysis

33 interventions were evaluated in this NMA. Each intervention had two components: the modality utilised for surfactant administration and the decision threshold at which surfactant replacement therapy was given. The acronyms used for the modality utilised is provided below. FiO_2 thresholds are depicted using the letter 'F' followed by the fraction which was expressed as percentage. For example if the threshold used was FiO_2 of 0.40, it was expressed as F40. The acronyms used for the modality of surfactant administration and other thresholds besides FiO2 are explained below.

Abbreviation	Explanation
aAO2less0_36	Arterial alveolar oxygen tension ratio of less than 0.36
aAO2less0_22	Arterial alveolar oxygen tension ratio of less than 0.36
MV_RESCUE	Surfactant administration followed by continued mechanical ventilation when respiratory failure was diagnosed based on blood gas parameters of pH, partial pressure of oxygen and carbon dioxide. Some authors had also used differing criteria of FiO_2 and repeated episodes of apnea to define respiratory failure.
MVRESCUE_LATE	The definition of MVRESCUE was same as mentioned above. When surfactant was administered in a neonate who was mechanically ventilated when respiratory failure was diagnosed and also required a particular level of mean airway pressure and FiO_2, the intervention was classified as MVRESCUE_LATE.
MVPROPHYLACTIC	Surfactant administration immediately after birth before the symptoms of RDS appeared and when the neonate was intubated solely for the purpose of surfactant administration.
MVF	Surfactant administration based on different FiO_2 cut offs followed by continued mechanical ventilation beyond 1 hour.
MVCLINICALRD	Surfactant administration based on clinical assessment of respiratory distress followed by continued mechanical ventilation beyond 1 hour.
CPAP_only	The neonate was managed on only CPAP without any surfactant administration.
INSURE	Surfactant administration by Intubate-Surfactant-Extubation modality. The authors could have extubated at different time points but should not have continued mechanical ventilation beyond 1 hour.
INRECSURE	When manoeuvres such as sustained inflation along with conventional ventilation or recruitment through HFOV was administered prior to surfactant administration by INSURE
INSURELAMCOUNT	Surfactant administration by INSURE method based on lamellar body counts obtained by centrifuged sample of gastric aspirates.
INSURERESCUE	Rescue (as defined above) surfactant administration by INSURE
LISA	Less invasive surfactant administration while the neonate is supported on a non-invasive respiratory support modality such as CPAP using different types of catheters, without the insertion of an endotracheal tube.
LISA_LUS	Surfactant administration by LISA guided by lung ultrasound assessment of severity of RDS.
LMA	Laryngeal mask airway

Annexure 2: Narrative review of the included the studies in the systematic review [54-58]

Five studies were included in the narrative review. Of these, 4 evaluated long term neurodevelopmental outcomes of preterm neonates with RDS who were treated with surfactant using different modalities and varying thresholds; 1 study assessed the efficacy of delivery room CPAP and INSURE strategy vs. oxygen administration followed by mechanical ventilation in preterm neonates with RDS.

Vaucher et al. in their follow up study of very low birth weight (VLBW) neonates enrolled in a 3-armed RCT reported that at 12 months' corrected age (CA) both the Mental Development Index (MDI) (78 vs 87, p=0.02) and Psychomotor Development Index (PDI) (73 vs. 87, p=0.04) were lower in the prophylactic surfactant group when compared to the late rescue group. Both these groups were continued on invasive mechanical ventilation after surfactant administration. The third group in this study received placebo. Hentschel et al. in their follow up study of preterm neonates born at 27-32 weeks noted that both the neurobehavioral and motor development as assessed by Griffiths test were comparable at 20 months' CA in preterm neonates with RDS who received surfactant as early rescue vs. late rescue followed by continued mechanical ventilation. Certain sub scales such as 'personal social' (p=0.02), 'abnormal muscle tone' (p=0.01) and 'rolling over from supine to prone' (p=0.01) was noted to be delayed in the early rescue group.

Herting et al. and Mehler et al. evaluated the outcomes of neonates who received LISA at 24 months' corrected age. While Herting et al. did not find any difference in the anthropological parameters or neurodevelopmental outcomes as assessed by Bayley II scores, Mehler et at. noted that lesser proportion of preterm neonates with RDS who were treated with LISA had PDI (p=0.01) and MDI <70 (p=0.00) at 24 months' CA, when compared to those who required endotracheal intubation.

Tapia et al. conducted a randomised controlled trial evaluating the effect of delivery room CPAP vs. oxygen administration in VLBW neonates. While the CPAP group received surfactant by INSURE method at an FiO_2 of 35%, the oxygen hood group received surfactant followed by mechanical ventilation at a similar FiO_2 cut-off. It was reported that the CPAP/ INSURE group had a lower risk of requirement of invasive mechanical ventilation (p=0.00) as well as surfactant (p=0.00) when compared to the oxygen / mechanical ventilation group.

REFERENCES

1. Verder H., Albertsen P., Ebbesen F., Greisen G., Robertson B., Bertelsen A., et al. Nasal continuous positive airway pressure and early surfactant therapy for respiratory distress syndrome in newborns of less than 30 weeks' gestation. Pediatrics. 1999;103(2):E24.
2. Verder H, Ebbesen F, Fenger-Gron J, Henriksen TB, Andreasson B, Bender L, et al. Early surfactant guided by lamellar body counts on gastric aspirate in very preterm infants. Neonatology. 2013;104(2):116–22.
3. Rodriguez-Fanjul J, Jordan I, Balaguer M, Batista-Munoz A, Ramon M, Bobillo-Perez S. Early surfactant replacement guided by lung ultrasound in preterm newborns with RDS: the ULTRASURF randomised controlled trial. Eur J Pediatr. 2020;179(12):1913–20.
4. Attridge JT, Stewart C, Stukenborg GJ, Kattwinkel J. Administration of rescue surfactant by laryngeal mask airway: lessons from a pilot trial. Am J Perinatol. 2013;30(3):201–6.
5. Bao Y, Zhang G, Wu M, Ma L, Zhu J. A pilot study of less invasive surfactant administration in very preterm infants in a Chinese tertiary center. BMC Pediatr. 2015;15(100967804):21.
6. Barbosa RF, Simoes E Silva AC, Silva YP. A randomized controlled trial of the laryngeal mask airway for surfactant administration in neonates. J Pediatr (Rio J). 2017;93(4):343–50.
7. Boskabadi H, Maamouri G, Gharaei Jomeh R, Zakerihamidi M. Comparative study of the effect of the administration of surfactant through a thin endotracheal catheter into trachea during spontaneous breathing with intubation (intubation-surfactant-extubation method). J Clin Neonatol. 2019;8(4):227-231.
8. Choupani R, Mashayekhy G, Hmidi M, Kheiri S, Dehkordi M. A Comparative study of the efficacy of surfactant administration through a thin intratracheal catheter and its administration via an endotracheal tube in neonatal respiratory distress syndrome. Iran J Neonatol. 2018;9(4):33-40.
9. Dani C., Bertini G., Pezzati M., Cecchi A., Caviglioli C., Rubaltelli F.F. Early extubation and nasal continuous positive airway pressure after surfactant treatment for respiratory distress syndrome among preterm infants <30 weeks' gestation. Pediatrics. 2004;113(6):e560-563.
10. Dargaville PA, Kamlin COF, Orsini F, Wang X, De Paoli AG, Kanmaz Kutman HG, et al. Effect of Minimally Invasive Surfactant Therapy vs

Sham Treatment on Death or Bronchopulmonary Dysplasia in Preterm Infants With Respiratory Distress Syndrome: The OPTIMIST-A Randomized Clinical Trial. JAMA J Am Med Assoc. 2021;326(24):2478–87.
11. Dilmen U., Ozdemir R., Tatar Aksoy H., Uras N., Demirel N., Kirimi E., et al. Early regular versus late selective poractant treatment in preterm infants born between 25 and 30 gestational weeks: A prospective randomized multicenter study. J Matern Fetal Neonatal Med. 2014;27(4):411–5.
12. Dunn MS, Shennan AT, Zayack D, Possmayer F. Bovine surfactant replacement therapy in neonates of less than 30 weeks' gestation: a randomized controlled trial of prophylaxis versus treatment. Pediatrics. 1991;87(3):377–86.
13. Egberts J, de Winter JP, Sedin G, de Kleine MJ, Broberger U, van Bel F, et al. Comparison of prophylaxis and rescue treatment with Curosurf in neonates less than 30 weeks' gestation: a randomized trial. Pediatrics. 1993;92(6):768–74.
14. Gharehbaghi M, Moghaddam Y, Radfar R. Comparing the efficacy of surfactant administration by laryngeal mask airway and endotracheal intubation in neonatal respiratory distress syndrome. Crescent J Med Biol Sci. 2018;5(3):222-227.
15. Gopel W, Kribs A, Ziegler A, Laux R, Hoehn T, Wieg C, et al. Avoidance of mechanical ventilation by surfactant treatment of spontaneously breathing preterm infants (AMV): an open-label, randomised, controlled trial. Lancet Lond Engl. 2011;378(9803):1627–34.
16. Gortner L, Wauer RR, Hammer H, Stock GJ, Heitmann F, Reiter HL, et al. Early versus late surfactant treatment in preterm infants of 27 to 32 weeks' gestational age: a multicenter controlled clinical trial. Pediatrics. 1998;102(5):1153–60.
17. Huang XL, Chen D, Li XP, Li MY, Shen JF, Wu XS. [Clinical effectiveness of INSURE method in the treatment of neonatal respiratory distress syndrome]. Zhongguo Dang Dai Er Ke Za Zhi Chin J Contemp Pediatr. 2013;15(1):9–13.
18. Imani M., Derafshi R., Arbabisarjou A. Comparison of nasal continuous positive airway pressure therapy with and without prophylactic surfactant in preterm neonates. Intensive Care Med. 2013;39:S138.
19. Kandraju H, Murki S, Subramanian S, Gaddam P, Deorari A, Kumar P. Early routine versus late selective surfactant in preterm neonates with

respiratory distress syndrome on nasal continuous positive airway pressure: a randomized controlled trial. Neonatology. 2013;103(2):148–54.
20. Kendig J, Notter R, Cox C, Reubens L, Davis J, Maniscalco W, et al. A comparison of surfactant as immediate prophylaxis and as rescue therapy in newborns of less than 30 weeks' gestation. N Engl J Med. 1991;324(13):865-871.
21. Khosravi N, Mohagheghi P. Do large preterm infants with respiratory distress syndrome benefit from early surfactant? Acta Med Iran. 2008;46(5):391-394.
22. Konishi M, Fujiwara T, Chida S, Maeta H, Shimada S, Kasai T, et al. A prospective, randomized trial of early versus late administration of a single dose of surfactant-TA. Early Hum Dev. 1992;29(1):275–82.
23. Li XF, Cheng TT, Guan RL, Liang H, Lu WN, Zhang JH, et al. Effects of different surfactant administrations on cerebral autoregulation in preterm infants with respiratory distress syndrome. J Huazhong Univ Sci Technol Med Sci Hua Zhong Ke Ji Xue Xue Bao Yi Xue Ying Wen Ban Huazhong Keji Daxue Xuebao Yixue Yingdewen Ban. 2016;36(6):801–5.
24. Lefort S, Diniz EMA, Vaz FAC. Clinical course of premature infants intubated in the delivery room, submitted or not to porcine-derived lung surfactant therapy within the first hour of life. J Matern-Fetal Neonatal Med Off J Eur Assoc Perinat Med Fed Asia Ocean Perinat Soc Int Soc Perinat Obstet. 2003;14(3):187–96.
25. Nayeri FS, Esmaeilnia Shirvani T, Aminnezhad M, Amini E, Dalili H, Moghimpour Bijani F. Comparison of INSURE method with conventional mechanical ventilation after surfactant administration in preterm infants with respiratory distress syndrome: therapeutic challenge. Acta Med Iran. 2014;52(8):596–600.
26. Olivier F, Nadeau S, Belanger S, Julien AS, Masse E, Ali N, et al. Efficacy of minimally invasive surfactant therapy in moderate and late preterm infants: A multicentre randomized control trial. Paediatr Child Health. 2017;22(3):120–4.
27. Anonymous. Early versus delayed neonatal administration of a synthetic surfactant--the judgment of OSIRIS. The OSIRIS Collaborative Group (open study of infants at high risk of or with respiratory insufficiency--the role of surfactant. Lancet Lond Engl. 1992;340(8832):1363–9.
28. Sabzehei MK, Basiri B, Shokouhi M, Ghremani S, Moradi A. Comparison of minimally invasive surfactant therapy with intubation

surfactant administration and extubation for treating preterm infants with respiratory distress syndrome: a randomized clinical trial. Clin Exp Pediatr. 2021;(101761234).
29. Sadeghnia A, Tanhaei M, Mohammadizadeh M, Nemati M. A comparison of surfactant administration through i-gel and ET-tube in the treatment of respiratory distress syndrome in newborns weighing more than 2000 grams. Adv Biomed Res. 2014;3(101586897):160.
30. Finer N.N., Carlo W.A., Walsh M.C., Rich W., Gantz M.G., Laptook A.R., et al. Early CPAP versus surfactant in extremely preterm infants. N Engl J Med. 2010;362(21):1970–9.
31. 2004. Early surfactant for neonates with mild to moderate respiratory distress syndrome: a multicenter randomized trial. J Pediatr. 2004;144:804-808.
32. Yang G, Hei M, Xue Z, Zhao Y, Zhang X, Wang C. Effects of less invasive surfactant administration (LISA) via a gastric tube on the treatment of respiratory distress syndrome in premature infants aged 32 to 36 weeks. Medicine (Baltimore). 2020;99(9):e19216.
33. Amini E., Sheikh M., Shariat M., Dalili H., Azadi N., Nourollahi S. Surfactant administration in preterm neonates using laryngeal mask airway: A randomized clinical trial. Acta Med Iran. 2019;57(6):348–54.
34. Berggren E, Liljedahl M, Winbladh B, Andreasson B, Curstedt T, Robertson B, et al. Pilot study of nebulized surfactant therapy for neonatal respiratory distress syndrome. Acta Paediatr Oslo Nor 1992. 2000;89(4):460–4.
35. Gallup J, Pinheiro J, Ndakor S, Pezzano C. Randomized trial of surfactant therapy via laryngeal mask airway vs. Brief tracheal intubation. Pediatrics. 2021;147(3):755-756.
36. Gupta B.K., Saha A.K., Mukherjee S., Saha B. Minimally invasive surfactant therapy versus InSurE in preterm neonates of 28 to 34 weeks with respiratory distress syndrome on non-invasive positive pressure ventilation-a randomized controlled trial. Eur J Pediatr. 2020;179(8):1287–93.
37. Han T, Liu H, Zhang H, Guo M, Zhang X, Duan Y, et al. Minimally Invasive Surfactant Administration for the Treatment of Neonatal Respiratory Distress Syndrome: A Multicenter Randomized Study in China. Front Pediatr. 2020;8(101615492):182.
38. Heidarzadeh M, Mirnia K, Hoseini M, Sadeghnia A, Akrami F, Balila M, et al. Surfactant administration via thin catheter during spontaneous

breathing: randomized controlled trial in alzahra hospital. Iran J Neonatol. 2013;4(2):5-9.
39. Jena SR, Bains HS, Pandita A, Verma A, Gupta V, Kallem VR, et al. Surfactant therapy in premature babies: SurE or InSurE. Pediatr Pulmonol. 2019;54(11):1747–52.
40. Kanmaz HG, Erdeve O, Canpolat FE, Mutlu B, Dilmen U. Surfactant administration via thin catheter during spontaneous breathing: randomized controlled trial. Pediatrics. 2013;131(2):e502-9.
41. Kribs A., Roll C., Gopel W., Wieg C., Groneck P., Laux R., et al. Nonintubated surfactant application vs conventional therapy in extremely preterm infants: A randomized clinical trial. JAMA Pediatr. 2015;169(8):723–30.
42. Merritt T, Hallman M, Berry C, Pohjavuori M, Edwards D, Jaaskelainen J, et al. Randomized, placebo-controlled trial of human surfactant given at birth versus rescue administration in very low birth weight infants with lung immaturity. J Pediatr. 1991;118(4):581-594.
43. Minocchieri S, Berry CA, Pillow JJ, CureNeb Study Team. Nebulised surfactant to reduce severity of respiratory distress: a blinded, parallel, randomised controlled trial. Arch Dis Child Fetal Neonatal Ed. 2019;104(3):F313–9.
44. Pareek P, Deshpande S, Suryawanshi P, Sah LK, Chetan C, Maheshwari R, et al. Less Invasive Surfactant Administration (LISA) vs. Intubation Surfactant Extubation (InSurE) in Preterm Infants with Respiratory Distress Syndrome: A Pilot Randomized Controlled Trial. J Trop Pediatr. 2021;67(4).
45. Pinheiro JMB, Santana-Rivas Q, Pezzano C. Randomized trial of laryngeal mask airway versus endotracheal intubation for surfactant delivery. J Perinatol Off J Calif Perinat Assoc. 2016;36(3):196–201.
46. Reininger A, Khalak R, Kendig JW, Ryan RM, Stevens TP, Reubens L, et al. Surfactant administration by transient intubation in infants 29 to 35 weeks' gestation with respiratory distress syndrome decreases the likelihood of later mechanical ventilation: a randomized controlled trial. J Perinatol. 2005;25(11):703–8.
47. Roberts KD, Brown R, Lampland AL, Leone TA, Rudser KD, Finer NN, et al. Laryngeal Mask Airway for Surfactant Administration in Neonates: A Randomized, Controlled Trial. J Pediatr. 2018;193:40-46.e1.
48. Rojas MA, Lozano JM, Rojas MX, Laughon M, Bose CL, Rondon MA, et al. Very early surfactant without mandatory ventilation in premature infants treated with early continuous positive airway

pressure: a randomized, controlled trial. Pediatrics. 2009;123(1):137–42.
49. Vento G, Ventura ML, Pastorino R, van Kaam AH, Carnielli V, Cools F, et al. Lung recruitment before surfactant administration in extremely preterm neonates with respiratory distress syndrome (IN-REC-SUR-E): a randomised, unblinded, controlled trial. Lancet Respir Med. 2021;9(2):159–66.
50. Verder H, Robertson B, Greisen G, Ebbesen F, Albertsen P, Lundstrom K, et al. Surfactant therapy and nasal continuous positive airway pressure for newborns with respiratory distress syndrome. Danish-Swedish Multicenter Study Group. N Engl J Med. 1994;331(16):1051–5.
51. Walti H, Paris-Llado J, Bréart G, Couchard M. Porcine surfactant replacement therapy in newborns of 25-31 weeks' gestation: a randomized, multicentre trial of prophylaxis versus rescue with multiple low doses. The French Collaborative Multicentre Study Group. Acta Paediatr. 1995;84(8):913-921.
52. Yang Y, Yan W, Ruan M, Zhang L, Su J, Deng H, et al. Lung recruitment improves the efficacy of intubation-surfactant-extubation treatment for respiratory distress syndrome in preterm neonates, a randomized controlled trial. BMC Pediatr. 2022;22(1):1–8.
53. Mohammadizadeh M, Ardestani A, Sadeghnia A. Early administration of surfactant via a thin intratracheal catheter in preterm infants with respiratory distress syndrome: feasibility and outcome. J Res Pharm Pract. 2015;4(1):31-36.
54. Hentschel R, Dittrich F, Hilgendorff A, Wauer R, Westmeier M, Gortner L. Neurodevelopmental outcome and pulmonary morbidity two years after early versus late surfactant treatment: does it really differ?. Acta Paediatr Oslo Nor 1992. 2009;98(4):654–9.
55. Tapia JL, Urzua S, Bancalari A, Meritano J, Torres G, Fabres J, et al. Randomized trial of early bubble continuous positive airway pressure for very low birth weight infants. J Pediatr. 2012;161(1):75-80.e1.
56. Vaucher YE, Harker L, Merritt TA, Hallman M, Gist K, Bejar R, et al. Outcome at twelve months of adjusted age in very low birth weight infants with lung immaturity: a randomized, placebo-controlled trial of human surfactant. J Pediatr. 1993;122(1):126–32.
57. Herting E, Kribs A, Hartel C, von der Wense A, Weller U, Hoehn T, et al. Two-year outcome data suggest that less invasive surfactant administration (LISA) is safe. Results from the follow-up of the

randomized controlled AMV (avoid mechanical ventilation) study. Eur J Pediatr. 2020;179(8):1309–13.
58. Mehler K., Broer A., Roll C., Gopel W., Wieg C., Jahn P., et al. Developmental outcome of extremely preterm infants is improved after less invasive surfactant application: Developmental outcome after LISA. Acta Paediatr Int J Paediatr. 2021;110(3):818–25.
